# Controlling Magnetic Anisotropy of Endohedral Lanthanide Ions by Carbene Addition: Paramagnetic NMR, Lanthanide Luminescence, and Single-Molecule Magnetism in Adamantylidene Adducts of MSc_2_N@C_80_ (M = Nd, Dy)

**DOI:** 10.1021/jacs.5c10147

**Published:** 2025-09-03

**Authors:** Wei Yang, Matheus Felipe de Souza Barbosa, Noel Israel, Marco Rosenkranz, Fupin Liu, Stanislav M. Avdoshenko, Alexey A. Popov

**Affiliations:** † 28394 Leibniz Institute for Solid State and Materials Research (IFW Dresden), Helmholtzstr. 20, Dresden 01069, Germany; ‡ Jiangsu Key Laboratory of New Power Batteries, Jiangsu Collaborative Innovation Center of Biomedical Functional Materials, School of Chemistry and Materials Science, 12534 Nanjing Normal University, Nanjing 210023, China

## Abstract

Metallofullerenes with endohedral lanthanides have emerged as a versatile class of single-molecule magnets owing to strong single-ion magnetic anisotropy, which can be realized in the interior of the fullerene cage. Since exohedral chemical modification of fullerenes is often used to adjust their properties and processability for prospective practical applications, it is necessary to understand how it can affect their magnetic properties. In this work, we studied how a popular [2 + 1] cycloaddition reaction, photochemical addition of adamantylidene (Ad), affects single-ion magnetic anisotropy and single-molecule magnetism of MSc_2_N@C_80_ (M = Nd, Dy). For each lanthanide, the reaction yielded [5,6]-open and [6,6]-open isomers of the monoadduct MSc_2_N@C_80_(Ad). Paramagnetic ^1^H NMR was demonstrated that the Ad-addition site in [5,6] isomers is predominantly coordinated by Sc, whereas both Sc and lanthanide coordination coexist in [6,6] isomers. *Ab initio* calculations and Nd-based photoluminescence showed that the [5,6] isomer has enhanced ligand-field splitting, whereas coordination of the lanthanide to the Ad-addition site in the [6,6] isomer reduces magnetic axiality and ligand-field splitting. For DySc_2_N@C_80_(Ad), Dy-Ad coordination leads to a noticeable reduction in the blocking temperature of magnetization, whereas Dy coordination to the unfunctionalized fragments of the fullerene cage improves the SMM performance in comparison to the unfunctionalized DySc_2_N@C_80_. Thus, carbene addition can enhance or deteriorate SMM properties depending on the regioisomerism and the lanthanide-cage coordination geometry in MSc_2_N@C_80_(Ad) adducts. These results demonstrate that chemical derivatization of EMFs can become a useful tool for improving their magnetic properties, but will require careful evaluation of different factors for each reaction type.

## Graphical abstract

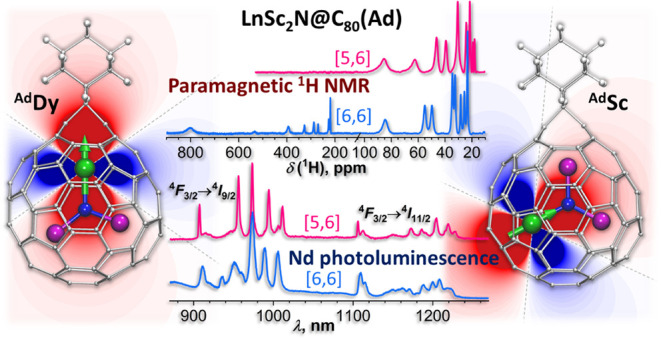

## Introduction

Endohedral metallofullerenes (EMFs) form a special class of molecules in which a carbon cage encapsulates one to several metal and nonmetal atoms, often in quite exotic constellations, yet with atomically precise composition.^1–7^ This turns EMFs into a platform for the study of unusual physical properties emerging in endohedral clusters due to their unconventional structures and electronic states. For lanthanide EMFs, one such property is magnetic anisotropy, which can be quite strong in EMFs owing to the short interatomic distances and large atomic charges.^8–10^ Strong magnetic anisotropy induced in endohedral lanthanide ions leads to single-molecule magnetism (SMM),^11–15^ which among EMFs was first discovered in 2012 for DySc_2_N@C_80_ ^16^ and has since been observed in different types of Dy-EMFs,^10,17–29^ as well as in some EMFs with other lanthanides, such as Pr,^30^ Nd,^31,32^ Tb,^33–39^ and Ho.^33,40^ Encapsulation of lanthanide atoms inside chemically and thermally stable fullerene cages is an important advantage of EMFs because it allows stabilization of unusual species in the interior of the carbon cage and, at the same time, dramatically simplifies processing of EMF-SMMs necessary for the exploration and utilization of their genuinely single-molecule spin properties.^41–46^

Strong intracluster interactions are the main source of the ligand field and magnetic anisotropy in lanthanide EMFs, and for a decade since 2012, the design of EMF-SMMs has been mainly focused on intracluster effects. But the fullerene host is also noninnocent in this regard, and the role of the carbon cage has recently become a more active area of research.^23,25,31,47–49^ It is already understood that lanthanide-fullerene bonding induces a nonaxial contribution to the ligand field, which is potentially harmful to SMM performance, but the effect can vary significantly with alteration of the metal-cage coordination geometry.^25,49^ Another important factor for SMMs is spin-phonon coupling,^50–52^ and here rigid fullerene cages fortuitously provide a phonon-poor environment. The excellent SMM performance of Dy@C_81_N, despite its modest single-ion anisotropy, is believed to stem from the minimal number of low-frequency vibrations in this molecule.^48^ Relaxation of magnetization in EMFs is likely driven by metal-based vibrations,^52^ which are inevitably affected by the metal-cage bonding.^48,53^ Any modification of the fullerene cage, for instance, by its chemical derivatization, should also affect metal-cage interactions and, hence, the magnetic properties of EMFs.

Chemical modification of fullerenes is often required to adjust their physicochemical properties, including solubility and molecular orbital energies, or to introduce different functional groups for prospective practical applications. More than three decades of EMF research have demonstrated that the fullerene cage has rich chemistry and provided multiple examples of different reaction types, particularly numerous variants of cycloaddition.^54–58^ Cycloaddition across a fullerene C–C bond offers two conceptually different possibilities. One is that the two affected fullerene atoms change their hybridization from C-sp^2^ to C-sp^3^, while the σ-bond between them is preserved. Typical examples are 1,3-dipolar cycloaddition (Prato reaction)^59,60^ or Diels–Alder reaction.^61^ Endohedral metals usually avoid cage C-sp^3^ atoms in such adducts. Another possibility is that the C-sp^2^ hybridization of the cage atoms is preserved, whereas the σ-bond between them is broken. A typical example is [2 + 1] cycloaddition (cyclopropanation), such as the Bingel-Hirsch reaction,^59,62^ CF_2_ addition,^63,64^ or carbene addition.^65^ A very popular version of the latter is the photochemical reaction with adamantane diaziridine, which gives adamantylidene adducts for many types of EMFs.^66–72^ Carbene addition sites in EMFs with an open C–C bond form a pocket to which endohedral metal atoms tend to coordinate.

In the context of molecular magnetism, 1,3-dipolar cycloaddition was used to introduce surface-anchoring groups to EMF-SMMs Dy_*x*_Sc_3–*x*_N@C_80_ (*x* = 1, 2) and Tb_2_@C_80_(CH_2_Ph) for self-assembly of magnetic monolayers on gold and graphene substrates.^34,43,73^ Prato cycloadducts of EMFs preserved the SMM behavior but altered the blocking temperature of magnetization. Potentially, derivatization might even be employed to improve SMM performance once the influence of this factor is properly understood. However, a possible connection between chemical modification and the magnetic properties of EMFs is currently not well understood and has been scarcely studied.^74^ It is the goal of this work to provide a comprehensive analysis of how the local structural changes of the fullerene cage induced by a typical cycloaddition reaction alter the single-ion anisotropy and SMM properties of endohedral lanthanide ions in nitride cluster fullerene MSc_2_N@C_80_ (M = Nd, Dy). To address this problem, we employ the photochemical reaction of MSc_2_N@C_80_ with AdN_2_ (Figure 1a) and study the obtained MSc_2_N@C_80_(Ad) cycloadducts by a combination of paramagnetic NMR, lanthanide-based photoluminescence, SQUID magnetometry, and quantum-chemical modeling.

**Figure 1:**
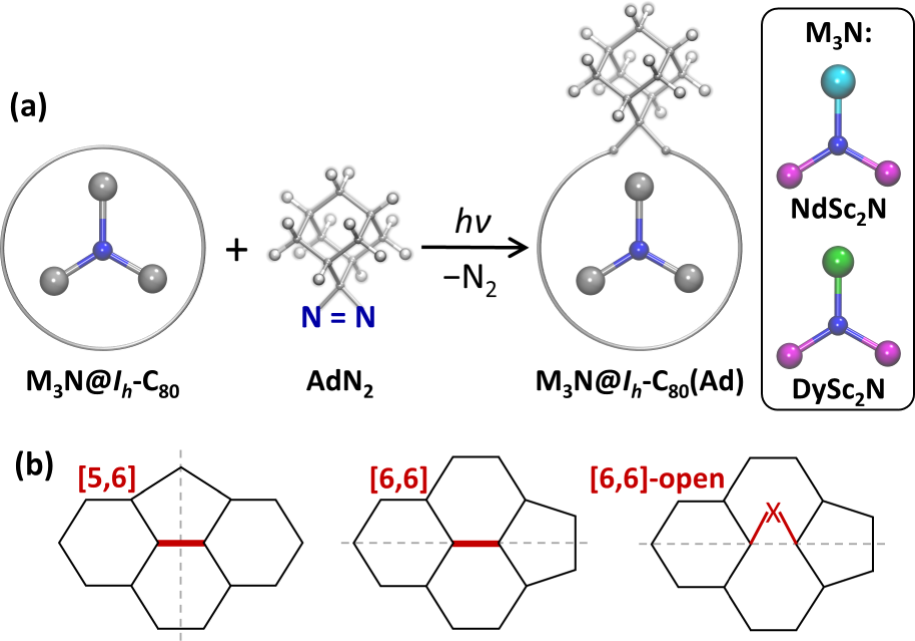
(a) Scheme of the photochemical reaction between M_3_N@C_80_ and AdN_2_ with the formation of the M_3_N@C_80_(Ad) monoadduct; the trimetallic nitride clusters studied in this work are NdSc_2_N and DySc_2_N (Nd – cyan, Dy – green, Sc – magenta, N – blue). (b) [5,6] and [6,6] C–C bonds (highlighted in red) in the *I*_*h*_-C_80_ carbon cage with surrounding pentagons and hexagons, and [6,6]-open adduct; dashed gray lines denote orientations of the symmetry plane.

## Results and Discussion

### Synthesis and Molecular Structure of MSc_2_N@C_80_(Ad) Adducts (M = Nd, Dy)

#### Synthesis of Adamantylidene Adducts

Nitride clusterfullerenes NdSc_2_N@C_80_ and DySc_2_N@C_80_ with *I*_*h*_ fullerene cages were synthesized in our group earlier.^31,75^ Since only *I*_*h*_ isomers are studied in this work, designation of the cage symmetry will be omitted. 2-Adamantane-2,3′-[3H]-diazirine (AdN_2_) was synthesized from commercially available 2-adamantanone following literature methods.^76,77^ Toluene solutions of MSc_2_N@C_80_ mixed with an excess of AdN_2_ were irradiated by a UV-LED light source with maximum intensity at 365 nm (LightningCure LC-L1 from Hamamatsu) to induce the photochemical reaction. HPLC traces measured at indicated time intervals showed depletion of pristine fullerenes (HPLC peaks near 82 min) and the appearance of two peaks at 30–40 min, corresponding to two regio-isomers of MSc_2_N@C_80_(Ad) monoadducts (Figure 2a,b). Formation of bis and poly adducts MSc_2_N@C_80_(Ad)_*n* ≥ 2_ was also identified by the appearance and gradual growth of several overlapping HPLC peaks at shorter times (10–30 min). Since bis-addition did not show the required regioselectivity under these conditions, we focused only on the monoadducts in this work. To avoid excessive formation of poly adducts, irradiation was terminated after 6 min (Nd) and 20 min (Dy) despite incomplete conversion of MSc_2_N@C_80_.

**Figure 2:**
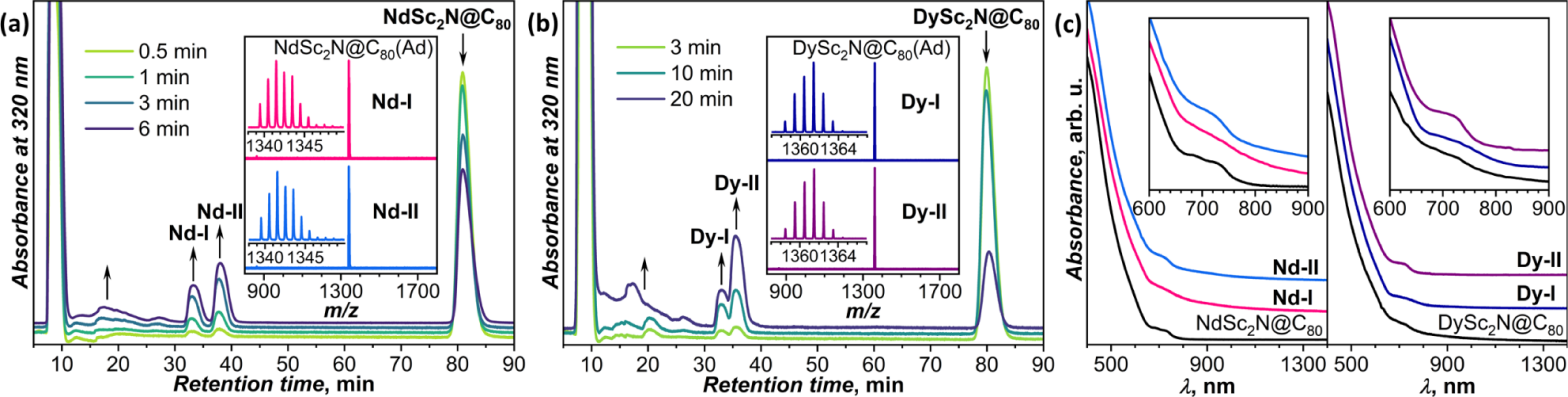
(a, b) HPLC traces measured at different moments of time during the photochemical reaction between MSc_2_N@C_80_ and AdN_2_: (a) M = Nd, (b) M = Dy. Insets show MALDI mass spectra of two isolated isomers of MSc_2_N@C_80_(Ad), denoted as **M-I** and **M-II** (M = Nd, Dy). (c) Vis-NIR absorption spectra of MSc_2_N@C_80_, **M-I**, and **M-II** (M = Nd, Dy) in CS_2_ solution.

After the reaction, isomers of MSc_2_N@C_80_(Ad) monoadducts were separated by HPLC and, for DySc_2_N@C_80_(Ad), additionally purified with recycling HPLC to give two isomers for each lanthanide (see Supporting Information for further details). Their compositions were verified by MALDI-TOF mass spectrometry, in which molecular peaks of MSc_2_N@C_80_(Ad) were observed without fragmentation (insets in Figures 2a,b and see also S1–S3). The compounds are air-stable and did not require special handling conditions; no signs of degradation were observed after more than 1 year under ambient conditions. The isomers will be hereafter labeled as **M-I** (shorter retention time) and **M-II** (longer retention time). Absorption spectra of **M-I** and **M-II** are very similar to those of pristine fullerenes, which points to the formation of open adducts with minimal perturbation on the fullerene π-system (all carbons preserve their sp^2^ hybridization). Both MSc_2_N@C_80_ and their Ad adducts show almost featureless absorption spectra at wavelengths shorter than 650 nm and low-intensity broad bands at 650–800 nm (Figure 2c). Yet, the spectra demonstrate some sensitivity to isomeric structures, as **Nd-II** and **Dy-II** exhibit a sharper low-energy band than **Nd-I** and **Dy-I**.

Regio-isomerism of Ad addition to MSc_2_N@C_80_ is caused by the presence of two types of C–C bonds in the *I*_*h*_-C_80_ cage, denoted hereafter as [5,6] (pentagon/hexagon edge) and [6,6] (hexagon/hexagon edge) (Figure 1b). The earlier study of Ad addition to Sc_3_N@C_80_ and Lu_3_N@C_80_ reported the formation of [5,6]-open and [6,6]-open adducts with a relative yield ratio of 1:6.2 for Sc_3_N and 1:22 for Lu_3_N.^68^ In this work, Ad addition to MSc_2_N@C_80_ also preferably occurs across the [6,6] bond (**M-II** isomers), but the relative yields of 1:1.6 for **Nd-I**:**Nd-II** and 1:2.8 for **Dy-I**:**Dy-II** indicate that the nitride cluster composition has a strong influence on the distribution of isomeric monoadducts, with the yield of minor [5,6] isomers being enhanced for asymmetric MSc_2_N clusters. The only mixed-metal clusterfullerene studied in reaction with AdN_2_ before this work was Dy_2_TiC@C_80_, for which selective formation of the [6,6] isomer of Dy_2_TiC@C_80_(Ad) with exclusive coordination of the Ad addition site by Ti was found.^66^ As we demonstrate in the next section, the nitride cluster MSc_2_N shows a different coordination preference than carbido-Dy_2_TiC.

#### Paramagnetic ^1^H NMR Spectroscopy

Molecular structures of MSc_2_N@C_80_(Ad) adducts can be conveniently addressed by ^1^H NMR spectroscopy. Paramagnetism of metal ions poses complications in the form of strong paramagnetic shifts and broadening of the signals, but on the other hand, it gives new insights that diamagnetic analogs would not be able to provide, such as a strong sensitivity to metal position,^70,78,79^ akin to lanthanide shift reagents. Paramagnetic shift can also be a serious advantage, since it moves ^1^H signals of EMF derivatives outside of the range of 0–10 ppm, where they would otherwise overlap with signals from diamagnetic solvent residues, small-molecule residues from chromatographic columns used in the EMF separation, and the lock. Given the limited number of EMF derivatives, reliable detection of their signals in this range is severely complicated. We will therefore not consider the range of 0–10 ppm in the following discussion.

A standalone Ad moiety has 14 protons and *C*_2*v*_ symmetry with two mirror planes. In this symmetry, protons are grouped into five types (2*a*, 4*b*, 4*c*, 2*d*, and 2*e*), shown in Figure 3a. Upon attachment to the *I*_h_-C_80_ cage, the Ad symmetry is reduced to *C*_s_, whereby only one of the mirror planes is preserved. Since the remaining plane has a different orientation in [5,6] and [6,6] adducts, the isomers will have different numbers of inequivalent protons: nine ^1^H signals of (*a*, *a*’, 2*b*, 2*b*’, 2*c*, 2*c*’, 2*d*, *e*, *e*’) types are expected for the [5,6] adduct, whereas the [6,6] isomer should show eight signals of (2*a*, 2*b*, 2*b*’, 2*c*, 2*c*’, *d*, *d*’, 2*e*) types.

**Figure 3:**
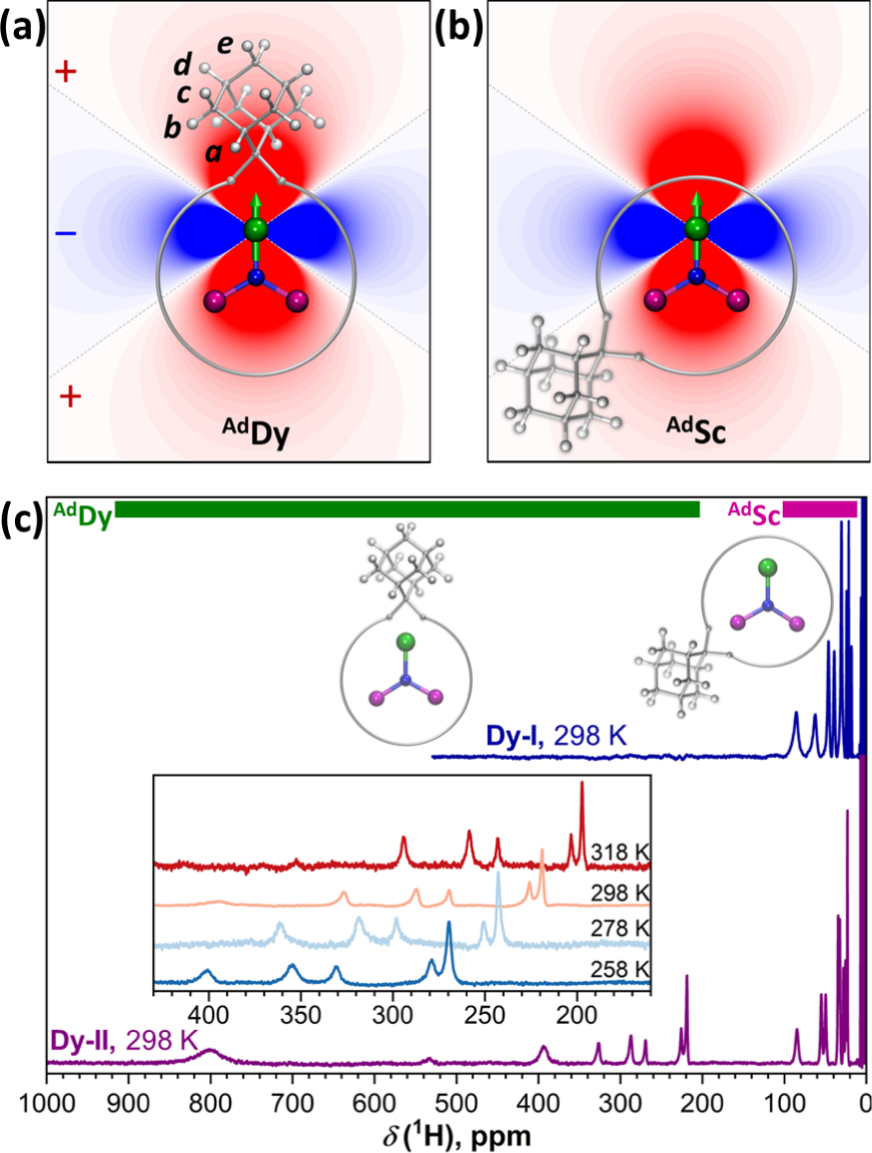
(a, b) Map of the geometrical factor (3cos^2^θ–1)/*R* ^3^ around Dy^3+^ ion (red – positive, blue – negative) overlaid with schematic molecular structures of DySc_2_N@C_80_(Ad) with (a) ^Ad^Dy and (b) ^Ad^Sc coordination; orientation of the Dy^3+^ quantization axis is shown with the green arrow. (c) ^1^H NMR spectra of **Dy-I** and **Dy-II** (*T* = 298 K, solvent CS_2_); chemical shift ranges of ^Ad^Dy and ^Ad^Sc forms are highlighted with green and magenta stripes, respectively. Inset shows variable-temperature spectra of **Dy-II** in part of the ^Ad^Dy range with the sharpest peaks.

Orientation of the MSc_2_N cluster with respect to the Ad addition site will also play a strong role in determining ^1^H chemical shifts. With partially filled 4f shell, both Nd^3+^ (4f^3^) and Dy^3+^ (4f^9^) have nonzero angular momentum. Under the influence of the nitride ion, they experience a strong uniaxial ligand field, aligning the magnetic moment along the M–N bond (*vide infra*). Dipolar interactions between the magnetic moment of the metal ion and a proton nuclear spin induce a pseudocontact chemical shift $\delta_{i}^{pc}$ , which for fast-rotating molecules in solution is described as a product of the geometrical term and the anisotropy of magnetic susceptibility *χ*:^80^

1 $$\delta_{i}^{pc}=\frac{3\cos^{2}\theta_{i}-1}{12\pi R_{i}^{3}}\left(\chi_{∥}-\chi_{⊥}\right)$$

where *R*_*i*_ is the distance between the metal atom and *i*th proton, *θ_i_* is the angle between the magnetic moment of the metal and the radius vector *R_i_*, and *χ*_∥_ (*χ*_⊥_) are the longitudinal (transversal) components of the magnetic susceptibility tensor. Here, we assume that the *χ* tensor is axial; otherwise, additional terms will appear in eq 1. Pseudocontact shifts thus contain information on both molecular geometry and magnetic anisotropy, making paramagnetic NMR a useful tool in the study of molecular magnets.^81–91^

The spatial distribution of the (3cos^2^θ – 1)/*R* ^3^ function is plotted in Figure 3a,b. Depending on how the MSc_2_N cluster is coordinated to the Ad-addition site, either by Sc (denoted hereafter as ^Ad^Sc) or by a lanthanide (^Ad^Dy or ^Ad^Nd coordination), a strong variation in pseudocontact shifts is expected. For instance, Dy···H distances in DFT-optimized structures of DySc_2_N@C_80_(Ad) span the range of 4.3–7.7 Å in the ^Ad^Dy form and 7–11 Å in the ^Ad^Sc form, resulting on average in a 10-fold larger geometrical term for ^Ad^Dy in comparison to ^Ad^Sc.

^1^H NMR spectra of **Dy-I** and **Dy-II** measured at 298 K in CS_2_ solution are compared in Figures 3c and 4a,b. Whereas for **Dy-I**, we found one set of nine signals in the range of 15–90 ppm (Figure 4a), **Dy-II** showed two sets of signals. Eight peaks are observed in the similar range of 20–90 ppm as for **Dy-I** (Figure 4b), while another set of eight peaks spans the much broader range of 200–800 ppm (Table S1). Measurements at different temperatures revealed considerable variation in chemical shifts between 258 and 318 K (Figures 4, S4 and S5) and ensured that the number of signals is not confused by an accidental overlap of peaks at room temperature. Therefore, we can conclude that **Dy-I** and **Dy-II** isomers are [5,6] and [6,6] adducts, respectively. Two sets of peaks in **Dy-II** suggest the presence of both ^Ad^Dy (200–800 ppm) and ^Ad^Sc (20–90 ppm) forms, as the ratio of the chemical shifts corresponds to the expected ratio of geometrical factors. Recycling HPLC did not show signs of the further separation of **Dy-II** and **Nd-II**. Either ^Ad^Dy and ^Ad^Sc forms have very similar retention times, or they can interconvert and equilibrate, which would preclude their separation. In the latter case, the rearrangement should be sufficiently slow on the NMR time scale to allow the observation of their individual spectroscopic features. Unfortunately, our attempts to detect the interconversion of the two forms by using 2D or 1D EXSY-NMR techniques (EXSY stands for EXchange SpectroscopY) were not conclusive because of the fast paramagnetic relaxation. By the similarity of the chemical shift ranges with **Dy-II**, we can conclude the dominance of the ^Ad^Sc coordination for **Dy-I** and exclude the fast interconversion between ^Ad^Dy and ^Ad^Sc forms for this isomer, as the latter would give ^1^H signals at intermediate positions. Besides, very weak signals in the range of the ^Ad^Dy form are detectable for **Dy-I** as well (Figure S4d).

**Figure 4:**
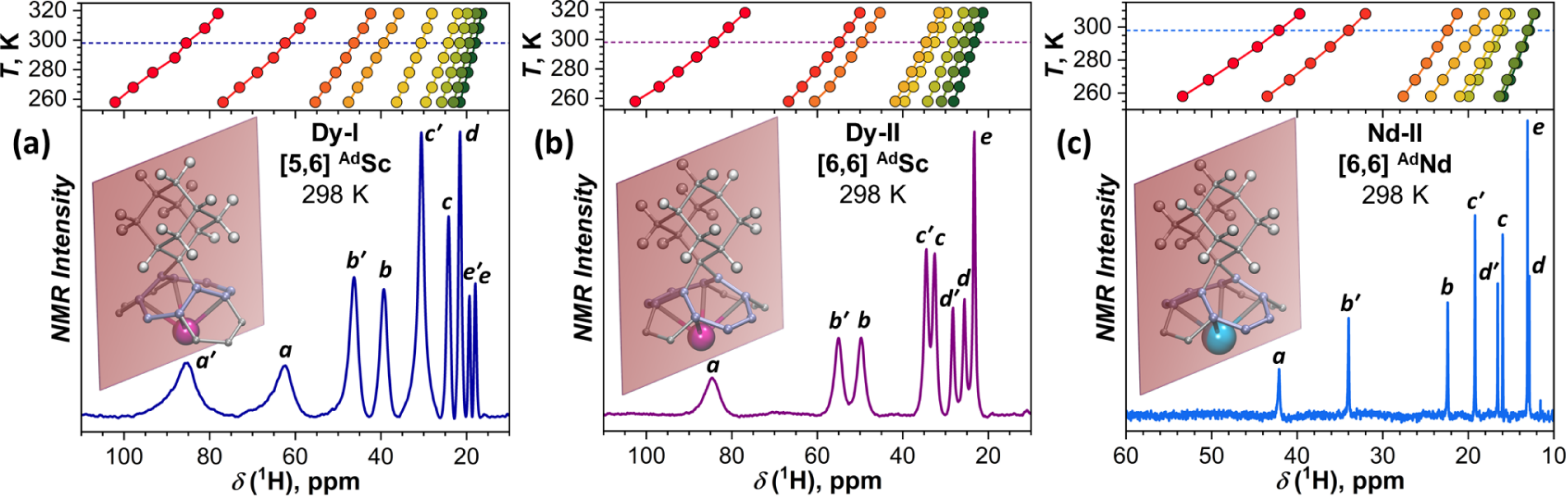
^1^H NMR spectra of (a) **Dy-I** and (b) **Dy-II** in the ^Ad^Sc range, and (c) **Nd-II** in the ^Ad^Nd range (*T* = 298 K, solvent CS_2_); upper panels describe variation of chemical shifts with temperature. Insets show orientation of the symmetry plane crossing the Ad moiety, giving 9 and 8 unique protons for [5,6] and [6,6] isomers, respectively. Letters at NMR peaks denote their tentative assignment to different types of Ad protons introduced in Figure 3a.

Our earlier ^13^C and ^45^Sc NMR study of the MSc_2_N@C_80_ series, which included M = Dy and Nd among other rare-earth metals, showed that the ^45^Sc paramagnetic shift in NdSc_2_N@C_80_ (161 ppm) is smaller than in DySc_2_N@C_80_ (1692 ppm) by an order of magnitude.^89^ Since geometric factors for these molecules are nearly identical, this difference was ascribed to the 10-fold smaller magnetic susceptibility term of Nd. Assuming analogous variation of susceptibility for Nd and Dy in MSc_2_N@C_80_(Ad) and similar values of geometrical factors, 10-fold smaller ^1^H chemical shifts can be expected for **Nd-I** and **Nd-II** in comparison to **Dy-I** and **Dy-II**. This places the chemical shifts of ^Ad^Sc forms of NdSc_2_N@C_80_(Ad) into the range less than 10 ppm, where reliable detection of their signals is complicated. At the same time, signals of the ^Ad^Nd form are expected at more positive shifts, where they can be firmly detected. Indeed, **Nd-II** showed one set of 8 peaks at 12–44 ppm (Figure 4c), which we assign to the ^Ad^Nd form of the [6,6] isomer, while **Nd-I** did not show ^1^H signals above 10 ppm. Thus, similarly to Dy analogs, the [5,6] isomer of NdSc_2_N@C_80_(Ad) exists predominantly in the ^Ad^Sc form, while the presence of both ^Ad^Nd and ^Ad^Sc forms can be assumed for the [6,6] isomer. Based on the ratio between chemical shifts of ^Ad^M forms of **Dy-II** and **Nd-II**, we can estimate that the room-temperature susceptibility term of Nd^3+^ in the ^Ad^M configuration is ∼20 times smaller than that for Dy^3+^ (Table S1). This ratio is close to 24, as tabulated by Bleaney et al. for the ratio of chemical shifts in Dy and Nd complexes.^92^ However, analysis of the variable-temperature chemical shifts in DySc_2_N@C_80_(Ad) and NdSc_2_N@C_80_(Ad) in terms of the *T* ^–2^ dependence suggested by Bleaney’s theory for pseudocontact shifts^92,93^ revealed significant deviations from the theory (Figure S5). This is not surprising, given that Bleaney’s assumption on the ligand field splitting being less than thermal energy is not fulfilled.

#### DFT Calculations

To obtain systematic information on molecular structures and relative stability of different orientations of the endohedral cluster in MSc_2_N@C_80_(Ad) adducts, DFT calculations were performed using Y as a model, as it has similar ionic radius to Dy. For both [5,6] and [6,6] isomers of YSc_2_N@C_80_(Ad), we used Fibonacci sampling^94^ to prepare 120 starting configurations with different orientations of the YSc_2_N cluster. Additional configurations were also obtained by rotating the endohedral cluster around the^Ad^Sc–N or ^Ad^Y–N bond with a step of 15°. The structures were then optimized at the PBE/TZ2P level using the fast DFT code Priroda.^95,96^

Optimized molecular structures of the most stable conformers are shown in Figure 5, while more complete data are presented in Figures S6 and S7 and Table S2. For the bare YSc_2_N@C_80_, an analogous survey located 5 unique conformers,^97^ all within 4 kJ mol^–1^. For YSc_2_N@C_80_(Ad) adducts, the calculations revealed four types of structures, which are sorted in Figure 5a,b according to their relative energy (Δ*E*) and the C–C distance in the Ad-addition site. The conformers, in which endohedral metal atoms do not coordinate to the addition site span relative energies of 45–75 kJ mol^–1^. They are roughly equally distributed between “closed” adducts with C–C distances of less than 1.8 Å and “open” adducts with C–C distances longer than 1.9 Å. The structures with metal-coordinated Ad sites have longer C–C distances (>2.1 Å) and are considerably more stable (Δ*E* = 0–20 kJ mol^–1^), in agreement with the observation of metal-coordinated Ad sites in the majority of adducts of different EMFs.^62,66–68,70–72,98^ Note that rare examples of noncoordinated Ad pockets in EMF­(Ad) derivatives were also reported.^99,100^

**Figure 5:**
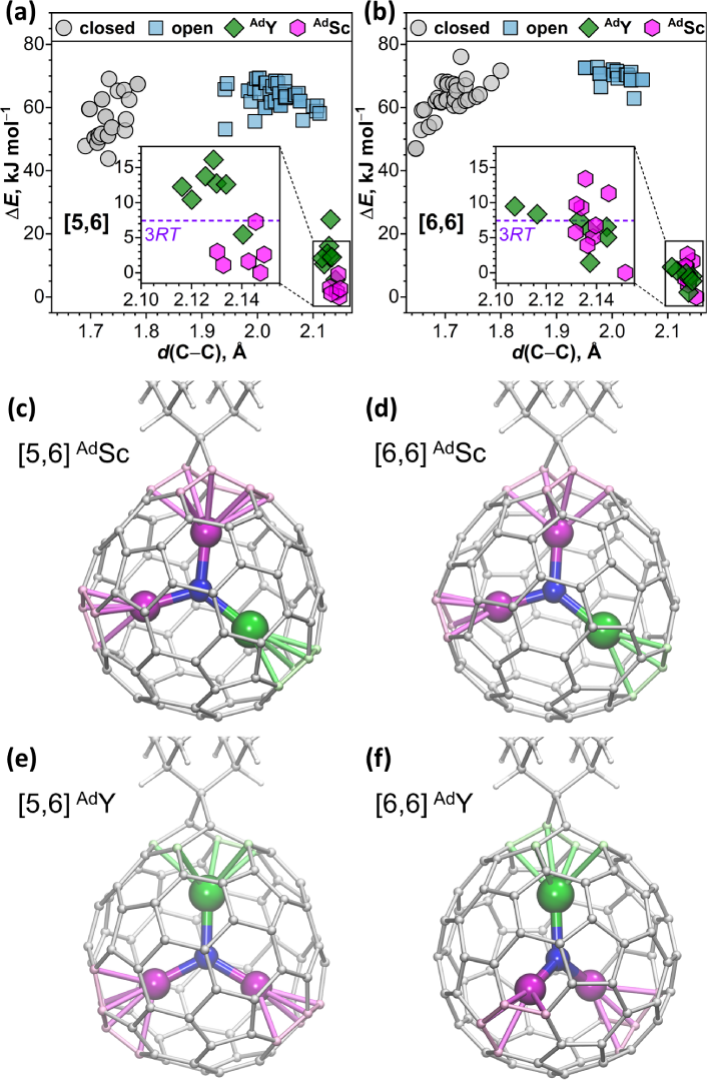
(a, b) DFT (PBE/TZ2P) relative energies of conformers with different orientations of the YSc_2_N cluster in [5,6] (a) and [6,6] (b) isomers of YSc_2_N@C_80_(Ad), sorted according to C–C distance in the Ad addition site; ^Ad^Y and ^Ad^Sc are conformers in which the Ad addition site is coordinated by Y and Sc atoms, respectively. To guide the eye, dashed lines in the insets are plotted at Δ*E* = 7.4 kJ mol^–1^, corresponding to 3RT at 298 K. (c–f) Optimized molecular structures of the most stable [5,6] and [6,6] isomers of YSc_2_N@_C80(_Ad) with ^Ad^Sc (c, d) and ^Ad^Y (e, f) configurations. Y – green, Sc – magenta, N – blue; Y–C and Sc–C distances shorter than 2.6 and 2.5 Å, respectively, are visualized as bonds.

^Ad^Sc conformers tend to be more stable than their ^Ad^Y counterparts, but the patterns for [5,6] and [6,6] isomers are different. For the [5,6] adduct, the lowest-energy ^Ad^Sc conformer is more stable than ^Ad^Y by 5 kJ mol^–1^. Furthermore, ^Ad^Sc conformers are grouped in the range of 0–7 kJ mol^–1^, whereas all but one ^Ad^Y conformer are located above 9 kJ mol^–1^ (Figure 5a). For the [6,6] adduct, the energy difference between the most stable ^Ad^Y and ^Ad^Sc conformers is only 1.4 kJ mol^–1^, while the other ^Ad^Sc and ^Ad^Y conformers are clustered in the same energy range (Figure 5b). These results suggest that, in the thermodynamic equilibrium, one can expect the dominance of ^Ad^Sc conformers for the [5,6] isomer and a comparable concentration of ^Ad^Y and ^Ad^Sc conformers for the [6,6] isomer. This perfectly agrees with our NMR results, in which ^Ad^Sc configurations were observed for both [5,6] and [6,6] isomers, while the appreciable amount of ^Ad^Dy and ^Ad^Nd configurations was only found for the [6,6] isomer.

A possibility of interconversion between conformers determines how they will appear in NMR spectra. Transformation within the set of ^Ad^M (^Ad^Sc) conformers proceeds via rotation around the ^Ad^M–N (^Ad^Sc–N) bond. As all such conformers are grouped at small relative energies, and considering that the barriers for the cluster rotation in M_3_N@*I*_h_-C_80_ are usually well below 20 kJ mol^–1^,^101–104^ similarly low barriers can be expected for the rotation around ^Ad^M–N or ^Ad^Sc–N bonds in MSc_2_N@C_80_(Ad). Computational and single-crystal X-ray diffraction studies of [6,6]-open M_3_N@*I*_h_-C_80_ adducts also demonstrate facile rotation of the M_3_N cluster around the ^Ad^M–N bond even at reduced temperatures used in diffraction measurements.^62,64,66,68,98,105^ Analogous rotation of the MSc_2_N cluster in MSc_2_N@C_80_(Ad) should also be fast on the NMR time scale near room temperature, resulting in averaging over conformers and ensuring experimentally observed effective *C*_s_ symmetry.

Different dynamics are expected for the transformation between ^Ad^M and ^Ad^Sc forms. Here, the metal should inevitably escape the Ad-pocket, likely proceeding via one or more uncoordinated conformers at intermediate steps. Unfortunately, we did not succeed in locating a transition state for this transformation, but sheer relative energies of uncoordinated conformers (Figure 5a,b) indicate that the interconversion barriers should be substantial. We therefore suggest that ^Ad^Dy and ^Ad^Sc forms are either frozen in the ratio that occurred during the Ad addition or can equilibrate at room temperature. The equilibration should then be sufficiently slow on the NMR time scale to allow observation of distinct NMR signals for the two types of conformers.

### Magnetic Anisotropy of Nd^3+^ and Dy^3+^ in MSc_2_N@C_80_(Ad)

With the structural peculiarities of MSc_2_N@C_80_(Ad) adducts having been clarified, we can focus on the analysis of the magnetic anisotropy of lanthanide ions in these compounds, the influence of the chemical derivatization thereupon, and its consequences for the magnetization dynamics. At first, we analyze the ligand-field splitting in NdSc_2_N@C_80_(Ad) by lanthanide-based photoluminescence (Ln-PL), then rationalize these results by *ab initio* CASSCF/RASSI-SO calculations of single-ion magnetic properties, and finally proceed to study the SMM behavior in DySc_2_N@C_80_(Ad) by SQUID magnetometry.

#### Nd-Based Photoluminescence

The N^3–^ ion in nitride clusterfullerenes is located at a short distance to a lanthanide and induces a strong ligand field (LF) for the latter. The size of the LF splitting and the structure of the LF levels in Dy-EMFs have been extensively studied computationally (*vide infra*) in lieu of their single-molecule magnetism, but the experimental spectroscopic analysis has not been successful yet. For instance, Dy-PL, which has been quite useful for other molecular complexes of Dy,^106–117^ cannot be observed for Dy-EMFs because of the low energy of fullerene excited states (∼1.6 eV, Figure 2c) in comparison to the emitting level of Dy^3+^ (^4^F_9/2_ at ∼2.6 eV). Near-infrared Ln-PL is more accessible for experimental exploration in EMFs,^49,118–123^ and we recently succeeded in the detection of the finely structured Nd-PL of NdM_2_N@C_80_ (M = Sc, Y, Lu).^31^

Figures 6 and S8 show variable-temperature PL spectra of NdSc_2_N@C_80_(Ad) isomers in the energy range of the Nd-PL. Both **Nd-I** and **Nd-II** showed Nd-based NIR emission, which is weak and poorly structured at room temperature but gains intensity and resolution upon cooling. The highest-energy PL band at 900–1020 nm (^4^F_3/2_→^4^I_9/2_) is visibly stronger than^4^F_3/2_→^4^I_11/2_ at 1100–1250 nm, while ^4^F_3/2_→^4^I_13/2_ at ∼1400 nm is barely seen. This is different from the vast majority of Nd^3+^ compounds, which typically exhibit the highest-intensity ^4^F_3/2_→^4^I_11/2_ band, but is similar to the intensity distribution in NdSc_2_N@C_80_. Remarkably, the PL bands of NdSc_2_N@C_80_ and NdSc_2_N@C_80_(Ad) are red-shifted from their common positions in Nd^3+^ compounds, which we attribute to the very strong nephelauxetic effect caused by the short Nd–N bond length and enhanced covalency of the bonding.^31^

**Figure 6:**
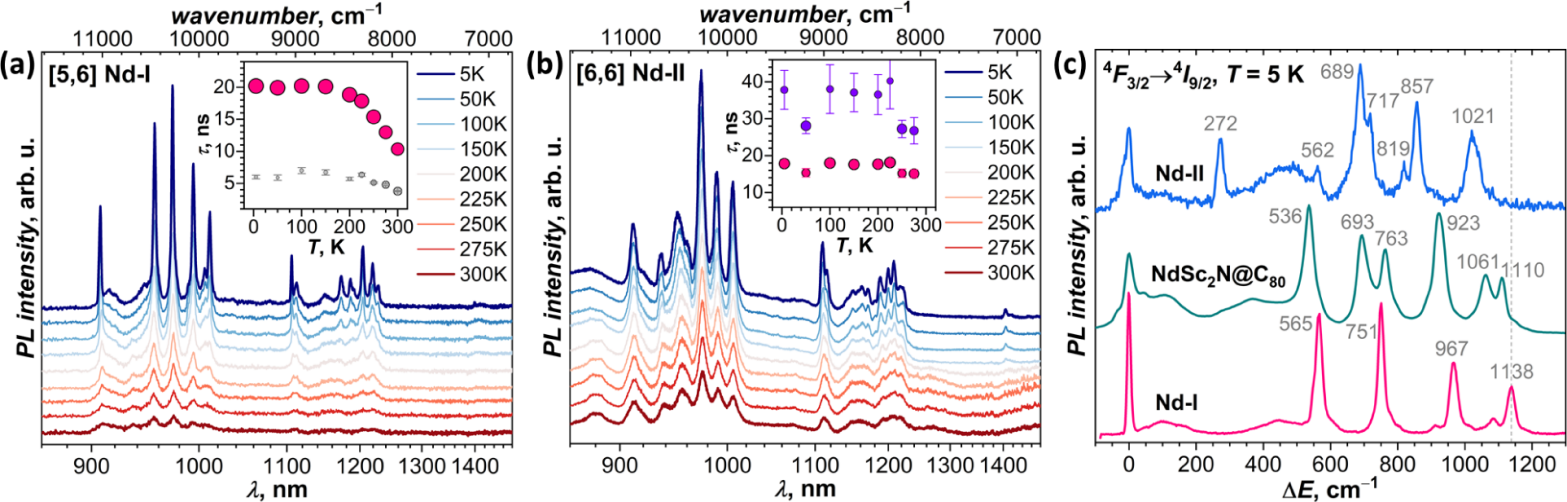
Variable-temperature photoluminescence spectra of (a) [5,6] and (b) [6,6] isomers of NdSc_2_N@C_80_(Ad) in the energy range of Nd^3+^ f-f transitions (^4^F_3/2_→^4^I_*J*_, *J* = 9/2, 11/2, 13/2); insets show temperature variation of PL lifetimes, determined by biexponential fit of PL decays. (c) Low-temperature fine structure of the ^4^F_3/2_→^4^I_9/2_ band in **Nd-I**, **Nd-II**, and NdSc_2_N@C_80_ caused by the ligand-field splitting in the ground state multiplet ^4^I_9/2_.

PL decay measurements showed that the lifetimes of **Nd-I** and **Nd-II** are much shorter than the 0.2–2 μs usually observed for Nd complexes. Decay curves of both compounds can be fitted with two lifetimes (Figures S9–S11). Below 200 K, **Nd-I** has a main lifetime of 20 ns with a small contribution from a shorter (∼6 ns) component (Figure 6a). **Nd-II** shows two components with comparable weights (Figure 6b): one with a lifetime of 18 ns (∼70%) and the second with a longer lifetime of 30–40 ns (30%). The PL of nonderivatized NdSc_2_N@C_80_ was described by a biexponential decay with lifetimes of 19 ns (main) and 4 ns (minor).^31^ A plausible interpretation is that the Nd-PL lifetime depends on how the Nd^3+^ ion is coordinated to the fullerene cage. Coordination to the nonfunctionalized cage fragment, as in the pristine NdSc_2_N@C_80_ or in ^Ad^Sc forms of NdSc_2_N@C_80_(Ad), gives the lifetime near 18–20 ns, whereas the ^Ad^Nd form has a lifetime of 30–40 ns.

The fine structure of the ^4^F_3/2_→^4^I_9/2_ band is caused by the ligand-field splitting of the ground-state multiplet ^4^I_9/2_. By the number of Kramers doublets (KDs) in this multiplet, five PL transitions can be expected at helium temperature, when hot bands are frozen out. We indeed observed five sharp peaks at Δ*E* = 0, 565, 751, 967, and 1138 cm^–1^ in the PL spectrum of **Nd-I** measured at 5 K (Figures 6c and 7a). Two small features at 914 and 1085 cm^–1^ can be assigned to a minor conformer with a somewhat different Nd-cage coordination geometry. For comparison, the Nd-PL spectrum of NdSc_2_N@C_80_ revealed a slightly smaller LF splitting of 1110 cm^–1^ and the presence of at least two conformers with comparable contributions (Figure 6c).

**Figure 7:**
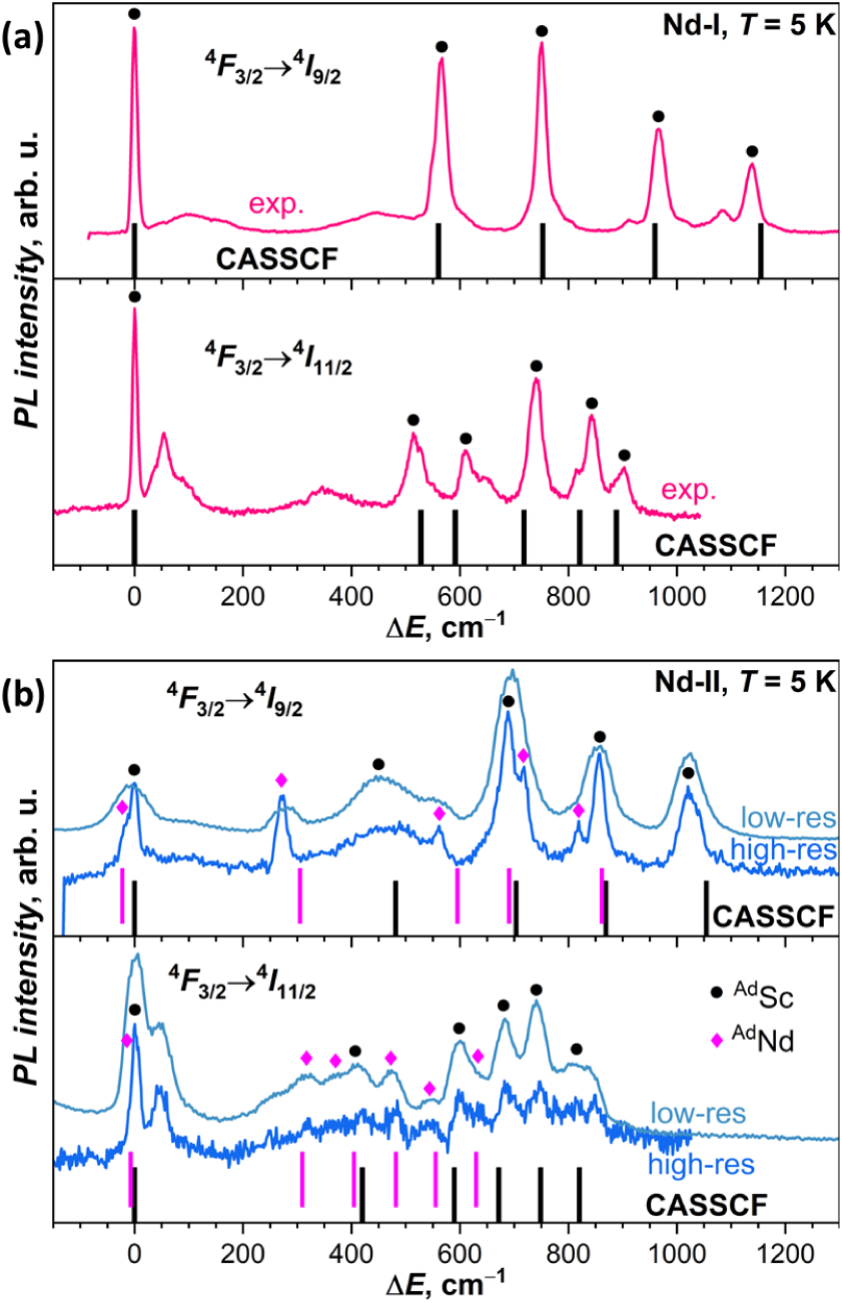
(a) Low-temperature PL spectra of **Nd-I** in the ranges of ^4^F_3/2_→^4^I_9/2_ and ^4^F_3/2_→^4^I_11/2_ bands; experimental spectra are compared to the results of CASSCF/RASSI-SO calculations for the lowest-energy ^Ad^Sc conformer (vertical bars; computed energies are multiplied by 1.19 to account for the lack of dynamic correlation^74^). (b) Low-temperature PL spectra of **Nd-II** in the ranges of ^4^F_3/2_→^4^I_9/2_ and ^4^F_3/2_→^4^I_11/2_ bands; experimental spectra are compared to the results of CASSCF/RASSI-SO calculations for the lowest-energy ^Ad^Sc and ^Ad^Nd conformers (black and magenta bars, respectively; computed energies are multiplied by 1.19). Since signal-to-noise ratio in the high-resolution spectrum of the ^4^F_3/2_→^4^I_11/2_ band of **Nd-II** is too low, the spectra measured with lower resolution are also shown (see also Figure S7). Tentative assignment of PL peaks to individual KDs of ^Ad^Sc and ^Ad^Nd forms is given by black circles and magenta diamonds, respectively. Δ*E* = 0 cm^–1^ corresponds to 906.9 nm (^4^F_3/2_→^4^I_9/2_, **Nd-I**), 1105.8 nm (^4^F_3/2_→^4^I_11/2_, **Nd-I**), 910.8 nm (^4^F_3/2_→^4^I_9/2_, **Nd-II**), and 1107.8 nm (^4^F_3/2_→^4^I_11/2_, **Nd-II**).

**Nd-II** shows a more complex Nd-PL spectrum. The ^4^F_3/2_→^4^I_9/2_ band has more than five peaks, which can be ascribed to the presence of ^Ad^Sc and ^Ad^Nd forms suggested by NMR spectroscopy. Whereas the gap between the lowest-energy and the first excited KD (Δ_1,2_) is more than 500 cm^–1^ in **Nd-I** and NdSc_2_N@C_80_, **Nd-II** has the peak at 272 cm^–1^. Since there is no reason for ^Ad^Sc and ^Ad^Nd forms to emit at exactly the same energy, it is not possible to unambiguously extract the information on the LF splitting in each of them (see Figure 7b for a tentative assignment), but it can be concluded that the Δ_1,2_ energy in one of the forms is definitively smaller than in **Nd-I** and NdSc_2_N@C_80_. The upper limit of the LF splitting in ^Ad^Sc and ^Ad^Nd forms of **Nd-II** is given by the total span of the ^4^F_3/2_→^4^I_9/2_ band, equal to 1021 cm^–1^, which is noticeably smaller than the LF splitting of 1138 cm^–1^ determined for the ^Ad^Sc form of **Nd-I**.

#### Computational Studies

The LF imposed on lanthanide ions in nitride clusterfullerenes of the MSc_2_N@C_80_ type and their derivatives has two main sources: (i) the nitride ion and (ii) the bonding with the fullerene cage. The former has a much stronger and predominantly axial contribution, but the M–N^3–^ interaction hardly changes between [5,6] and [6,6] isomers and their conformers. On the other hand, although the metal-cage interaction is weaker, it yields a nonaxial contribution to the ligand field, which is very sensitive to the metal-cage coordination geometry. The variation of the single-ion anisotropy between the isomers and conformers is therefore mainly caused by metal-cage interactions, which we address in this section with QTAIM and CASSCF calculations.

Figure 8a shows a metal-coordinated fragment of the nonfunctionalized fullerene cage along with the QTAIM atomic charges, using the lowest-energy [5,6]-^Ad^Sc conformer as a representative example and Y as a model for a lanthanide. In nitride clusterfullerenes, each metal atom formally transfers one electron to the nitrogen and two electrons to the fullerene. The QTAIM charge of the nitride ion is −1.85, while the charge transferred from Y to the fullerene is −1.40, mainly distributed over the island of 12 carbon atoms. Atomic charges of individual carbons do not exceed −0.12 and gradually decrease with the distance from the metal ion. Y–C delocalization indices (DI, QTAIM analogs of bond orders) follow a similar pattern as atomic charges. The carbon closest to Y has a DI of 0.24; the values for other atoms are smaller and decrease with the Y–C distance, while the total Y-cage “bond order” is 1.65. Overall, this is a typical example of metal-cage bonding described for many types of EMFs with rare-earth metals.^124^ Its semilocal character results in a high degree of flexibility in the sense that there is no universally predefined metal-cage coordination geometry. Lateral displacement of the metal atom along the fullerene inner surface decreases interactions with some carbons but increases interactions with others, creating a possibility for multiple conformers with similar energies in isotropic fullerene cages, particularly in *I*_h_-C_80_ (Figure 5). Although these conformers are not very different energetically, the variation in metal-cage coordination can lead to pronounced changes in the single-ion magnetic anisotropy.

**Figure 8:**
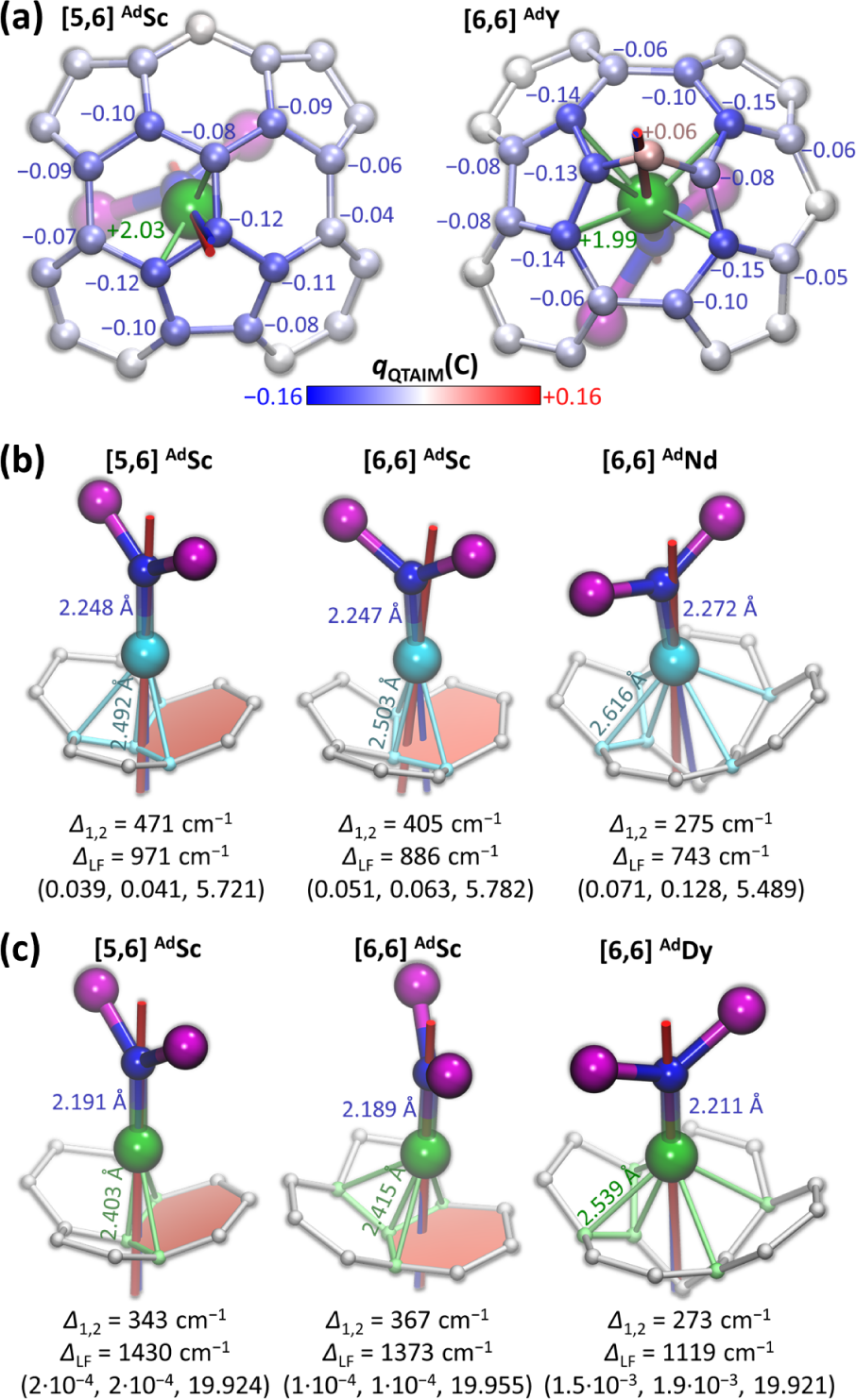
(a) QTAIM charges of carbon atoms in metal-coordinated fragments of the cage for [5,6] ^Ad^Sc and [6,6] ^Ad^Y isomers of YSc_2_N@C_80_(Ad). (b, c) Lanthanide ions with fragments of the fullerene cage for representative conformers of (b) NdSc_2_N@C_80_(Ad) and (c) DySc_2_N@C_80_(Ad). Shown for each structure are the orientation of the quantization axis for the ground-state Kramers doublet (red line) and geometrical M–N axis (blue line), as well as M–N and the shortest M–C bond lengths (M = Nd, Dy). Nd–C and Dy–C distances shorter than 2.65 and 2.60 Å, respectively, are visualized as bonds. Also listed in (b, c) are the gap between the first and the second Kramers doublets (Δ_1,2_), full ligand field splitting of the ground-state multiplet (Δ_LF_), and principal values of the pseudospin g-tensor of the ground-state Kramers doublet. Structure optimization and computation of electron density for QTAIM analysis are performed with PBE0 functional using the Orca suite;^126^ ligand-field splitting and g-tensors are computed at the CASSCF/RASSI-SO level.

For the *ab initio* calculations of LF splitting, conformers of YSc_2_N@C_80_(Ad) were reoptimized for the corresponding Nd and Dy structures at the PBE-D/PAW level with 4f-shell in-core treatment using the VASP code;^125^ Table S2 demonstrates that the results are not much different from the YSc_2_N@C_80_(Ad) data. Figure 8b,c shows Nd-cage and Dy-cage coordination fragments in the most stable conformers of [5,6]-^Ad^Sc, [6,6]-^Ad^Sc, and [6,6]-^Ad^M along with the orientation of the *g*_z_ principal axis of the pseudospin *g*-tensor for the ground-state Kramers doublet (KD1), principal values of the *g*-tensor, and the size of the LF splitting (Δ_LF_), all computed at the CASSCF/RASSI-SO level (see Tables S3–S6 for numerical data). Whereas the strong interaction with the nitride ion aligns the quantization axis along the M–N bond, metal–carbon interactions deflect the axis from the strict M–N direction. The N–M–C angle can be used as a measure of the nonaxiality for a given carbon position, while the angle between the M–N bond and the *g*_z_ axis can serve as an indicator of how the cage perturbs the nitride-induced axiality.

In the [5,6]-^Ad^Sc conformer of NdSc_2_N@C_80_(Ad) shown in Figure 8b, the closest carbon is at an Nd–C distance of 2.492 Å and with a N–Nd–C angle of 174.5°, is located in an almost perfect head-on position. Less axial positions of carbons next to it, with Nd–C distances/N–Nd–C angles of 2.553 Å/148.7°, 2.563 Å/148.0 Å, and 2.636 Å/141.5°, are counterbalanced by their nearly symmetric triangular arrangement and longer Nd–C distances. As a result, this coordination geometry provides the strongest support for the magnetic axiality among all conformers of NdSc_2_N@C_80_(Ad) that we studied. The Nd–N/*g*_*z*_ angle is only 2.7°, and the LF splitting (Δ_LF_) of 970 cm^–1^ is the largest among all of the conformers. After single-factor correction for the systematic underestimation of CASSCF-computed LF splitting in lanthanide EMFs by 15–20%,^74^ theoretical KD energies in ^4^I_9/2_ and ^4^I_11/2_ multiplets agree very well with the fine structure of the ^4^F_3/2_→^4^I_9/2_ and ^4^F_3/2_→^4^I_11/2_ PL bands of **Nd-I** (Figure 7a).

In the lowest-energy [6,6]-^Ad^Sc conformer of NdSc_2_N@C_80_(Ad) (Figure 8b), Nd is located in the off-center position above the hexagon. With the N–Nd–C angle of 161.5°, the closest carbon at a distance of 2.503 Å is clearly less axial than in [5,6]-^Ad^Sc, resulting in an increase of the Nd–N/*g*_*z*_ deflection angle to 9.2° and a decrease in the LF splitting by ∼10%. The reduced Δ_LF_ of 886 cm^–1^ agrees well with the smaller LF splitting in the experimental PL spectrum of **Nd-II** (Figure 7b). Overall, the findings about ^Ad^Sc conformers of NdSc_2_N@C_80_(Ad) follow the trend for Nd-cage coordination revealed in our recent study of NdSc_2_N@C_80_: the more central position above the hexagon that Nd occupies, the lower the axiality and overall LF splitting.^31^ Depending on the coordination geometry, the Δ_LF_ value can vary by up to 25%, ranging from 780 to 970 cm^–1^, while the Nd–N/*g*_*z*_ deflection angle can reach 13°. Our attempts to find more distinct correlations between metal-cage coordination geometry and ligand-field splitting were not successful (Figures S12–S18).

Low-energy ^Ad^Sc conformers of [5,6] and [6,6] isomers of DySc_2_N@C_80_(Ad) as shown in Figure 8c have metal-cage coordination similar to that of their Nd counterparts, except for somewhat shorter Dy–C and Dy–N bonds. However, the influence of Dy-cage interactions on the orientation of the quantization axis is much weaker, with the Dy–N/*g*_*z*_ deflection angle being less than 4° for all studied ^Ad^Sc structures. The variability of the LF splitting among ^Ad^Sc conformers, 1370–1440 cm^–1^, is also less pronounced than in the Nd series.

This very different sensitivity of the magnetic anisotropy of Nd^3+^ and Dy^3+^ to the fullerene cage stems from Stevens coefficients *θ*_*k*_, which enter the crystal field parameters as $B_{q}^{k}=A_{q}^{k}\left\langle r^{k}\right\rangle \theta_{k}$ (*k* = 2, 4, 6;*−k* ≤ *q* ≤ *k*), where $A_{q}^{k}$ reflects the coordination geometry and, in the first approximation, is metal-independent. The product of the radial integral and Stevens coefficient, ⟨*r^k^* ⟩*θ_k_*, is the intrinsic property of the metal ion transferable between different environments. Nd^3+^ and Dy^3+^ have similar *θ*_2_ values, but *θ*_4_ and *θ*_6_ coefficients of Nd^3+^ are larger than those of Dy^3+^ by factors of 5 and −37, respectively (Table 1). Considering that rank *k* coefficients are multiplied by $J_{\alpha }^{q}$ terms (*α* = *x*, *y*, *z*; *q* ≤ *k*), and that the total momentum of Nd^3+^ is smaller than that of Dy^3+^, relative weights of different ranks are better represented by the ratio *θ_k_J^k^*/*θ*_2_ *J* ^2^. As listed in Table 1, *θ*_4_ *J* ^4^/*θ*_2_ *J* ^2^ and *θ*_6_ *J* ^6^/*θ*_2_ *J* ^2^ ratios for Dy^3+^ are both near 0.5, but for Nd^3+^ they amount to 0.9 and 2.4, respectively (Table 1). The ratio of radial integrals for Nd^3+^ and Dy^3+^ will also favor higher ranks for Nd^3+^.^127^ Therefore, while the LF splitting in ^Ad^Sc conformers of DySc_2_N@C_80_(Ad) is dominated by 78–81% of $B_{q}^{2}$ terms with only 10–12% of $B_{q}^{6}$ , the contributions of $B_{q}^{2}$ and $B_{q}^{6}$ terms in ^Ad^Sc-NdSc_2_N@C_80_(Ad) redistribute to nearly equal shares of 42–45% and 40–42%, respectively. Likewise, the net weight of axial LF terms $B_{0}^{2}$ , $B_{0}^{4}$ , and $B_{0}^{6}$ is about 50–60% for Dy and is twice smaller, 23–33%, for Nd. The axiality of LF strongly affects the wave function composition of the states. In the *m_J_* basis, the ground-state KD1 of DySc_2_N@C_80_(Ad) is 99–100% |±15/2⟩, and the next three to four KDs also have a single dominant *m_J_* component (>85%), whereas considerable *m_J_* mixing starts from KD5 or KD6. In Nd counterparts, only the first KD is described as 85–91% of |±9/2⟩, while all higher-energy states are strongly mixed. Finally, pseudospin *g*-tensor of KD1 in ^Ad^Sc-DySc_2_N@C_80_(Ad) has very small *g_x_*_,_ *_y_* values of less than 3 × 10^–4^ and *g_z_* of 19.92–19.98, close to 2*Jg_J_* = 20 for Dy^3+^, whereas *g_x_*_,_ *_y_* in ^Ad^Sc-NdSc_2_N@C_80_(Ad) is found within the range of 0.02–0.09, while *g_z_* spans 5.7–6.0 and is noticeably smaller than 2*Jg_J_* = 6.54 for Nd^3+^. In brief, much larger *θ*_6_ coefficient and the ensuing enhanced weight of $B_{q}^{6}$ terms make Nd^3+^ substantially more susceptible to the nonaxial contribution to the LF imposed by the fullerene cage.

**Table 1:** Ligand-Field Splitting, Contribution of LF Parameters, and Selected Single-Ion Magnetic Parameters in ^Ad^Sc and ^Ad^M Conformers of [5,6] and [6,6] Isomers of MSc_2_N@C_80_(Ad) (M = Nd or Dy) According to CASSCF/RASSI-SO Calculations^a^

	M = Nd		M = Dy	
	^Ad^Sc	^Ad^Nd	^Ad^Sc	^Ad^Dy
*J*, *M* ^3+^multiplet	9/2, ^4^I_9/2_		15/2, ^6^H_15/2_	
*g_J_*	8/11		4/3	
θ_2_ × 10^–2^	–0.643		–0.635	
θ_4_ × 10^–4^; θ_4_ *J* ^4^/θ_2_ *J* ^2^	–2.911; 0.92		–0.592; 0.52	
θ_6_ × 10^–6^; θ_6_ *J* ^6^/θ_2_ *J* ^2^	–37.99; 2.42		1.035; – 0.52	
**LF splitting**				
Δ_1,2_, cm^–1^	250–500	240–330	340–460	260–320
Δ_LF_, cm^–1^	780–970	710–800	1370–1440	1080–1330
**LF parameters**				
$\underset{\left|q\right|\leq 2}{\sum }B_{q}^{2}$ in Δ_LF_, %	42–47	37–42	78–81	78–81
$\underset{\left|q\right|\leq 4}{\sum }B_{q}^{4}$ in Δ_LF_, %	10–13	8–11	7–8	5–6
$\underset{\left|q\right|\leq 6}{\sum }B_{q}^{6}$ in Δ_LF_, %	38–41	44–48	10–12	12–14
$\underset{\left|q\right|\leq 8}{\sum }B_{q}^{8}$ in Δ_LF_, %	3.3–4.7	3.2–6.4	2.0–2.5	1.7–2.3
**Axial LF parameters**				
$B_{0}^{2}$ , cm^–1^	–(9.1–10.5)	–(7.2–8.0)	–(7.9–8.1)	–(6.3–6.6)
$B_{0}^{2}$ in Δ_LF_, %	15–20	12–19	45–50	40–47
$\left(B_{0}^{2}+B_{0}^{4}+B_{0}^{6}\right)$ in Δ_LF_, %	24–33	17–37	49–58	43–51
**Properties of KD1**				
dominant |*m_J_* ⟩ in KD1, %	85–91	78–85	98.0–100.0	96.5–99.4
*g_x_*_,_ *_y_* for KD1	0.01–0.09	0.01–0.14	<3 × 10^–4^	<4 × 10^–3^
*g_z_* for KD1	5.7–6.0	5.4–5.7	19.92–19.98	19.78–19.94
**Magnetic susceptibility** **at 298 K**				
*χ*_*z*_, cm^3^ mol^–1^	9.69 × 10^–3^	8.47 × 10^–3^	1.18 × 10^–1^	1.11 × 10^–1^
(*χ*_*x*_ + *χ_y_*)/2, cm^3^ mol^–1^	2.27 × 10^–3^	2.87 × 10^–3^	1.06 × 10^–2^	1.44 × 10^–2^
${\left(\chi_{∥}-\chi_{⊥}\right)}_{\mathrm{Dy}}/{\left(\chi_{∥}-\chi_{⊥}\right)}_{\mathrm{Nd}}$			14.5	17.3
**SMM properties**				
*T*_B_/*T*_irrev_, K			7.9–9.0/10–11	4.6

a cgs system.

Coordination of the lanthanide ion to the Ad addition site gives a distinctly different metal-cage bonding pattern. According to QTAIM charges, Ad moiety transfers 0.26 *e* to the fullerene, which results in somewhat higher negative charges on the carbons next to the metal than in ^Ad^Sc conformers (Figure 8a). At the same time, the shortest metal–carbon distances in the ^Ad^Y configuration are longer by ∼0.1 Å than in ^Ad^Sc, and Y–C DIs are noticeably lower (the largest DI is 0.16 in the ^Ad^Y conformer versus 0.24 in ^Ad^Sc). By the nature of ^Ad^M coordination to the “open” C–C bond, the metal ion has no cage atoms in the head-on (axial) position. The closest in that direction is the carbon atom from the Ad moiety at a distance of ∼3.5 Å with a slightly positive charge (+0.06, Figure 8a). Nearest to the axial position are bridgehead atoms at ∼2.6 Å with N–Y–C angles of 152–160°. Carbons next to them on both sides of the open bond are slightly closer to the metal, bear the most negative atomic charges of −0.14 to −0.15, but have much smaller N–M–C angles of ∼130°, which places them closer to equatorial (90°) than axial (180°) positions. The Y–N distance in ^Ad^Y is also slightly longer than that in ^Ad^Sc conformers.

The utterly nonaxial metal-cage coordination geometry in the ^Ad^M configuration has a visible influence on the lanthanide magnetic anisotropy, as summarized in Table 1. The most pronounced changes, in comparison to ^Ad^Sc conformers, are considerably reduced LF splitting (Δ_LF_ and Δ_1,2_) for both Nd^3+^ and Dy^3+^, increased weight of $B_{q}^{6}$ terms, reduced contribution of axial terms in the LF, reduced size of $B_{0}^{2}$ , reduced weight of |±9/2⟩ in Nd^3+^–KD1, increased *g_x_*_,_ *_y_* values (twice for Nd^3+^ and by 1 order of magnitude for Dy^3+^), and reduced *g_z_* for Nd^3+^. All these differences are signatures of the lower magnetic axiality in ^Ad^M. The recent computational study of DySc_2_N@C_80_(CF_2_),^74,128^ in which CF_2_ group was added in the [6,6]-open position as in the experimentally available Sc_3_N@C_80_(CF_2_),^64^ also demonstrated a considerable reduction of the LF splitting in ^CF2^Dy coordination in comparison to ^CF2^Sc. The authors ascribed this phenomenon to the electron-withdrawing effect of fluorine atoms and the orientation of the positive side of the CF_2_ dipole toward Dy. However, our results demonstrate a similar reduction of the LF splitting for the electron-donating Ad moiety. Therefore, it appears to be a general property of the lanthanide coordination to a functionalized fullerene fragment with an open bond, caused by the close-to-equatorial positions of the nearest carbon atoms.

Experimental verification of the lower LF splitting in ^Ad^Nd than in ^Ad^Sc conformers of NdSc_2_N@C_80_(Ad) is provided by the PL spectroscopy of **Nd-II**, for which NMR suggests the presence of both forms. Assuming similar energies of KD1 and using CASSCF/RASSI-SO energies of KDs in ^4^I_9/2_ and ^4^I_11/2_ multiplets as a guide, we can tentatively assign all peaks to either ^Ad^Sc or ^Ad^Nd conformers (Figure 7b). Low-energy peaks in the spectra of **Nd-II**, which are absent for **Nd-I**, are especially characteristic of the ^Ad^Nd form. Computed KD energies also allow us to estimate the components of magnetic susceptibility tensors, which affect paramagnetic chemical shifts (eq 1). For the lowest-energy [6,6]-^Ad^M conformers, the theoretical ratio of (*χ*_∥_ − *χ*_⊥_) values for Dy and Nd at 298 K is 17.3, reasonably close to the experimental ratio of the chemical shifts of the ^Ad^Dy and ^Ad^Nd forms.

#### Single-Molecule Magnetism of DySc_2_N@C_80_(Ad)

Figure 9 summarizes the SMM properties of **Dy-I** and **Dy-II** studied by SQUID magnetometry in comparison to the nonfunctionalized fullerene. The peak in the temperature dependence of the magnetization measured for a zero-field-cooled (ZFC) sample of pristine DySc_2_N@C_80_ occurs at 6.9 K and coincides with the bifurcation point of the ZFC and FC (field-cooled) curves. For **Dy-I**, an analogous peak is shifted to *T*_B_ = 9.0 K, and bifurcation occurs at *T*_irrev_ = 10 K. The ZFC curve of **Dy-II** has a more complex shape, with the peak at 4.6 K, a broad feature near 8 K, and ZFC/FC bifurcation at 11 K. As shown by NMR spectroscopy, **Dy-I** consists of the single [5,6]-^Ad^Sc form, and we can definitely conclude that carbene addition across the [5,6] bond increases the blocking temperature of magnetization for the ^Ad^Sc coordination. The complex shape of the **Dy-II** ZFC curve is evidently caused by the presence of [6,6]-^Ad^Sc and [6,6]-^Ad^Dy forms with distinct relaxation behavior. By analogy with **Dy-I**, and based on the results of *ab initio* calculations, we propose that the blocking temperature of the [6,6]-^Ad^Sc form is increased in comparison to DySc_2_N@C_80_, whereas that of [6,6]-^Ad^Dy is decreased and corresponds to the ZFC peak at 4.6 K.

**Figure 9:**
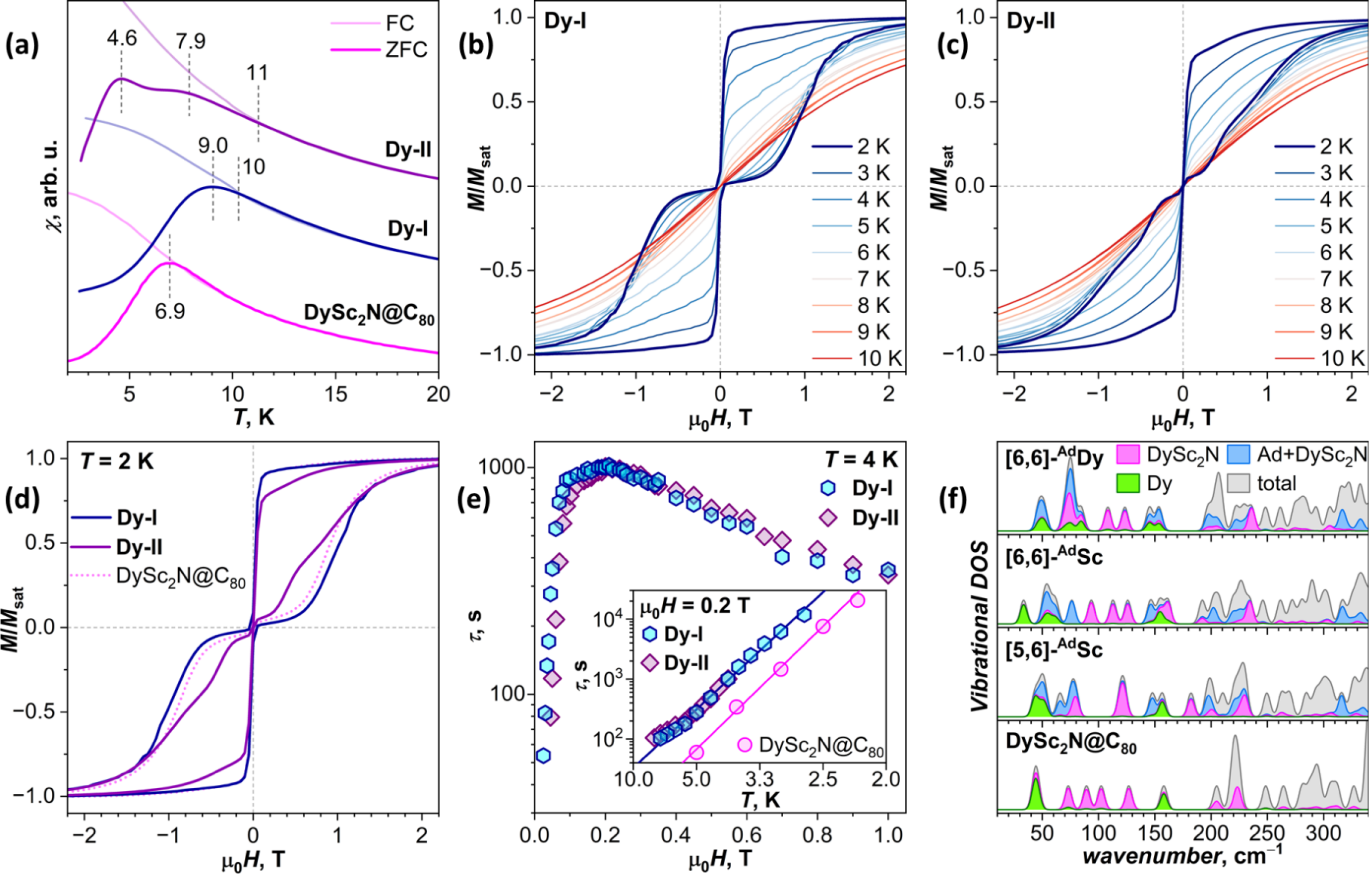
(a) Blocking temperature from ZFC/FC measurements (μ_0_ *H* = 0.2 T, temperature sweep rate 5 K min^–1^); (b) magnetic hysteresis of **Dy-I** at different temperatures; (c) magnetic hysteresis of **Dy-II** at different temperatures; (d) magnetic hysteresis of **Dy-I**, **Dy-II**, and DySc_2_N@C_80_ measured at 2 K. In (b–d), the average magnetic field sweep rate is 2.9 mT s^–1^. (e) Field dependence of magnetization relaxation time of **Dy-I** and **Dy-II** at 4 K, inset shows temperature dependence of magnetization relaxation time of **Dy-I** and **Dy-II** in the field of 0.2 T. Also shown are relaxation times of nonderivatized DySc_2_N@C_80_ from ref 75 and linear fits in Arrhenius coordinates. (f) Total and partial vibrational density of states in the low-frequency range computed for DySc_2_N@C_80_ and isomers of DySc_2_N@C_80_(Ad); VDOS is obtained by convoluting frequencies computed for isolated molecules with a Gaussian function.

**Dy-I** and **Dy-II** show butterfly (waist-restricted) magnetic hysteresis with an abrupt change in magnetization near zero field (Figure 9b,c), which is characteristic of the vast majority of single-ion Dy SMMs and is caused by the quantum tunneling of magnetization. Narrow opening of the hysteresis is preserved up to 10 K, in line with *T*_irrev_ values of 10–11 K. The hysteresis of **Dy-I** is slightly broader than that of pristine DySc_2_N@C_80_ (Figure 9d). In the hysteresis of **Dy-II**, two components with narrower and broader widths are distinguishable, presumably corresponding to ^Ad^Dy and ^Ad^Sc forms, respectively.

Magnetization relaxation times τ of **Dy-I** and **Dy-II** were measured using the DC technique (Tables S7 and S8, and Figure S19 and S20). Figure 9e shows the field dependence of τ at 4 K. The contribution of the ^Ad^Dy form in **Dy-II** at this temperature should be already negligible. Nearly identical dependencies of two isomers indicate that ^Ad^Sc forms have very similar relaxation characteristics, independent of the carbene addition site. The longest times are measured near 0.2 T, and this field was chosen to study the temperature dependence (Figure 9e, inset). In the temperature range accessible for DC technique, **Dy-I** shows 5-fold longer relaxation times than DySc_2_N@C_80_. The dependence is linear in Arrhenius coordinates and can be described by the local mode mechanism with a frequency of 22.5(6) K (15.6 cm^–1^) and an attempt time of 3.5(6) s. DySc_2_N@C_80_ gives a similar frequency of 23.6 K (16.4 cm^–1^), but a shorter attempt time of 0.6 s.^75^ Relaxation times of **Dy-II** measured above 4 K coincide with those of **Dy-I**, indicating once again that the relaxation times of ^Ad^Sc forms do not depend on the isomerism of Ad addition. Yet, they are longer than in the pristine DySc_2_N@C_80_ despite coordination of Dy to the nonfunctionalized fragment of the cage.

Linear regimes in the temperature dependence of relaxation times were observed for a number of Dy metallofullerenes and are presumably associated with low-frequency metal-based vibrations.^10,17,18,27,47,53,75^ Figure 9f compares total and partial vibrational density of states calculated for [5,6]-^Ad^Sc, [6,6]-^Ad^Sc, [6,6]-^Ad^Dy, and DySc_2_N@C_80_ (note that vibrational calculations were performed at the PBE/TZ2P level for Y analogs but using atomic mass of Dy). In the pristine fullerene, cluster modes are mainly found below 200 cm^–1^. Three modes with dominant Dy contributions occur at 43, 46, and 158 cm^–1^. The former two can be described as lateral (vibrational displacement of Dy is parallel to the cage surface, DySc_2_N cluster librates as a whole), and the higher-frequency one has longitudinal character (displacements normal to the fullerene surface, mainly Dy–cage and Dy–N stretching). Carbene addition introduces new low-frequency modes of the Ad moiety and changes potential energy surface for the endohedral cluster, which can affect its vibrational frequencies. In [5,6]-^Ad^Sc, the modes with Dy contributions at 44, 52, and 157 cm^–1^ mainly remain intact except for a mixing of one lateral vibration with Ad mode and its slight upshift. In [6,6]-^Ad^Sc, the effect of the derivatization is more pronounced, as one lateral mode shifts down to 33 cm^–1^, while another one is strongly mixed with Ad modes and is distributed over three vibrations at 52, 55, and 62 cm^–1^. Longitudinal mode is also partially mixed with Ad but retains the main component at 155 cm^–1^. Trapping Dy in the Ad pocket in [6,6]-^Ad^Dy results in stronger mixing with Ad vibrations and moves the center of gravity to higher frequencies. The main contribution of lateral Dy displacements is found at 47, 51, 70, 75, and 84 cm^–1^. The frequency of the longitudinal mode is less affected and remains near 150 cm^–1^.

Contribution of a given vibrational mode with frequency *v* to the spin–lattice relaxation rate can be presented as a product of the spin-phonon coupling parameter *V*_sp‑ph_ and a thermal factor, *V*_sp‑ph_exp­(*hv*/*k*_B_ *T*)­{exp­(*hv*/*k*_B_ *T*)–1}^−2^. The latter favors low-frequency modes, especially at low temperature. Thus, lateral modes should be most critical for the relaxation of magnetization in metallofullerenes.^52^ However, local mode frequencies deduced from relaxation times are significantly lower than the computed vibrational frequencies. This discrepancy can be caused by the use of a harmonic approximation in vibrational calculations, which cannot be very accurate for low-frequency modes with shallow curvature of the potential energy surface. Besides, consideration of the finite phonon lifetime was found to change the barrier to half of the vibrational frequency.^129^ On the other hand, our calculations are performed for isolated molecules, while in the crystalline phase the metal-based vibrations were found to efficiently mix with lattice phonons. Importance of such mode mixing for spin–lattice relaxation was discussed in refs 53,130 ,^131^. Finally, if the analysis is limited solely to the thermal factor, the increase in lateral mode frequencies for the ^Ad^Dy conformer would be expected to slow down the relaxation; however, an opposite trend is observed experimentally. Deeper evaluation of these factors requires calculations of phonon band structure and spin–phonon coupling parameters, which remain computationally demanding for molecules of this size and will require a separate dedicated study.

## Conclusions

In this work, we analyzed how chemical derivatization of the fullerene cage by carbene addition affects the single-ion magnetic anisotropy and SMM properties of endohedral lanthanides. For the case study, we used mixed-metal nitride clusterfullerenes MSc_2_N@C_80_, which were chemically modified by addition of an adamantylidene group. The choice of lanthanides Nd and Dy was dictated by their strong eas*y*-axis magnetic anisotropy in pristine nitride cluster fullerenes MSc_2_N@C_80_, and by the possibility to use spectroscopic and magnetometry techniques: Nd forms weak SMMs, but its ligand-field splitting in EMFs can be directly addressed by NIR photoluminescence, whereas Dy is not accessible by Dy-PL, but forms robust SMMs, which can be comprehensively analyzed by SQUID magnetometry.

For both NdSc_2_N@C_80_ and DySc_2_N@C_80_, Ad addition gave [5,6]-open and [6,6]-open isomers of MSc_2_N@C_80_(Ad). Proton nuclear spins of the Ad moiety, in combination with strong lanthanide-induced pseudocontact paramagnetic shifts, proved to be a powerful spin probe for the location of lanthanide ions. Paramagnetic ^1^H NMR clearly demonstrated that the Ad-addition site in [5,6] isomers is coordinated only by Sc, whereas Sc and lanthanide coordination coexist in [6,6] isomers. Inseparable ^Ad^Sc and ^Ad^M conformers complicate the study of [6,6] isomers but at the same time enable direct comparison of two conformations in the same settings.

In ^Ad^Sc conformers, the Ad addition site is located remotely from the lanthanide and has no direct influence on the latter. The lanthanide-cage interactions should not differ significantly from those in pristine MSc_2_N@C_80_. However, there is an indirect effect of the derivatization, manifesting in the reduced conformational freedom of the cluster in MSc_2_N@C_80_(Ad) and stabilization of the specific conformer for the [5,6] isomer in which the M-cage coordination geometry provides the strongest magnetic axiality. Experimentally it is observed as the enhanced LF splitting in PL spectra of [5,6]-NdSc_2_N@C_80_(Ad) and the increased blocking temperature of magnetization, along with longer relaxation times in ^Ad^Sc conformers of both [5,6] and [6,6] isomers of DySc_2_N@C_80_(Ad).

In the ^Ad^Nd and ^Ad^Dy conformers, the lanthanide coordination to the Ad-addition site has a direct influence on the magnetic properties. The carbons nearest to the lanthanide in this coordination geometry are located in semiequatorial positions, which results in the weakening of the magnetic axiality and a considerable reduction of the LF splitting, visible both in calculations and in Nd-PL spectra. For the SMM properties of DySc_2_N@C_80_(Ad), this leads to faster spin–lattice relaxation and a noticeable reduction in the blocking temperature of magnetization.

These findings demonstrate that there is no simple answer to the question, “How does derivatization of the fullerene affect the magnetic anisotropy of endohedral lanthanides?” Direct and indirect effects of carbene addition are opposite, and the outcome depends on the regio-isomerism and the realization of particular conformers, which may differ considerably in their magnetic properties. Chemical derivatization of EMFs can indeed become a useful tool to improve their SMM performance, but careful evaluation of different factors is always needed. Our results on the adamantylidene addition can be generalized to all types of [2 + 1] cycloaddition producing methanofulleroids with an open C–C bond, but other reactions, such as the cycloaddition occurring without opening cage C–C bonds, will require an independent study. Finally, we should note that derivatization of the carbon cage also affects vibrational modes, which can alter spin-phonon coupling and, thereby, relaxation of magnetization. A simple consideration of vibrational frequencies in this work was inconclusive, suggesting that a full analysis of spin-phonon coupling is necessary to evaluate this factor.

## Supplementary Material



## Notes

Experimental and computational details, HPLC separation, additional NMR and PL spectra, PL decays, additional computational results, and magnetization relaxation measurements (PDF)
