# Supplementary material for: Controlling Magnetic Anisotropy of Endohedral Lanthanide Ions by Carbene Addition: Paramagnetic NMR, Lanthanide Luminescence, and Single-Molecule Magnetism in Adamantylidene Adducts of MSc2N@C80 (M = Nd, Dy)
Source: J Am Chem Soc. 2025 Sep 3;147(37):33812–27. doi: 10.1021/jacs.5c10147 (PMC12447498; doi:10.1021/jacs.5c10147)
Supplement: Supplementary file 1 [file ja5c10147_si_001.pdf]

Controlling magnetic anisotropy of endohedral lanthanide ions by carbene addition: Paramagnetic NMR, lanthanide luminescence, and single-molecule magnetism in adamantylidene adducts of $MSc_2N@C_{80}$ (M = Nd, Dy)

Wei Yang,[$a] Matheus Felipe de Souza Barbosa,[$a] Noel Israel,[a] Marco Rosenkranz,[a] Fupin Liu,[b] Stanislav M. Avdoshenko,[a]* Alexey A. Popov[a]*

[a] *Leibniz Institute for Solid State and Materials Research (IFW Dresden), Helmholtzstr. 20, 01069, Dresden, Germany*

[b] *Jiangsu Key Laboratory of New Power Batteries, Jiangsu Collaborative Innovation Center of Biomedical Functional Materials, School of Chemistry and Materials Science, Nanjing Normal University, Nanjing, 210023 China*

Supporting information

Experimental details	S2
Synthesis and separation	S4
NMR spectra and chemical shifts	S5
DFT calculations	S8
Photoluminescence spectra and PL decays	S12
CASSCF calculations	S15
Magneto-structural correlations	S25
Magnetization relaxation times	S33
References	S37

Experimental details

Synthesis of AdN_2: 2-adamantane-2,3'-[3H]-diazirine was synthesized from 2-adamantanone following literature methodics.[1, 2] 2-Adamantanone (1.05 g) was stirred with ammonia in methanol (25% w/v) for 2 hours at −10 °C. One gram of freshly prepared hydroxylamine-O-sulfonic acid in 10 mL of methanol was added dropwise. The mixture was stirred for 24 h at 4 °C. Some ice was added to the flask to cool the solution and the flask was placed into an ice bath for keeping low temperature. Then, NaClO was added (180 mL; 6% w/v; 5 eq.) and the mixture was stirred vigorously for 2 hours. Afterwards, the product was purified by elution with n-hexane:

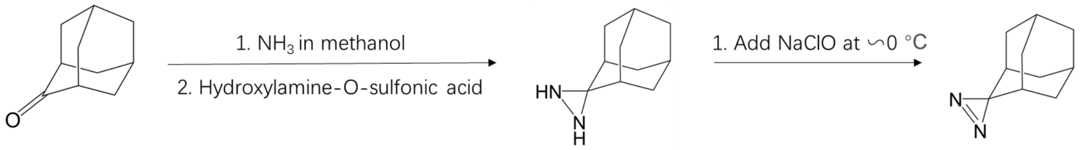

Synthesis of $MSc_2N@C_{80}(Ad)$: ~1.2 mg of endohedral metallofullerene (~1×10^{-3} mmol) and 20 equiv. of AdN_2 (2×10^{-2} mmol, 3.24 mg) were dissolved in 30 mL of anhydrous toluene and placed in a Schlenk tube. The mixture was degassed by freeze-pump-thaw cycles under reduced pressure for removing O_2 and then irradiated with a 365 nm LED light (LightningCure LC-L1 from Hamamatsu) at room temperature under N_2 atmosphere, with continuous stirring throughout the reaction. During the reaction, 0.5 mL aliquots of the solution were withdrawn using a syringe for chromatographic analysis (Buckyprep column, Φ = 10 mm × 250 mm, elution with toluene) at specified time intervals. To prevent significant build-up of bis-adducts, irradiation was stopped when conversion of the pristine fullerene was 50−75%. Two isomers of Ad monoadduct were then obtained by separation with linear and recycling HPLC, and their composition was identified by MALDI mass-spectrometry.

$NdSc_2N@C_{80}(Ad)$: based on HPLC peak areas, conversion of $NdSc_2N@C_{80}$ was 47%; net yield of both $NdSc_2N@C_{80}$(Ad) isomers 30% (63% if counted on reacted $NdSc_2N@C_{80}$), yield of $NdSc_2N@C_{80}$(Ad) isomers based on reacted $NdSc_2N@C_{80}$: 24% for Nd-I, 39% for Nd-II.

$DySc_2N@C_{80}(Ad)$: based on HPLC peak areas, conversion of $DySc_2N@C_{80}$ was 78%; net yield of both $DySc_2N@C_{80}$(Ad) isomers 34% (44% if counted on reacted $DySc_2N@C_{80}$), yield of $DySc_2N@C_{80}$(Ad) isomers based on reacted $DySc_2N@C_{80}$: 12% for Dy-I, 32% for Dy-II.

HPLC: HPLC analysis and separation were performed for toluene solutions of fullerene and with toluene as an eluent, employing semipreparative COSMOSIL Buckyprep chromatographic columns (Nacalai Tesque) and Agilent 1260 Infinity II LC System. Recycling HPLC separation was performed using Sunflow 100 system (SunChrome).

Mass spectrometry: Matrix-assisted laser desorption/ionization time-of-flight (MALDI-TOF) mass-spectra were measured with a Bruker autoflex mass-spectrometer.

UV-Vis spectrometry: UV-vis-NIR absorption spectra were measured in CS_2 solution at room temperature with Shimadzu 3100 spectrophotometer.

NMR spectroscopy: Variable-temperature ^{1}H NMR spectra were measured with 500 MHz Avance II spectrometer (Bruker) in CS_2 solution, in a concentration of ~0.5 mg/mL. For spectra measured in extended range (several hundred ppm), different phase correction had to be used in different parts of the spectra.

Photoluminescence spectroscopy. For photoluminescence measurements, fullerene samples were drop-casted from toluene solution onto KBr substrate producing microcrystalline films. NIR-PL spectra were

measured with a modified fluorescence microscope (Olympus BX series) using excitation at 488 nm from Omicron PhoxX diode laser and detected with Andor iDus 1.7 µm InGaAs camera attached to Kymera 328i spectrograph (Andor); temperature was controlled with Janis ST-500 microscopy cryostat. The photoluminescence decay measurements were performed using time-correlated single-photon counting (TCSPC) with ID900 Time Controller and ID230 NIR single-photon counter (both from ID Quantique) and 488 nm Omicron PhoxX diode laser, which was digitally modulated to give the pulse width of 1 ns.

Magnetic measurements. Magnetic measurements of powder samples were performed using a Quantum Design VSM MPMS3 magnetometer.

DFT computations. DFT optimization of $YSc_2N@C_{80}$(Ad) conformers was performed at the PBE level with implemented TZ2P-quality basis set for C, N, and H, and ECP basis set for Y using Priroda code.[3, 4] For the QTAIM analysis, wavefunction was computed with full-electron TZVP-quality basis set and PBE0 functional using Orca suite;[5] topological analysis of the electron density was then performed with AIMAll code. Conformers selected for CASSCF calculations were optimized at the PBE/PAW level using the VASP code and recommended pseudopotentials with f-shell in-core treatment for Dy and Nd.[6-10]

CASSCF calculations. *Ab initio* energies and wave functions of Dy^{3+} and Nd^{3+} LF multiplets in $MSc_2N@C_{80}$(Ad) molecules have been calculated at the CASSCF/SO-RASSI level of theory using the quantum chemistry package OpenMOLCAS[11] and SINGLE_ANISO module.[12] The basis sets were ANO-RCC-VTZP for Dy and Nd and ANO-RCC-VDZP for other elements.

Synthesis of $NdSc_2N@C_{80}(Ad)$

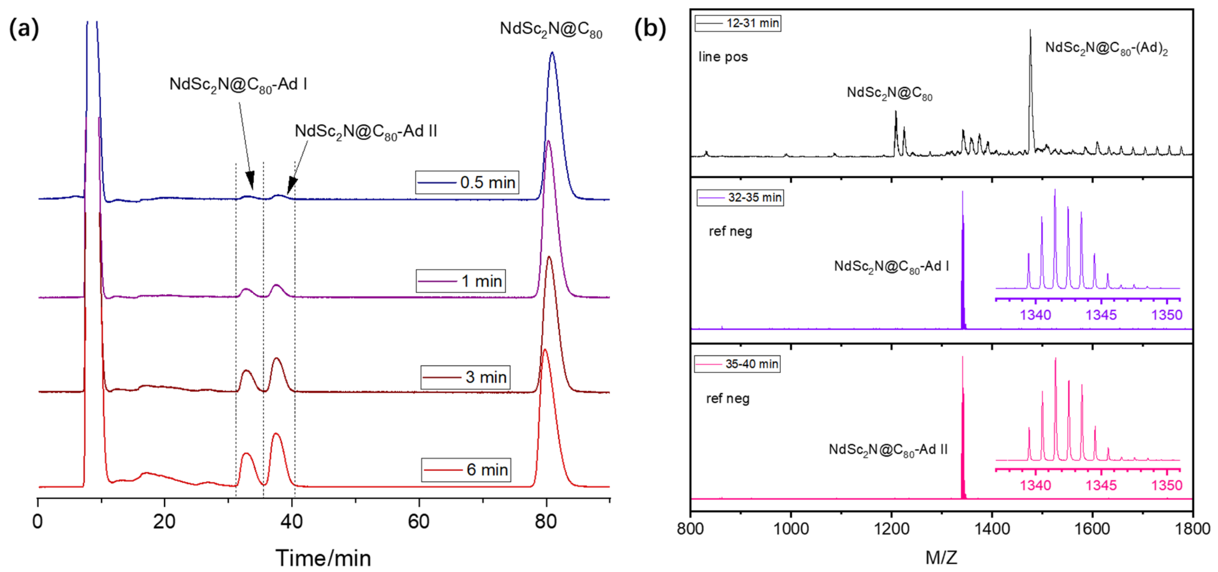

Figure S1. (a) HPLC traces measured at different moments of time during the photochemical reaction between $NdSc_2N@C_{80}$ and AdN_2 (2×Buckyprep columns, 5 mL/min). (b) Negative-ion MALDI mass spectra of isolated $NdSc_2N@C_{80}(Ad)$ isomers and of the fraction collected between 12 and 31 min showing bis-adduct signal.

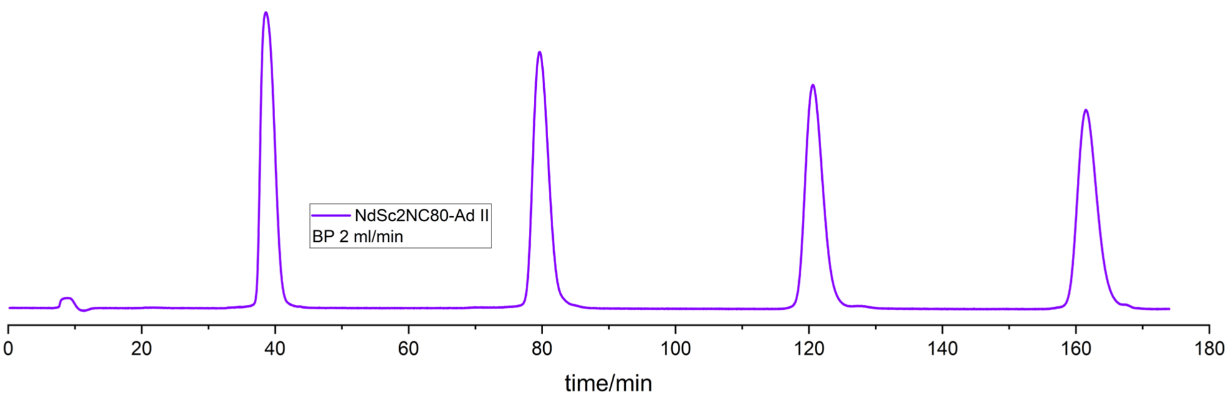

Figure S2. Recycling HPLC (Buckyprep column, 2 mL/min) of **Nd-II**.

Synthesis of $DySc_2N@C_{80}(Ad)$

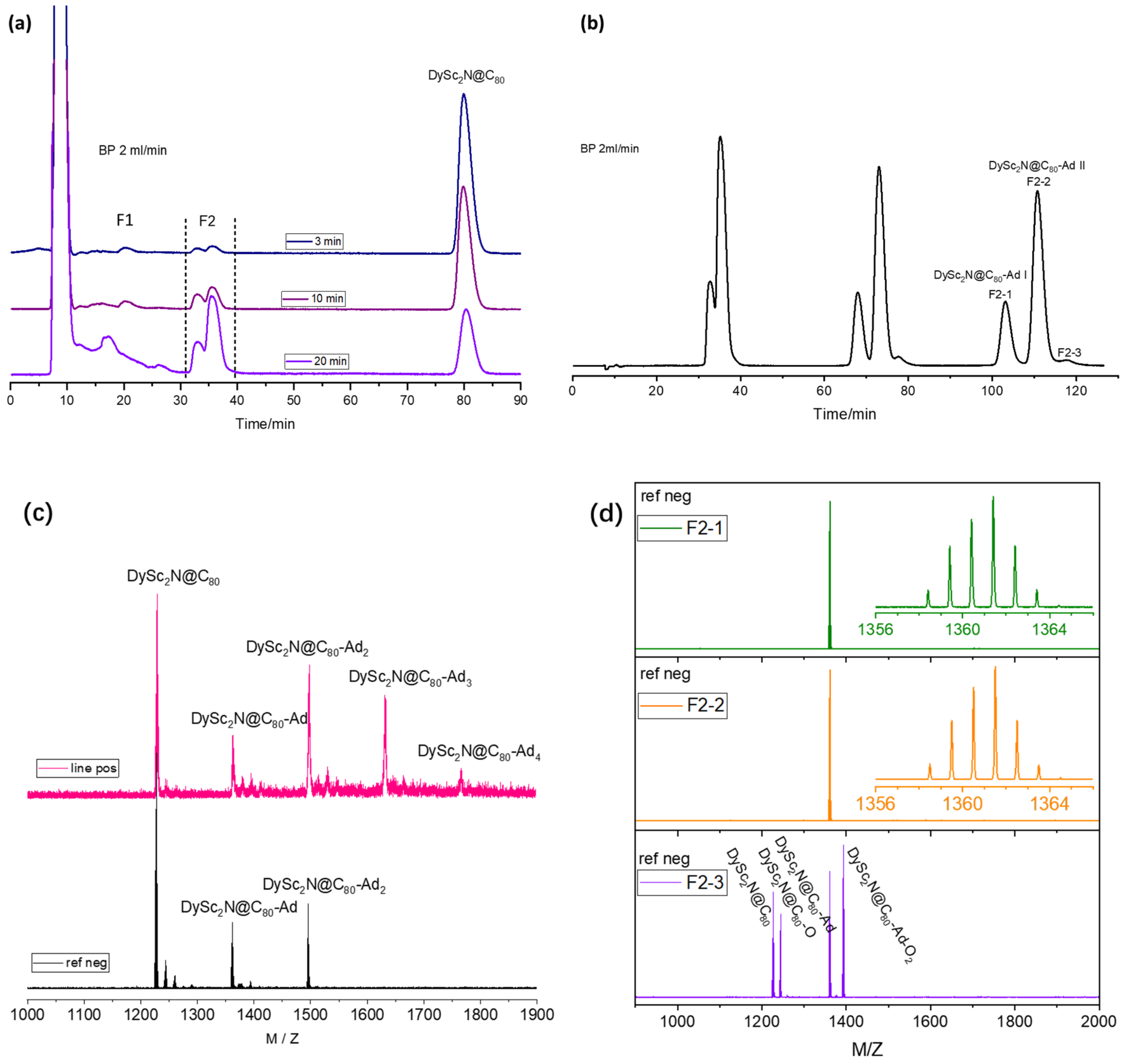

Figure S3. (a) HPLC traces measured at different moments of time during the photochemical reaction between $DySc_2N@C_{80}$ and AdN_2 (2×Buckyprep columns, 5 mL/min). (b) Recycling HPLC (Buckyprep column, 2 mL/min) of fraction F2, containing a mixture of $DySc_2N@C_{80}(Ad)$ isomers. (c) Negative and positive-ion MALDI mass spectra of the fraction collected between 12 and 31 min showing bis and poly-adduct signal. (d) Negative-ion MALDI mass spectra of isolated $DySc_2N@C_{80}(Ad)$ isomers (fractions F2-1 ad F2-2) and minor fraction F2-3 with signatures of oxidation.

Variable-temperature ^{1}H NMR

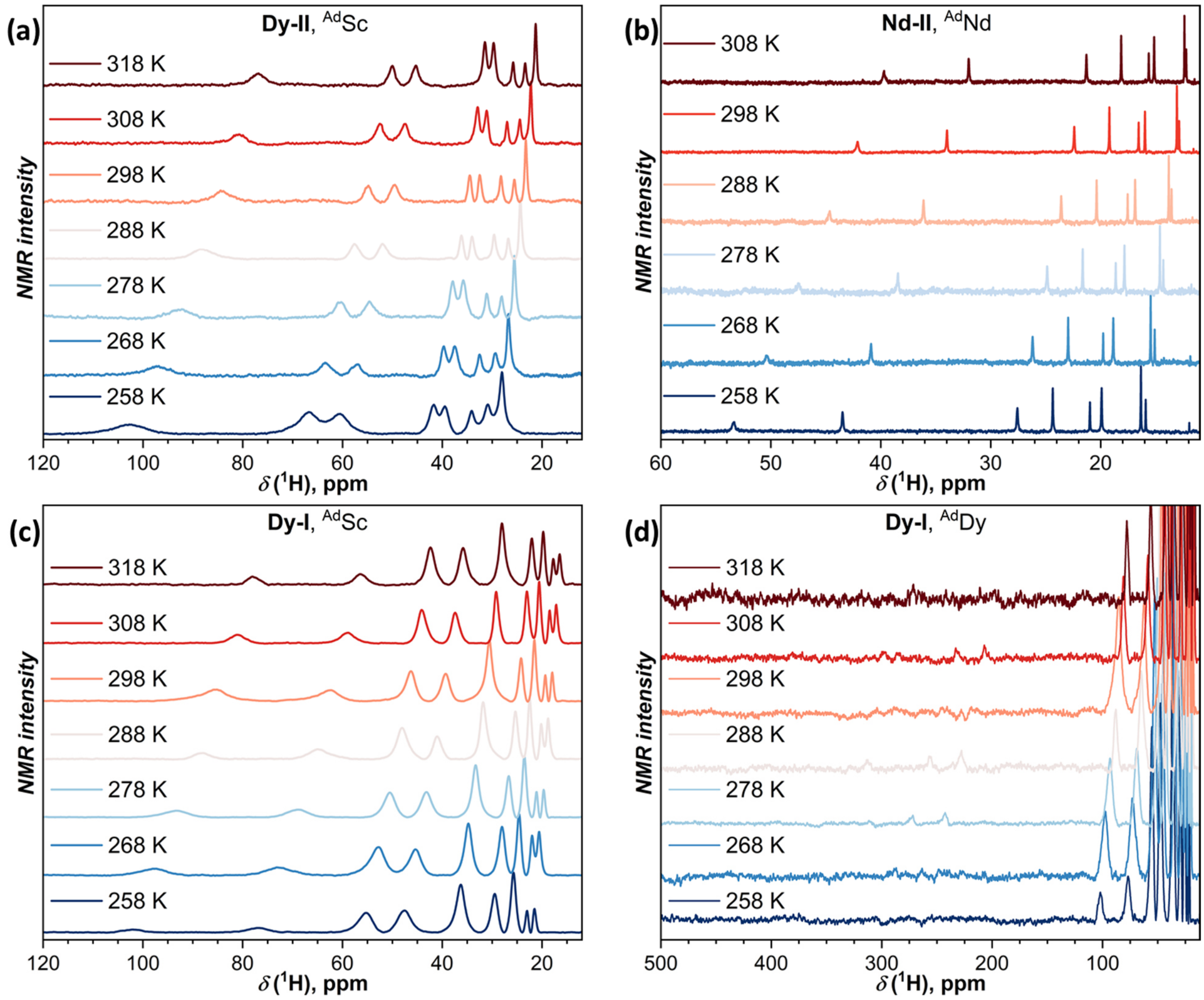

Figure S4. Variable-temperature ^{1}H NMR spectra: (a) **Dy-II**, ^{Ad}Sc range; (b) **Nd-II**, ^{Ad}Nd range; (c) **Dy-I**, ^{Ad}Sc range; (d) **Dy-I**, ^{Ad}Sc and ^{Ad}Dy range. For **Dy-I**, weak signals of the ^{Ad}Dy form are detected at 200–350 ppm, but the dominant signals are from the ^{Ad}Sc form.

According to Bleaney's theory, lanthanide-induced pseudocontact chemical shifts scale with temperature as T^{-2}.[13, 14] If contact shifts can be considered negligible, as we suggest for $DySc_2N@C_{80}$(Ad) and $NdSc_2N@C_{80}$(Ad), then a simple equation is fulfilled: $\delta_{para} = \delta_{dia}+CT^{-2}$, where C is the temperature-independent parameter. Extrapolation of linear dependencies to $T^{-2} = 0$ should then give diamagnetic shifts, which for Ad are in the range of 2–3 ppm.[15] Experimentally measured temperature dependencies shown in Figure S5 do not extrapolate to expected values, indicating that the T^{-2} dependence does not hold well.

Table S1. ^{1}H chemical shifts in **Dy-I**, **Dy-II**, and **Nd-II** at 298 K

	**Dy-I**		**Dy-II**		**Nd-II**	**Dy-II/ Nd-II**[a]
	^{Ad}Sc		^{Ad}Dy	^{Ad}Sc	^{Ad}Nd	^{Ad}Ln
e	18.0	***e***	219.1	23.3	13.1	20.6
e'	19.3	***d***	225.8	25.6	12.9	21.7
d	21.5	***d'***	269.6	28.3	16.6	19.1
c	24.2	***c***	287.6	32.6	16.0	21.3
c'	30.6	***c'***	326.8	34.5	19.2	19.5
b	39.4	***b***	393.5	49.8	22.4	19.7
b'	46.3	***b'***	533.5	55.0	34.0	16.9
a	62.4	***a***	801.9	84.6	42.1	20.2
a'	85.4					

[a] an average diamagnetic chemical shift of 2.6 ppm was subtracted from chemical shifts of **Dy-II** and **Nd-II** before calculating this ratio.

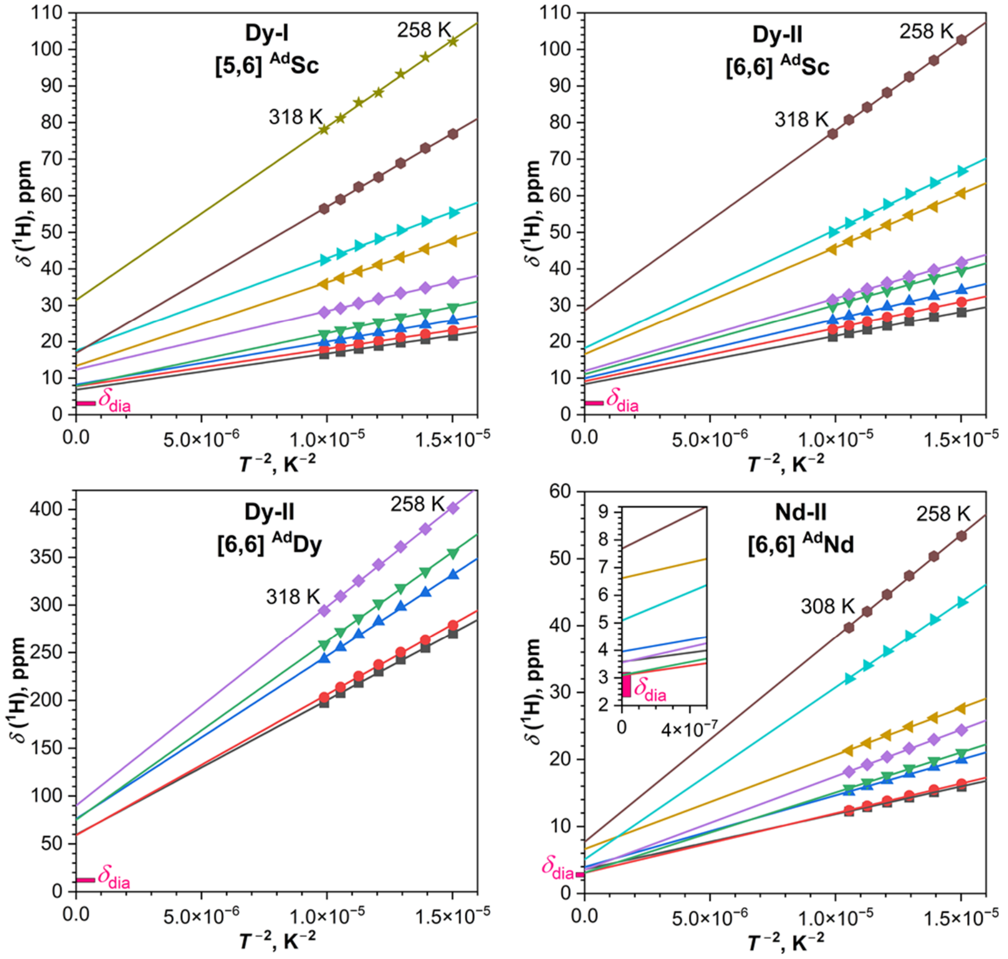

Figure S5. Temperature dependence of chemical shifts versus T^{-2} and extrapolation to $T^{-2} = 0$; δ_{dia} denotes the range of ^{1}H chemical shifts in diamagnetic $M_3N@C_{80}(Ad)$ compounds (M = Sc, Lu).[15]

DFT calculations of molecular structures

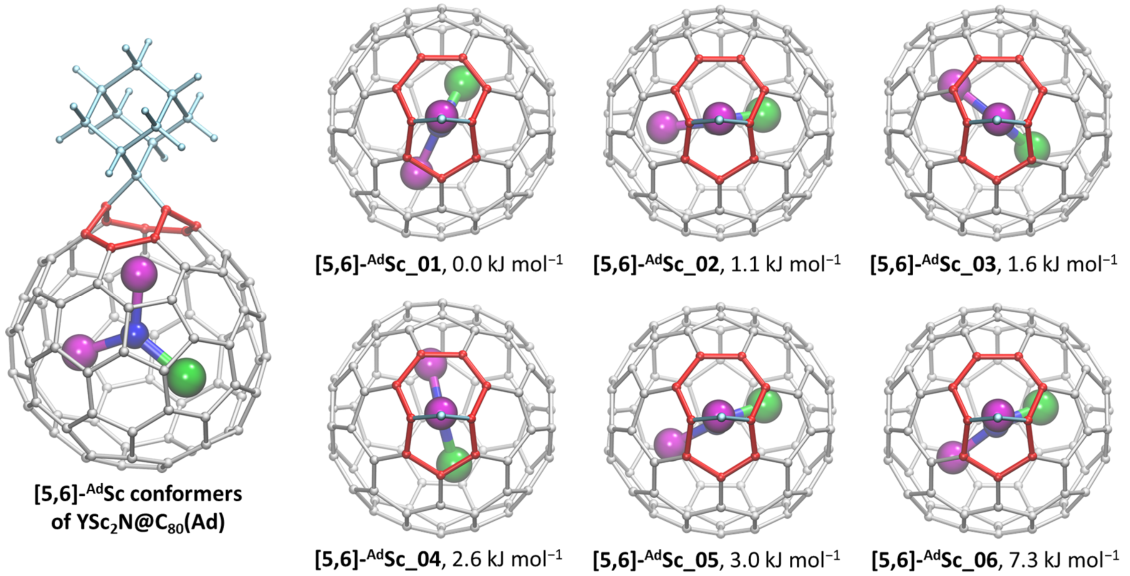

Figure S6a. Molecular structure of six unique [5,6]-^{Ad}Sc conformers of $YSc_2N@C_{80}$(Ad) and their relative energies (PBE/TZ2P). In the top view, Ad moiety is omitted for clarity as the structures only differ in the rotation angle around the ^{Ad}Sc–N bond. Color code: Y – green, Sc – magenta, N – blue, C – light gray, Ad – light cyan, pentagon and hexagon at the [5,6]-open edge are highlighted in red.

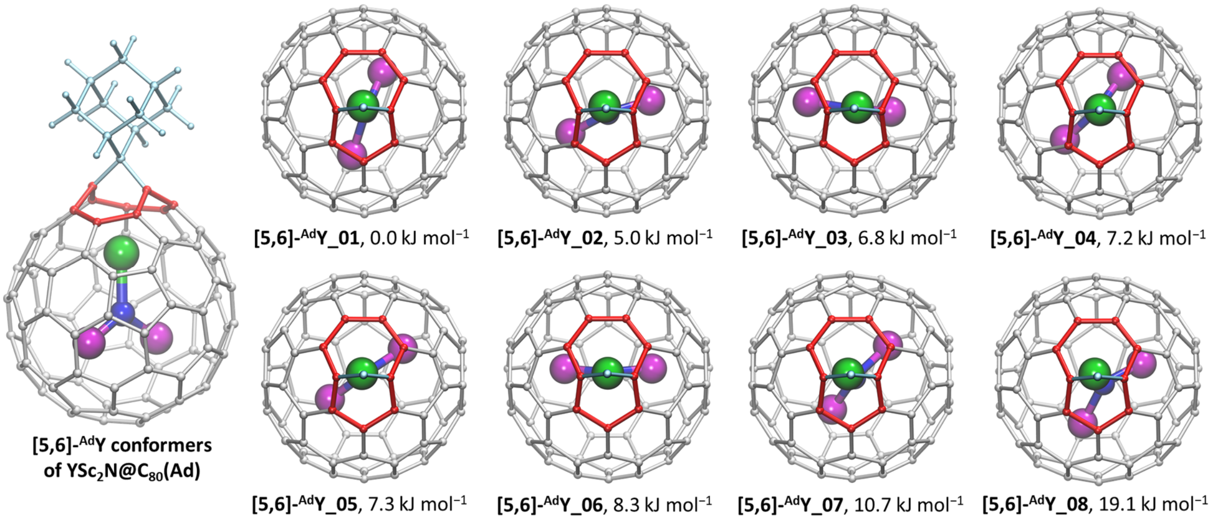

Figure S6b. Molecular structure of eight unique [5,6]-^{Ad}Y conformers of $YSc_2N@C_{80}$(Ad) and their relative energies (PBE/TZ2P). In the top view, Ad moiety is omitted for clarity as the structures only differ in the rotation angle around the ^{Ad}Y–N bond. Color code: Y – green, Sc – magenta, N – blue, C – light gray, Ad – light cyan, pentagon and hexagon at the [5,6]-open edge are highlighted in red.

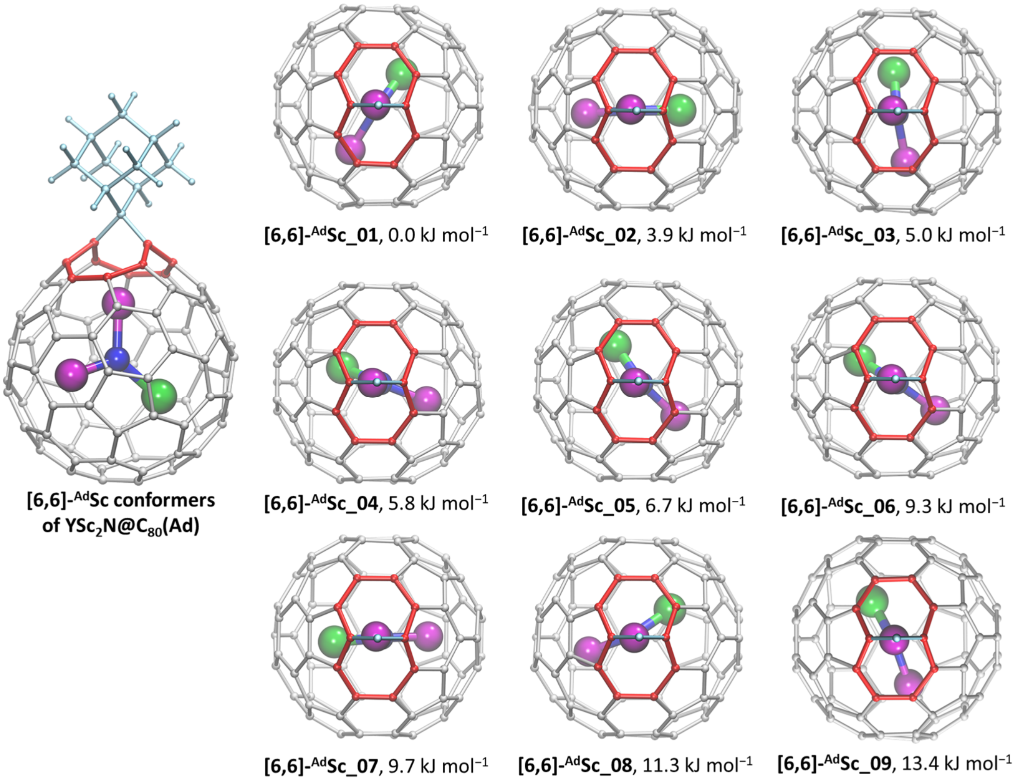

Figure S6c. Molecular structure of nine unique [6,6]-^{Ad}Sc conformers of $YSc_2N@C_{80}$(Ad) and their relative energies (PBE/TZ2P). In the top view, Ad moiety is omitted for clarity as the structures only differ in the rotation angle around the ^{Ad}Sc–N bond. Color code: Y – green, Sc – magenta, N – blue, C – light gray, Ad – light cyan, hexagons at the [6,6]-open edge are highlighted in red.

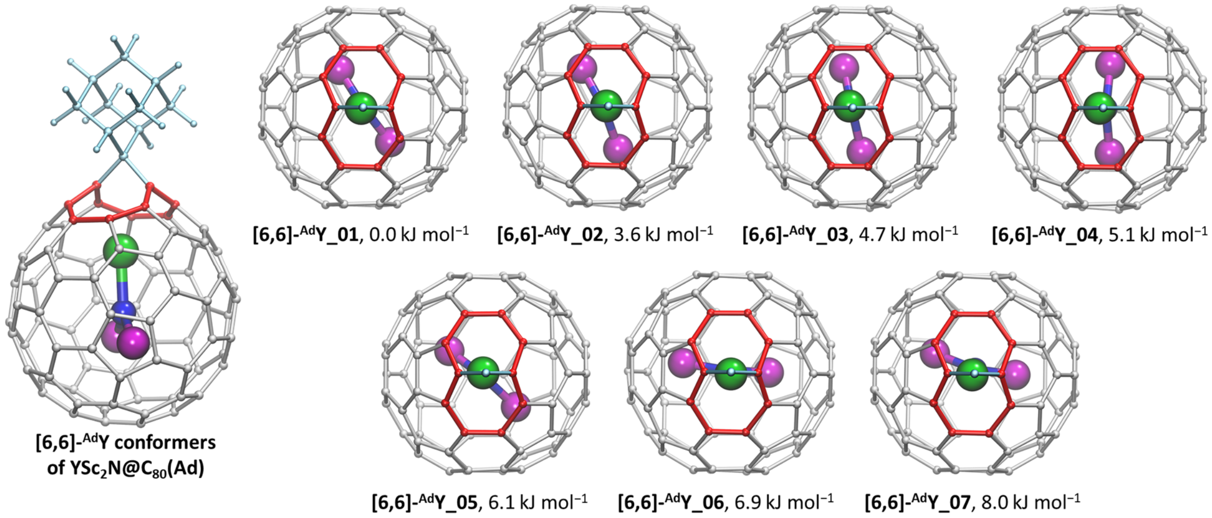

Figure S6d. Molecular structure of seven unique [6,6]-^{Ad}Y conformers of $YSc_2N@C_{80}$(Ad) and their relative energies (PBE/TZ2P). In the top view, Ad moiety is omitted for clarity as the structures only differ in the rotation angle around the ^{Ad}Y–N bond. Color code: Y – green, Sc – magenta, N – blue, C – light gray, Ad – light cyan, hexagons at the [6,6]-open edge are highlighted in red.

Table S2. Relative energies and M–N bond lengths in isomers and conformers of $MSc_2N@C_{80}$(Ad) (M = Y, Dy, Nd)

	M = Y		M = Dy		M = Nd	
	Δ*E*, kJ mol^{-1}	$d_{Y–N}$, Å	Δ*E*, kJ mol^{-1}	$d_{Dy–N}$, Å	Δ*E*, kJ mol^{-1}	$d_{Nd–N}$, Å
**[5,6]-$MSc_2N@C_{80}$(Ad)**						
[5,6]-^{Ad}Sc_01	0.0	2.185	1.2	2.187	1.0	2.245
**[5,6]-^{Ad}Sc_02**	**1.1**	**2.186**	**0.0**	**2.191**	**0.0**	**2.248**
[5,6]-^{Ad}Sc_03	1.6	2.189	2.0	2.191	0.1	2.247
[5,6]-^{Ad}Sc_04	2.6	2.178	0.0	2.181	4.1	2.240
[5,6]-^{Ad}Sc_05	3.0	2.189	0.7	2.191	1.1	2.252
[5,6]-^{Ad}Sc_06	7.2	2.180	9.3	2.183	8.1	2.244
[5,6]-^{Ad}M_01	5.4	2.195	1.3	2.197	8.1	2.256
[5,6]-^{Ad}M_02	10.4	2.201	4.9	2.201	10.4	2.261
[5,6]-^{Ad}M_03	12.2	2.202	8.0	2.204	12.6	2.259
[5,6]-^{Ad}M_04	12.6	2.194	9.7	2.196	15.2	2.253
[5,6]-^{Ad}M_05	12.7	2.203	6.1	2.207	13.1	2.266
[5,6]-^{Ad}M_06	13.7	2.199	7.9	2.203	16.0	2.265
[5,6]-^{Ad}M_07	16.1	2.197	10.9	2.198	17.0	2.255
[5,6]-^{Ad}M_08	24.5	2.178	23.8	2.177	32.4	2.250
**[6,6]-$MSc_2N@C_{80}$(Ad)**						
**[6,6]-^{Ad}Sc_01**	**0.0**	**2.187**	**4.3**	**2.189**	**0.0**	**2.247**
[6,6]-^{Ad}Sc_02	3.9	2.200	9.7	2.203	5.4	2.264
[6,6]-^{Ad}Sc_03	5.0	2.189	7.3	2.188	4.5	2.245
[6,6]-^{Ad}Sc_04	5.8	2.190	8.7	2.193	4.5	2.250
[6,6]-^{Ad}Sc_05	6.7	2.185	7.9	2.188	8.1	2.248
[6,6]-^{Ad}Sc_06	9.3	2.188	10.3	2.191	8.6	2.252
[6,6]-^{Ad}Sc_07	9.7	2.185	14.5	2.192	12.8	2.255
[6,6]-^{Ad}Sc_08	11.3	2.179	16.1	2.184	12.9	2.252
[6,6]-^{Ad}Sc_09	13.4	2.182	15.3	2.183	18.5	2.255
**[6,6]-^{Ad}M_01**	**1.4**	**2.209**	**0.0**	**2.211**	**1.1**	**2.272**
[6,6]-^{Ad}M_02	5.0	2.202	4.6	2.203	6.9	2.266
[6,6]-^{Ad}M_03	6.1	2.211	3.7	2.209	3.7	2.268
[6,6]-^{Ad}M_04	6.5	2.207	4.0	2.209	7.0	2.272
[6,6]-^{Ad}M_05	7.5	2.214	4.8	2.214	4.9	2.273
[6,6]-^{Ad}M_06	8.3	2.210	9.7	2.213	9.5	2.272
[6,6]-^{Ad}M_07	9.5	2.214	11.3	2.214	7.5	2.271

The values for M = Y are computed at the PBE/TZ2P level using Priroda code, values for M = Dy and Nd are computed at the PBE/PAW level using VASP.

Structures highlighted in red were used for the more detailed analysis of the LF splitting and coordination geometry (Figure 8 in the main text, and Tables S5 and S6 in SI)

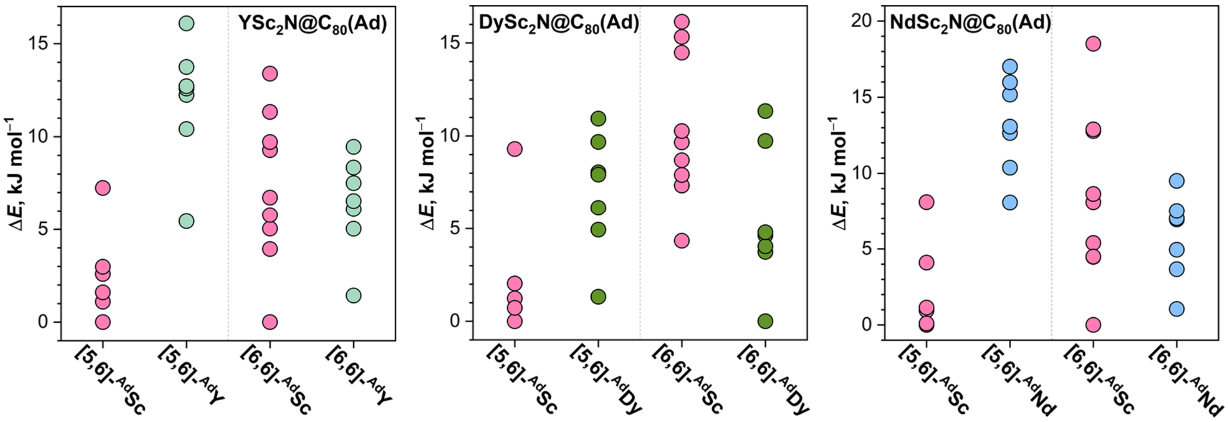

Figure S7a. DFT-calculated relative energies of $MSc_2N@C_{80}$(Ad) conformers (M = Y, Dy, Nd). The values for M = Y are computed at the PBE/TZ2P level using Priroda code, values for M = Dy and Nd are computed at the PBE/PAW level using VASP. There is a certain variation of relative energies for different metals, but the main results, i.e. the higher stability of ^{Ad}Sc conformers for [5,6] isomer and similar stability of ^{Ad}Sc and ^{Ad}M conformers for [6,6] isomers are reproduced for all metals.

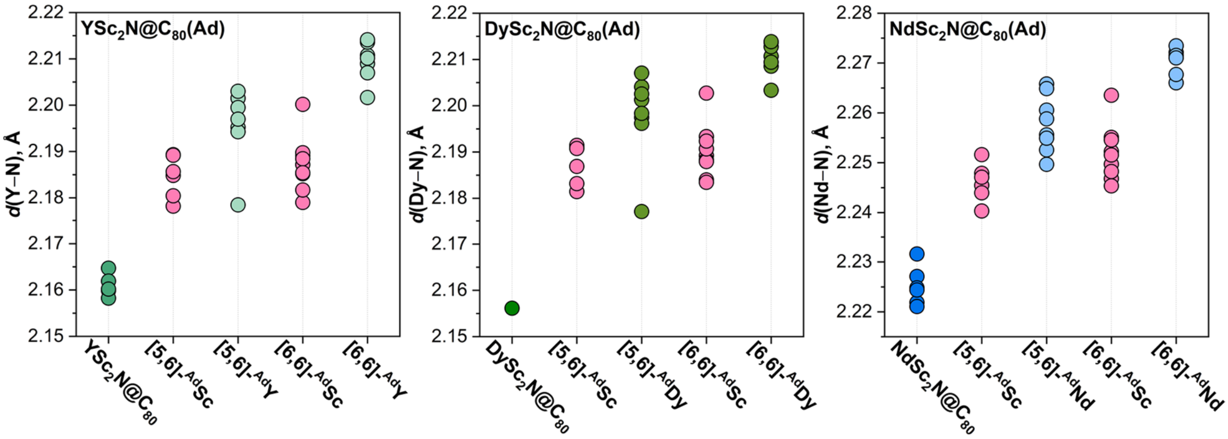

Figure S7b. DFT-optimized M–N bond lengths in conformers of $MSc_2N@C_{80}$ and $MSc_2N@C_{80}$(Ad) (M = Y, Dy, Nd). $MSc_2N@C_{80}$ has the shortest M–N bonds in the whole set. Thus, addition of carbene with opening of a C–C bond increases the space for the endohedral cluster and allows elongation of the M–N bond. For $MSc_2N@C_{80}$(Ad), ^{Ad}Sc conformers systematically show shorter M–N bonds than ^{Ad}M conformers, and [5,6] isomers have somewhat shorter M–N bonds than [6,6] isomers.

Photoluminescence spectra and decays of $NdSc_2N@C_{80}(Ad)$

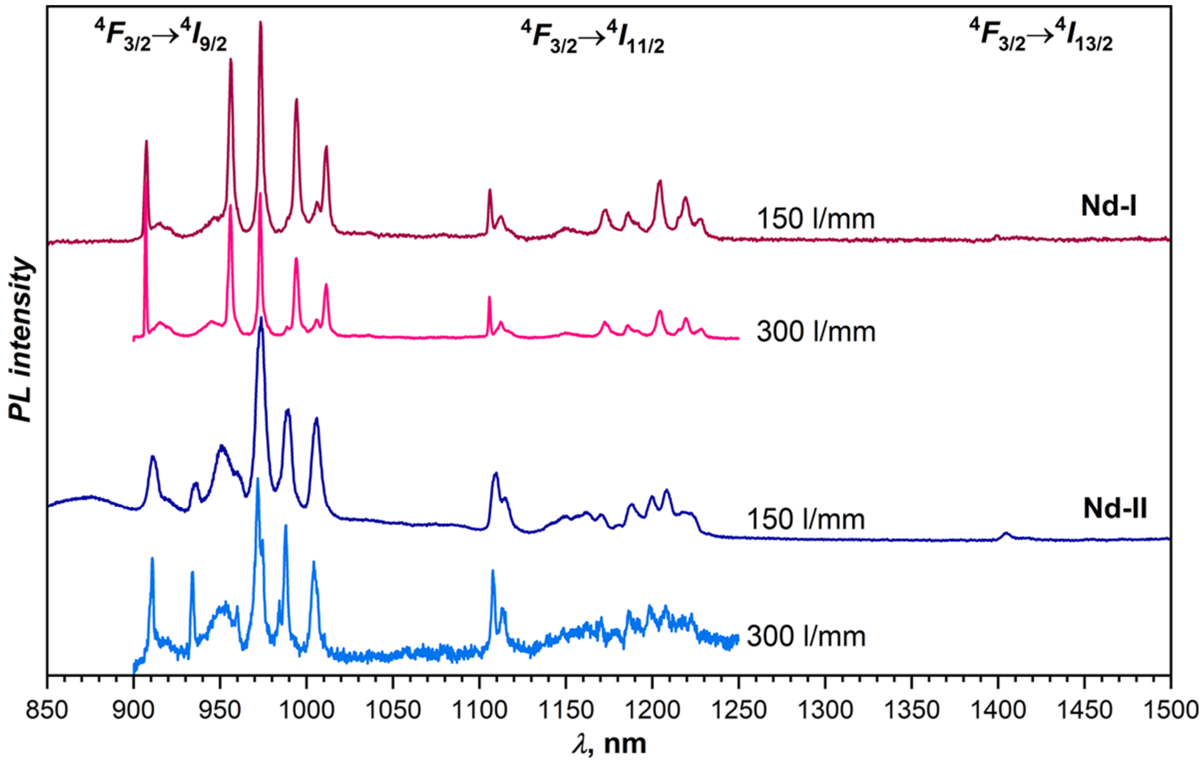

Figure S8. PL spectra of **Nd-I** and **Nd-II** measured at 5 K with 150 l/mm (lower-resolution) and 300 l/mm (higher-resolution) gratings.

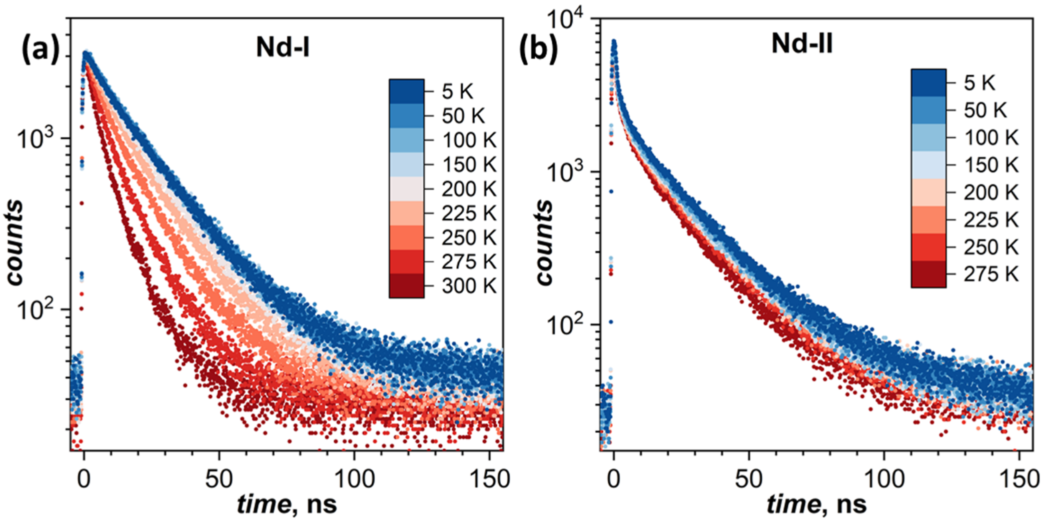

Figure S9. PL decays of (a) **Nd-I** and (b) **Nd-II** measured at different temperatures. Note that **Nd-I** exhibits more pronounced variation of decay rate with temperature than **Nd-II**. Decay data for each temperature and their fitting are shown in Figures S10 and S11.

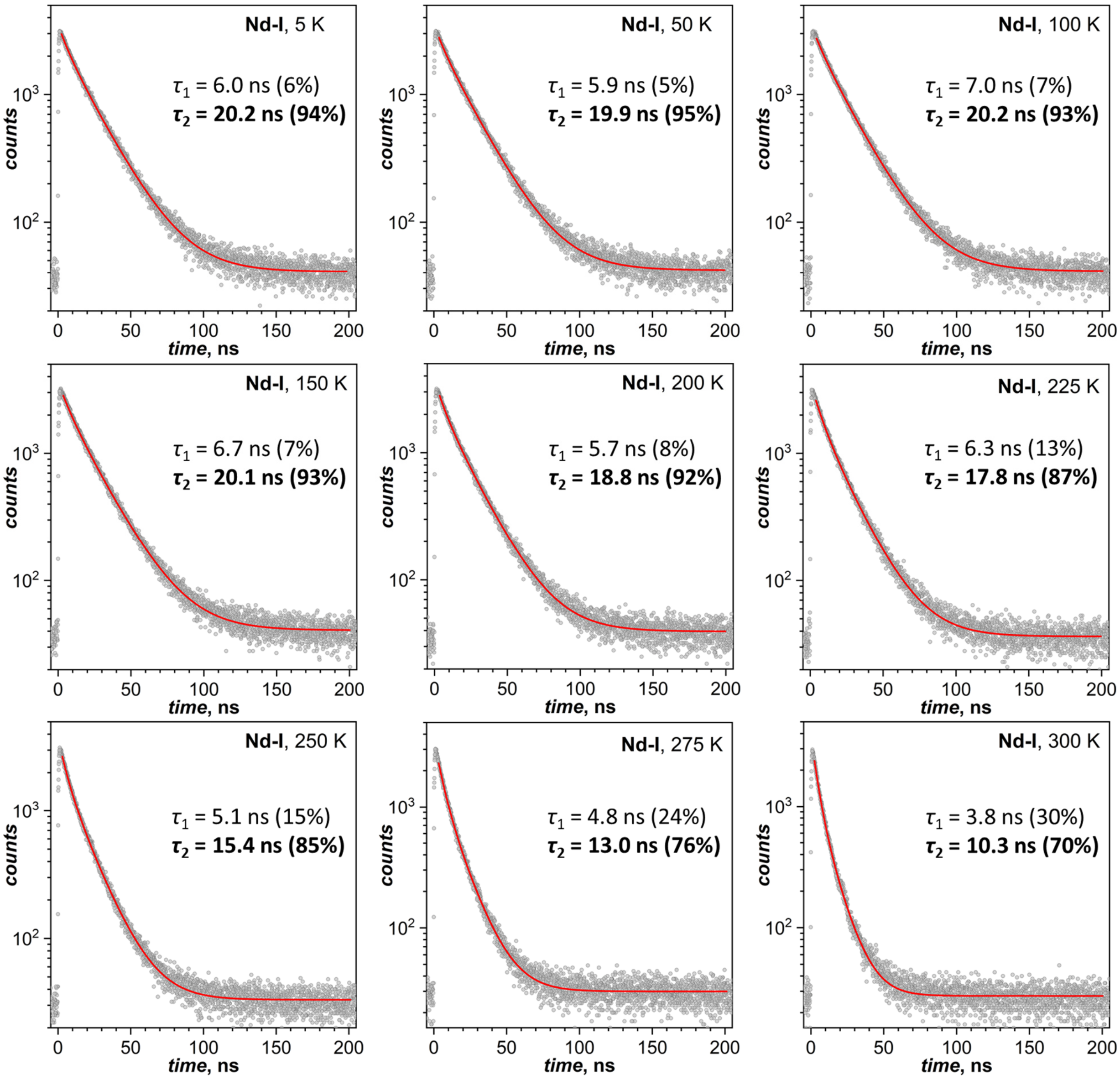

Figure S10. Luminescence decay curves of **Nd-I**, measured at different temperatures between 5 K and 300 K; laser excitation at 488 nm. Red curves are bi-exponential fits of experimental datapoints, fitted values of τ_1 and τ_1 are listed for each temperature, the main component is given in bold.

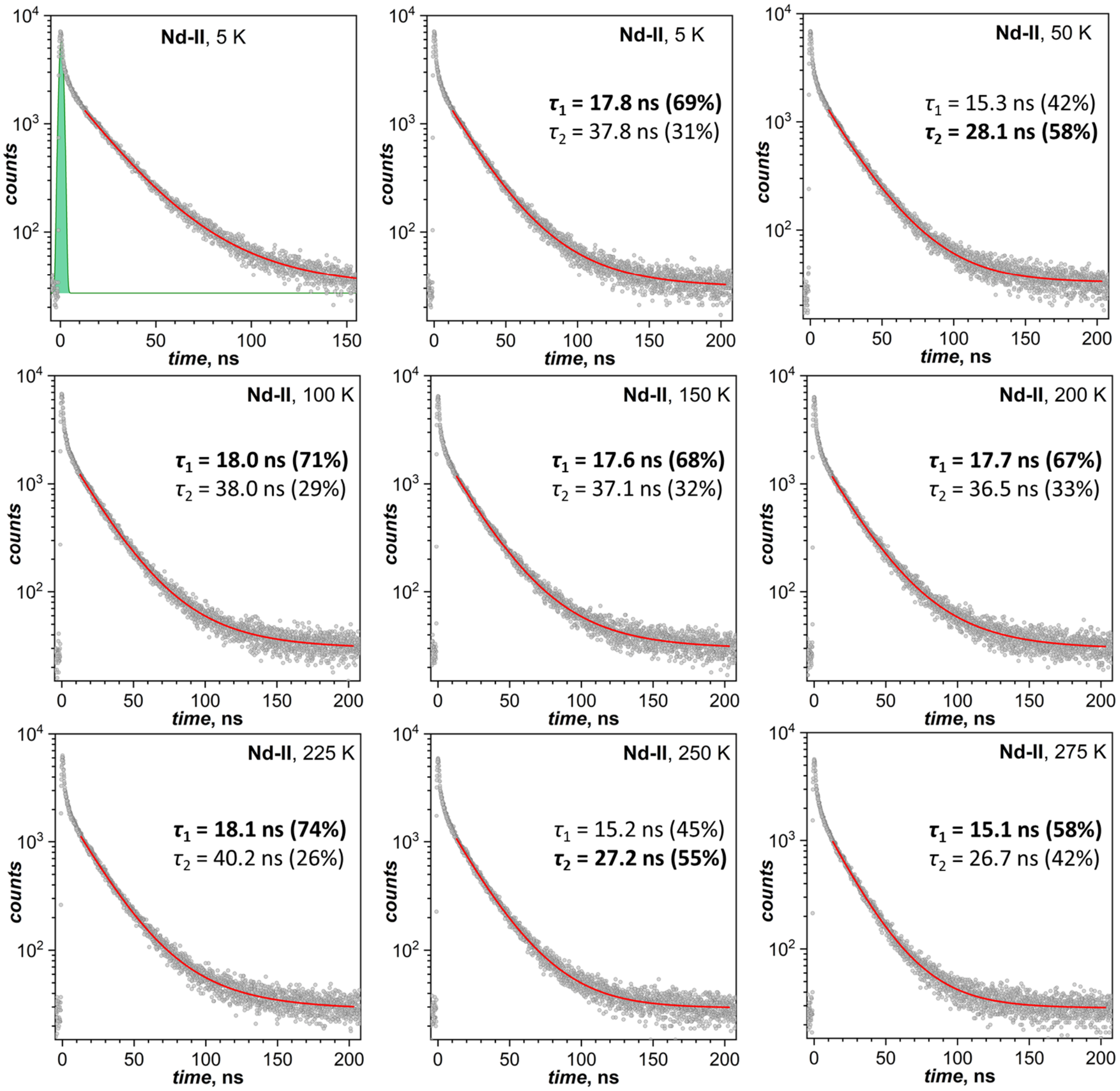

Figure S11. Luminescence decay curves of **Nd-II**, measured at different temperatures between 5 K and 275 K; laser excitation at 488 nm. Red curves are bi-exponential fits of experimental datapoints, fitted values of τ_1 and τ_1 are listed for each temperature, the main component is given in bold. The left top graph shows PL decay of **Nd-II** at 5 K compared to the approximate shape of the laser pulse: The sharp feature is assigned to the stray light from the laser pulse, which affects the shape of the decay curve and results of the fitting at short times. To avoid this influence, fitting of decay curves of **Nd-II** is performed starting from 10 ns after the pulse.

CASSCF/RASSI-SO calculations of ligand-field splitting in isomers and conformers of $NdSc_2N@C_{80}$(Ad)

For DFT-optimized structures of $MSc_2N@C_{80}$(Ad) conformers (Figure S5, Table S2), we performed CASSCF/RASSI-SO calculations of ligand-field splitting for the lanthanide ion. For each analyzed $MSc_2N@C_{80}$(Ad) conformer, Tables S3 and S4 list energies of LF states in the ground-state multipelt; contribution of axial LF terms (B_0^2, B_0^4, B_0^6) to the ligand-field splitting; net contributions of B_q^k terms for k = 2, 4, and 6 to the ligand-field splitting; principal values of the pseudospin g-tensor for the ground-state Kramers doublet (KD1), and the angle between g_z axis of KD1 and the M–N axis. For selected conformers, Tables S5 and S6 list compositions of KDs in the mJ basis and g-tensors for each Kramers doublet.

Table S3a. Energies of Kramers doublets (KDs), contribution of B_q^k terms to LF splitting, and pseudospin g-tensor of KD1 in conformers of [Ad]Sc-[5,6]-open $NdSc_2N@C_{80}$(Ad)

	Sc56_01	**Sc56_02**	Sc56_03	Sc56_04
KD1, cm^{-1}	0	**0**	0	0
KD2, cm^{-1}	318	**471**	310	478
KD3, cm^{-1}	549	**633**	533	632
KD4, cm^{-1}	685	**807**	704	776
KD5, cm^{-1}	821	**971**	820	949
B_0^2, %	19	**17**	16	17
B_0^4, %	4	**5**	4	4
B_0^6, %	8	**9**	7	8
$B_0^2 + B_0^4 + B_0^6$, %	30	**31**	26	30
$\sum_{\vert q\vert\leq 2} B_q^2$, %	45	**42**	45	42
$\sum_{\vert q\vert\leq 4} B_q^4$, %	10	**13**	11	13
$\sum_{\vert q\vert\leq 6} B_q^6$, %	40	**41**	40	41
g_x (KD1)	0.054	**0.039**	0.076	0.033
g_y (KD1)	0.069	**0.041**	0.084	0.039
g_z (KD1)	5.876	**5.721**	5.876	5.747
N–Nd/g_z (KD1),°	12.9	**2.7**	11.8	3.8

Table S3b. Energies of Kramers doublets (KDs), contribution of B_q^k terms to LF splitting, and pseudospin g-tensor of KD1 in conformers of ^{Ad}Nd-[5,6]-open $NdSc_2N@C_{80}(Ad)$

	M56_01	M56_02	M56_03	M56_04	M56_05	M56_07
KD1, cm^{-1}	0	0	0	0	0	0
KD2, cm^{-1}	298	269	288	296	279	305
KD3, cm^{-1}	453	424	454	455	445	472
KD4, cm^{-1}	613	661	691	654	676	659
KD5, cm^{-1}	752	772	804	776	790	794
B_0^2, %	15	13	13	14	14	14
B_0^4, %	3	2	3	3	3	3
B_0^6, %	6	6	7	6	5	8
$B_0^2 + B_0^4 + B_0^6$, %	23	21	23	23	21	24
$\sum_{\|q\|\leq 2} B_q^2$, %	42	41	40	41	40	40
$\sum_{\|q\|\leq 4} B_q^4$, %	8	8	9	9	8	10
$\sum_{\|q\|\leq 6} B_q^6$, %	45	45	45	44	45	45
g_x (KD1)	0.025	0.047	0.063	0.038	0.013	0.049
g_y (KD1)	0.058	0.074	0.090	0.075	0.050	0.074
g_z (KD1)	5.681	5.609	5.562	5.650	5.597	5.624
N–Nd/g_z (KD1),°	5.0	6.4	4.1	2.6	1.9	4.7

Table S3c. Energies of Kramers doublets (KDs), contribution of B_q^k terms to LF splitting, and pseudospin g-tensor of KD1 in conformers of ^{Ad}Sc-[6,6]-open $NdSc_2N@C_{80}(Ad)$

	**Sc66_01**	Sc66_03	Sc66_04	Sc66_05	Sc66_06
KD1, cm^{-1}	**0**	0	0	0	0
KD2, cm^{-1}	**405**	254	442	493	455
KD3, cm^{-1}	**591**	505	622	627	596
KD4, cm^{-1}	**730**	675	734	782	788
KD5, cm^{-1}	**886**	779	913	950	932
B_0^2, %	**16**	15	17	20	18
B_0^4, %	**4**	3	5	5	5
B_0^6, %	**8**	5	9	8	8
$B_0^2 + B_0^4 + B_0^6$, %	**28**	24	31	33	31
$\sum_{\vert q\vert \le 2} B_q^2$, %	**44**	47	43	43	43
$\sum_{\vert q\vert \le 4} B_q^4$, %	**12**	11	12	13	13
$\sum_{\vert q\vert \le 6} B_q^6$, %	**41**	38	41	41	41
g_x (KD1)	**0.051**	0.078	0.057	0.014	0.036
g_y (KD1)	**0.063**	0.095	0.058	0.021	0.043
g_z (KD1)	**5.782**	5.991	5.772	5.777	5.774
N–Nd/g_z (KD1),°	**9.2**	12.8	5.7	1.6	5.1

Table S3d. Energies of Kramers doublets (KDs), contribution of B_q^k terms to LF splitting, and pseudospin g-tensor of KD1 in conformers of ^{Ad}Nd-[6,6]-open $NdSc_2N@C_{80}$(Ad)

	**M66_01**	M66_02	M66_03	M66_04	M66_05	M66_06
KD1, cm^{-1}	**0**	0	0	0	0	0
KD2, cm^{-1}	**275**	259	287	328	263	240
KD3, cm^{-1}	**519**	508	512	537	514	507
KD4, cm^{-1}	**599**	595	595	634	614	639
KD5, cm^{-1}	**743**	716	742	792	744	736
B_0^2, %	**14**	15	15	19	13	12
B_0^4, %	**3**	3	3	5	3	3
B_0^6, %	**8**	4	8	13	6	3
$B_0^2 + B_0^4 + B_0^6$, %	**25**	22	27	37	22	17
$\sum_{\|q\|\leq 2} B_q^2$, %	**39**	39	40	39	38	38
$\sum_{\|q\|\leq 4} B_q^4$, %	**10**	9	10	11	10	9
$\sum_{\|q\|\leq 6} B_q^6$, %	**48**	48	47	47	47	47
g_x (KD1)	**0.071**	0.051	0.074	0.090	0.082	0.091
g_y (KD1)	**0.128**	0.136	0.125	0.112	0.139	0.136
g_z (KD1)	**5.489**	5.440	5.543	5.561	5.451	5.369
N–Nd/g_z (KD1),°	**6.6**	3.7	7.5	7.5	6.9	2.9

CASSCF/RASSI-SO calculations of ligand-field splitting in isomers and conformers of $DySc_2N@C_{80}$(Ad)

Table S4a. Energies of Kramers doublets (KDs), contribution of B_q^k terms to LF splitting, and pseudospin g-tensor of KD1 in conformers of ^{Ad}Sc-[5,6]-open $DySc_2N@C_{80}$(Ad)

	Sc56_01	**Sc56_02**	Sc56_03	Sc56_04	Sc56_05	Sc56_06
KD1, cm^{-1}	0	**0**	0	0	0	0
KD2, cm^{-1}	411	**343**	409	354	427	396
KD3, cm^{-1}	724	**711**	725	707	735	710
KD4, cm^{-1}	971	**1008**	974	982	974	966
KD5, cm^{-1}	1137	**1175**	1120	1158	1116	1126
KD6, cm^{-1}	1230	**1227**	1219	1245	1228	1231
KD7, cm^{-1}	1320	**1298**	1308	1312	1320	1308
KD8, cm^{-1}	1381	**1430**	1422	1375	1447	1417
B_0^2, %	51	**46**	45	50	44	44
B_0^4, %	5	**4**	4	5	4	4
B_0^6, %	1	**4**	1	3	2	0
$B_0^2 + B_0^4 + B_0^6$, %	56	**54**	49	58	49	48
$\sum_{\|q\|\le 2} B_q^2$, %	79	**78**	80	78	80	79
$\sum_{\|q\|\le 4} B_q^4$, %	7	**8**	7	8	7	7
$\sum_{\|q\|\le 6} B_q^6$, %	11	**12**	11	12	11	11
g_x (KD1)	2.1E-05	**1.6E-04**	1.4E-04	5.0E-05	1.1E-04	8.1E-05
g_y (KD1)	3.1E-05	**1.9E-04**	2.0E-04	6.1E-05	1.6E-04	1.1E-04
g_z (KD1)	19.975	**19.924**	19.964	19.943	19.973	19.975
N–Dy/g_z (KD1),°	1.4	**3.7**	2.0	2.0	2.8	3.0

Table S4b. Energies of Kramers doublets (KDs), contribution of B_q^k terms to LF splitting, and pseudospin g-tensor of KD1 in [Ad]Dy conformers of [5,6]-open $DySc_2N@C_{80}(Ad)$

	M56_01	M56_02	M56_03	M56_04	M56_05	M56_06	M56_07
KD1, cm^{-1}	0	0	0	0	0	0	0
KD2, cm^{-1}	275	280	279	295	295	315	266
KD3, cm^{-1}	546	567	573	576	585	609	553
KD4, cm^{-1}	747	770	786	780	787	812	771
KD5, cm^{-1}	887	876	895	904	897	911	896
KD6, cm^{-1}	959	936	935	962	953	960	954
KD7, cm^{-1}	1015	1042	1050	1043	1063	1086	1033
KD8, cm^{-1}	1152	1258	1271	1225	1271	1326	1210
B_0^2, %	47	41	38	41	41	43	39
B_0^4, %	2	2	2	2	2	3	2
B_0^6, %	2	1	1	1	1	0	2
$B_0^2 + B_0^4 + B_0^6$, %	51	44	41	44	44	46	43
$\sum_{\lvert q\rvert \le 2} B_q^2$, %	81	80	79	80	80	80	79
$\sum_{\lvert q\rvert \le 4} B_q^4$, %	5	5	6	6	6	6	6
$\sum_{\lvert q\rvert \le 6} B_q^6$, %	12	13	13	12	12	12	13
g_x (KD1)	3.3E-04	9.0E-04	1.3E-03	6.0E-04	8.5E-04	1.0E-03	4.6E-04
g_y (KD1)	4.2E-04	1.3E-03	1.8E-03	7.9E-04	1.1E-03	1.4E-03	5.3E-04
g_z (KD1)	19.853	19.778	19.782	19.848	19.800	19.801	19.823
N–Dy/g_z (KD1),°	0.9	1.4	5.9	6.2	6.4	7.8	4.0

Table S4c. Energies of Kramers doublets (KDs), contribution of B_q^k terms to LF splitting, and pseudospin g-tensor of KD1 in conformers of ^{Ad}Sc-[6,6]-open $DySc_2N@C_{80}$(Ad)

	**Sc66_01**	Sc66_02	Sc66_03	Sc66_04	Sc66_05	Sc66_06
KD1, cm^{-1}	**0**	0	0	0	0	0
KD2, cm^{-1}	**367**	445	460	347	363	369
KD3, cm^{-1}	**707**	738	756	689	718	724
KD4, cm^{-1}	**980**	966	974	969	990	993
KD5, cm^{-1}	**1158**	1122	1111	1137	1179	1160
KD6, cm^{-1}	**1227**	1235	1236	1231	1267	1227
KD7, cm^{-1}	**1307**	1324	1341	1295	1331	1313
KD8, cm^{-1}	**1373**	1406	1442	1369	1376	1424
B_0^2, %	**47**	49	50	46	54	48
B_0^4, %	**4**	4	4	4	5	4
B_0^6, %	**2**	2	3	3	4	2
$B_0^2 + B_0^4 + B_0^6$, %	**53**	56	57	53	63	55
$\sum_{\|q\|\leq 2} B_q^2$, %	**79**	80	81	79	79	79
$\sum_{\|q\|\leq 4} B_q^4$, %	**7**	7	7	7	8	7
$\sum_{\|q\|\leq 6} B_q^6$, %	**11**	11	10	12	11	11
g_x (KD1)	**5.3E-05**	4.0E-05	2.1E-04	2.4E-05	5.8E-05	4.7E-05
g_y (KD1)	**6.7E-05**	4.5E-05	2.8E-04	3.9E-05	6.5E-05	6.0E-05
g_z (KD1)	**19.955**	19.984	19.982	19.959	19.949	19.931
N–Dy/g_z (KD1),°	1.2	1.6	2.5	2.1	2.6	0.7

Table S4d. Energies of Kramers doublets (KDs), contribution of B_q^k terms to LF splitting, and pseudospin g-tensor of KD1 in conformers of ^{Ad}Dy-[6,6]-open $DySc_2N@C_{80}$(Ad)

	**M66_01**	M66_02	M66_03	M66_04	M66_06	M66_07
KD1, cm^{-1}	**0**	0	0	0	0	0
KD2, cm^{-1}	**273**	294	270	253	311	291
KD3, cm^{-1}	**531**	540	528	530	578	556
KD4, cm^{-1}	**742**	733	745	761	774	772
KD5, cm^{-1}	**868**	867	883	897	898	902
KD6, cm^{-1}	**947**	955	959	965	951	960
KD7, cm^{-1}	**1018**	1034	1041	1053	1027	1009
KD8, cm^{-1}	**1119**	1116	1086	1066	1230	1185
B_0^2, %	**43**	47	45	52	41	40
B_0^4, %	**3**	3	3	4	3	3
B_0^6, %	**1**	1	2	4	0	1
$B_0^2 + B_0^4 + B_0^6$, %	**47**	51	49	59	44	43
$\sum_{\|q\|\leq 2} B_q^2$, %	**78**	79	79	77	78	78
$\sum_{\|q\|\leq 4} B_q^4$, %	**5**	5	5	7	5	6
$\sum_{\|q\|\leq 6} B_q^6$, %	**14**	14	14	14	14	14
g_x (KD1)	**1.5E-03**	1.6E-03	1.2E-03	1.3E-03	3.0E-03	2.0E-03
g_y (KD1)	**1.9E-03**	1.8E-03	1.3E-03	1.5E-03	3.7E-03	2.5E-03
g_z (KD1)	**19.921**	19.926	19.942	19.934	19.870	19.913
N–Dy/g_z (KD1),°	**0.8**	1.7	1.8	1.3	2.2	3.2

Ab initio calculated ligand-field splitting, pseudospin g-tensors, and state compositions for selected conformers

Table S5a. Ligand-field splitting of Nd^{3+}-$^4I_{9/2}$ multiplet in [5,6]-$NdSc_2N@C_{80}$(Ad) (conformer **[5,6]-^{Ad}Sc_02**) and weights of m_J states in KDs computed at the CASSCF/RASSI level.

KD	exp. _E_ cm^{-1}	calc. _E_ cm^{-1}	|±9/2⟩ %	|±7/2⟩	|±5/2⟩	|±3/2⟩	|±1/2⟩	g_x	g_y	g_z
1	0	0	**87**	1	3	9	1	0.039	0.041	5.721
2	565	471	1	**14**	**65**	7	**13**	2.046	0.557	2.735
3	751	633	10	**34**	**15**	**40**	2	1.908	0.173	3.370
4	967	807	2	**44**	9	**38**	8	0.315	1.740	3.915
5	1138	971	0	8	9	6	**77**	3.873	3.009	0.489

Table S5b. Ligand-field splitting of Nd^{3+}-$^4I_{9/2}$ multiplet in [6,6]-$NdSc_2N@C_{80}$(Ad) (conformer **[6,6]-^{Ad}Sc_01**) and weights of m_J states in KDs computed at the CASSCF/RASSI level.

KD	exp. _E_ cm^{-1}	calc. _E_ cm^{-1}	|±9/2⟩ %	|±7/2⟩	|±5/2⟩	|±3/2⟩	|±1/2⟩	g_x	g_y	g_z
1	0	0	**87**	0	7	5	2	0.051	0.063	5.782
2	~450	405	2	**31**	**42**	**18**	7	0.227	1.037	4.625
3	689	591	9	**18**	**37**	**29**	7	4.567	0.991	0.091
4	857	730	2	**44**	6	**34**	**14**	2.740	0.390	3.459
5	1021	886	0	6	9	**14**	**71**	4.479	2.294	0.261

Table S5c. Ligand-field splitting of Nd^{3+}-$^4I_{9/2}$ multiplet in [6,6]-$NdSc_2N@C_{80}$(Ad) (conformer **[6,6]-^{Ad}Nd_01**) and weights of m_J states in KDs computed at the CASSCF/RASSI level.

KD	exp. _E_ cm^{-1}	calc. _E_ cm^{-1}	|±9/2⟩ %	|±7/2⟩	|±5/2⟩	|±3/2⟩	|±1/2⟩	g_x	g_y	g_z
1	0	0	**84**	0	2	8	6	0.071	0.128	5.489
2	285	275	1	**23**	**57**	**13**	6	2.934	0.147	2.156
3	575	519	8	**30**	13	**44**	5	2.157	1.625	0.252
4	730	599	5	**45**	**15**	**26**	9	0.553	3.381	1.911
5	832	743	2	2	**12**	9	**75**	3.957	2.902	0.230

* origin is at −13 cm^{-1}.

Exp. values are from PL measurements; assignment of PL features to individual KDs for **Nd-II** is more ambiguous than for **Nd-I**.

Table S6a. Ligand-field splitting of Dy^{3+}-$^6H_{15/2}$ multiplet in [5,6]-$DySc_2N@C_{80}$(Ad) (conformer **[5,6]-^{Ad}Sc_02**) and weights of m_J states in KDs computed at the CASSCF/RASSI level.

KD	*E* cm^{-1}	|±15/2⟩ %	|±13/2⟩	|±11/2⟩	|±9/2⟩	|±7/2⟩	|±5/2⟩	|±3/2⟩	|±1/2⟩	g_x	g_y	g_z
1	0	**99**	0	1	0	0	0	0	0	$1.6\cdot10^{-4}$	$1.9\cdot10^{-4}$	19.924
2	343	0	**99**	0	1	0	0	0	0	$3.0\cdot10^{-3}$	$3.1\cdot10^{-3}$	17.103
3	711	1	0	**97**	2	0	0	0	0	0.040	0.046	14.336
4	1008	0	0	1	**94**	3	1	1	0	0.139	0.222	11.564
5	1175	0	0	0	3	**62**	8	8	**19**	1.377	3.924	8.511
6	1227	0	0	0	1	**25**	**27**	**19**	**28**	8.303	7.872	2.202
7	1298	0	0	0	0	8	**51**	**38**	2	14.830	1.766	0.825
8	1430	0	0	0	0	2	**14**	**33**	**50**	19.139	0.413	0.075

Table S6b. Ligand-field splitting of Dy^{3+}-$^6H_{15/2}$ multiplet in [6,6]-$DySc_2N@C_{80}$(Ad) (conformer **[6,6]-^{Ad}Dy_01**) and weights of m_J states in KDs computed at the CASSCF/RASSI level.

KD	*E* cm^{-1}	|±15/2⟩ %	|±13/2⟩	|±11/2⟩	|±9/2⟩	|±7/2⟩	|±5/2⟩	|±3/2⟩	|±1/2⟩	g_x	g_y	g_z
1	0	**100**	0	0	0	0	0	0	0	$5.3\cdot10^{-5}$	$6.7\cdot10^{-5}$	19.956
2	367	0	**97**	2	0	0	0	0	0	$2.7\cdot10^{-3}$	$2.9\cdot10^{-3}$	17.193
3	707	0	3	**91**	6	0	0	0	0	0.048	0.055	14.340
4	980	0	0	6	**87**	6	1	0	0	0.164	0.222	11.604
5	1158	0	0	1	6	**89**	1	2	2	0.662	0.959	9.508
6	1227	0	0	0	1	2	**60**	**23**	**15**	5.466	12.969	2.726
7	1307	0	0	0	0	1	**35**	**38**	**26**	2.733	12.228	0.296
8	1373	0	0	0	1	2	3	**37**	**57**	3.251	16.850	0.677

Table S6c. Ligand-field splitting of Dy^{3+}-$^6H_{15/2}$ multiplet in [6,6]-$DySc_2N@C_{80}$(Ad) (conformer **[6,6]-^{Ad}Dy_01**) and weights of m_J states in KDs computed at the CASSCF/RASSI level. (**Dy66_01**)

KD	*E* cm^{-1}	|±15/2⟩ %	|±13/2⟩	|±11/2⟩	|±9/2⟩	|±7/2⟩	|±5/2⟩	|±3/2⟩	|±1/2⟩	g_x	g_y	g_z
1	0	**99**	0	1	0	0	0	0	0	$1.5\cdot10^{-3}$	$1.9\cdot10^{-3}$	19.921
2	273	0	**98**	1	0	0	1	0	0	0.017	0.019	17.158
3	531	0	1	**93**	4	0	0	1	0	0.153	0.182	14.351
4	742	0	0	4	**91**	2	2	1	0	0.568	1.012	11.669
5	868	0	0	0	2	**72**	9	10	7	4.872	8.326	6.492
6	947	0	0	1	1	**20**	**44**	**16**	**18**	3.754	11.580	0.592
7	1018	0	0	0	1	4	**33**	**36**	**26**	18.017	1.352	0.895
8	1119	0	0	1	1	1	**12**	**36**	**50**	19.107	0.030	0.124

Magneto-structural correlations

Having computed LF splitting in a series of conformers of $NdSc_2N@C_{80}(Ad)$ and $DySc_2N@C_{80}(Ad)$ (Tables S3−S4), we then tried to find if certain correlations between their structural parameters and the LF splitting can be found. An obvious example of such correlation is the difference between LF properties of lanthanide ions coordinated to Ad pocket and to unfunctionalized fragment of the cage as discussed in the main text. Here we aimed to find if more subtle correlations can be established, such as between coordination geometries within the same type of coordination. One obvious parameter to consider is the length of the lanthanide−nitrogen bond. Figure S12 plots $\Delta_{1,2}$ and Δ_{LF} splitting versus DFT-computed Ln−N bond lengths. Although a loose correlation between the bond length and the splitting is present (shorter bonds should give stronger splitting), the fine difference between the splitting within each group of structures (^{Ad}Ln or ^{cage}Ln coordination types) cannot be described by this parameter alone.

We thus tried to parameterize Ln−C interactions. Here two parameters can be considered for each Ln−C contact: the N−Ln−C angle θ representing the deviation from axiality and the Ln−C distance R. The two parameters are combined into one term akin to equation for dipolar interactions, $(3\cos^2(\theta) - 1)/R^3$. As each lanthanide ion is coordinated to many carbons with interaction strengths gradually decreasing with the Ln−C distance, we introduced the sum of individual atomic parameters as a metric:

$$\Sigma_k = \sum_{i=1:k} \frac{3\cos^2(\theta_i) - 1}{R_i^3}$$

where summation is done over k carbon atoms nearest to the lanthanide ion. That is, Σ_1 only considers the nearest carbon, Σ_2 – two nearest carbons etc. Plots of $\Delta_{1,2}$ and Δ_{LF} splitting versus Σ_k with different number of *k* are shown in Figures S13-S18. Some of these metrics showed apparent correlations within one or another group of structures (e.g., Σ_1 for $\Delta_{1,2}$ of ^{cage}Dy), none of these metrics showed a universal correlation with ligand field splitting in the whole group. Given the presence of many lanthanide-carbon interactions in metallofullerenes, it is questionable that simple correlations with local coordination geometry can be established.

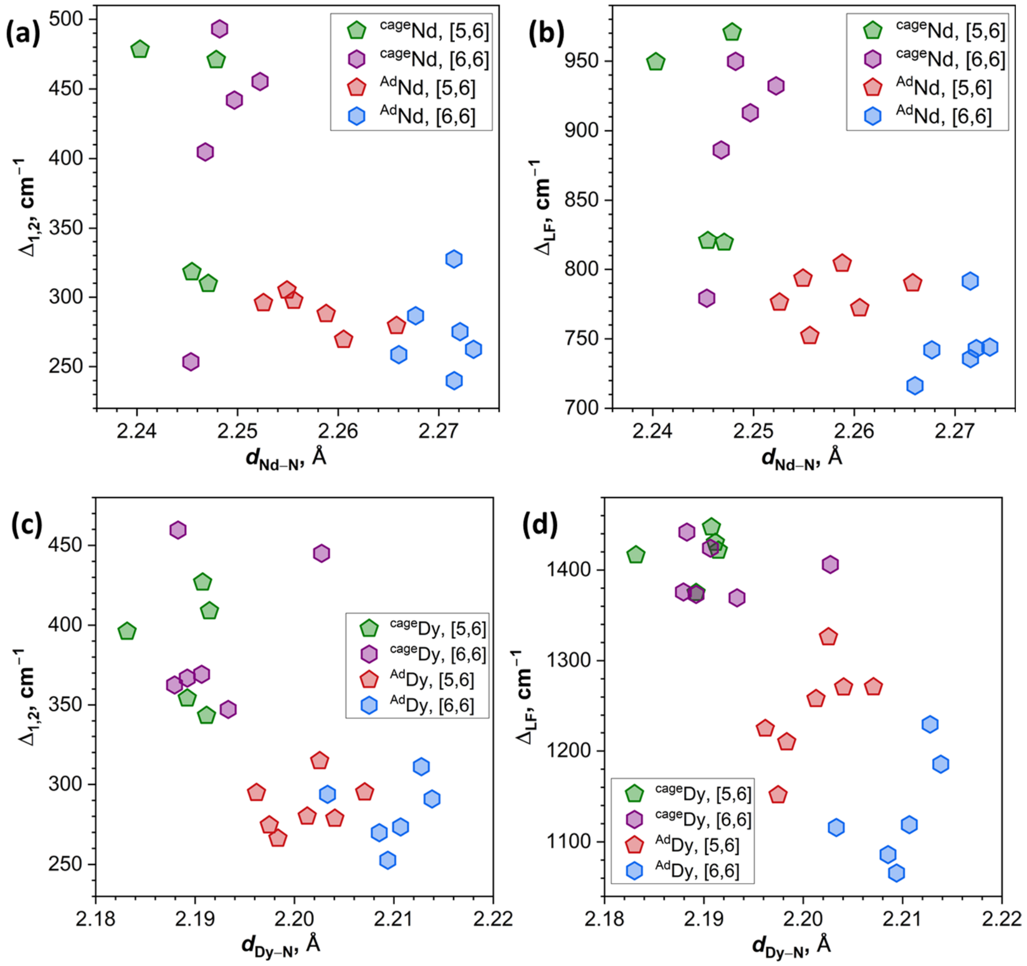

Figure S12. Correlations between DFT-computed Ln–N bond lengths and LF splitting ($\Delta_{1,2}$ and Δ_{LF}) in $NdSc_2N@C_{80}$(Ad) (a, b) and $DySc_2N@C_{80}$(Ad) (c, d). Four series of structures are denoted with different colors (two regioisomers and two types of Ln coordination for each isomer).

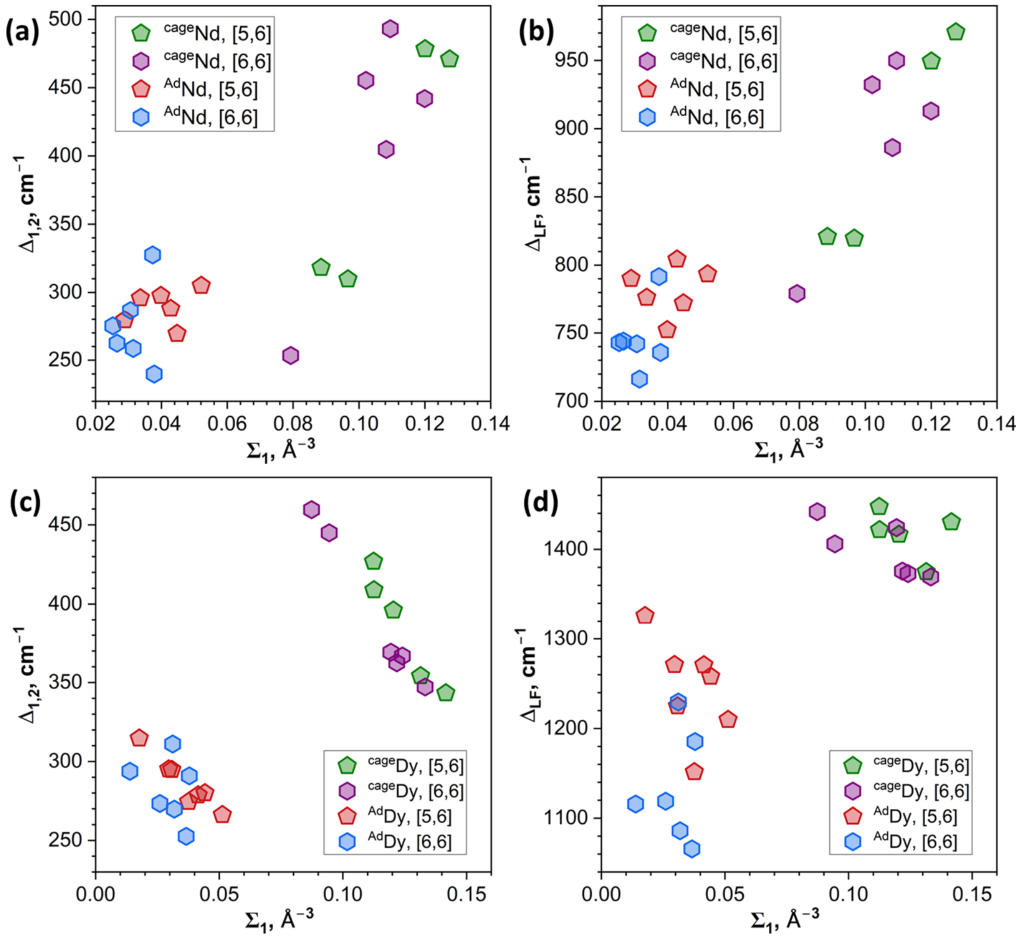

Figure S13. Correlations between Σ_1 and LF splitting ($\Delta_{1,2}$ and Δ_{LF}) in $NdSc_2N@C_{80}$(Ad) (a, b) and $DySc_2N@C_{80}$(Ad) (c, d). Four series of structures are denoted with different colors (two regioisomers and two types of Ln coordination for each isomer).

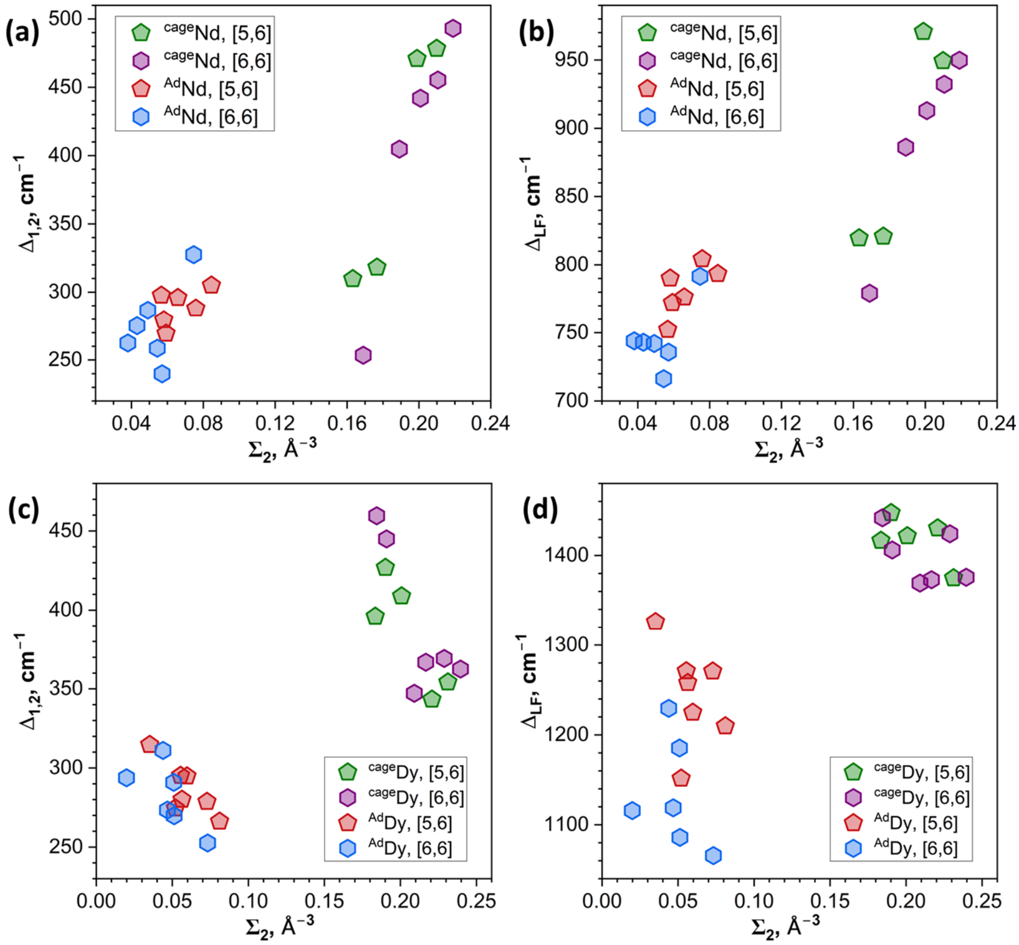

Figure S14. Correlations between Σ_2 and LF splitting ($\Delta_{1,2}$ and Δ_{LF}) in $NdSc_2N@C_{80}(Ad)$ (a, b) and $DySc_2N@C_{80}(Ad)$ (c, d). Four series of structures are denoted with different colors (two regioisomers and two types of Ln coordination for each isomer).

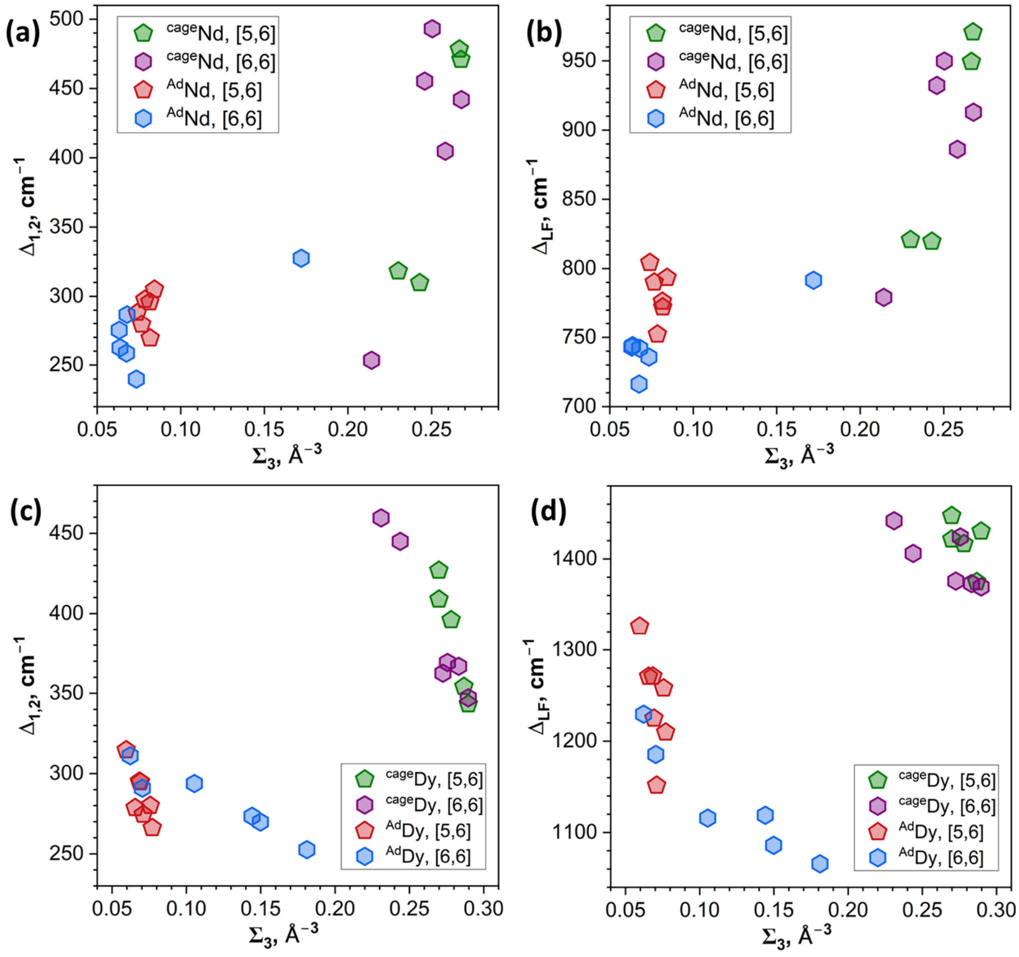

Figure S15. Correlations between Σ_3 and LF splitting ($\Delta_{1,2}$ and Δ_{LF}) in $NdSc_2N@C_{80}(Ad)$ (a, b) and $DySc_2N@C_{80}(Ad)$ (c, d). Four series of structures are denoted with different colors (two regioisomers and two types of Ln coordination for each isomer).

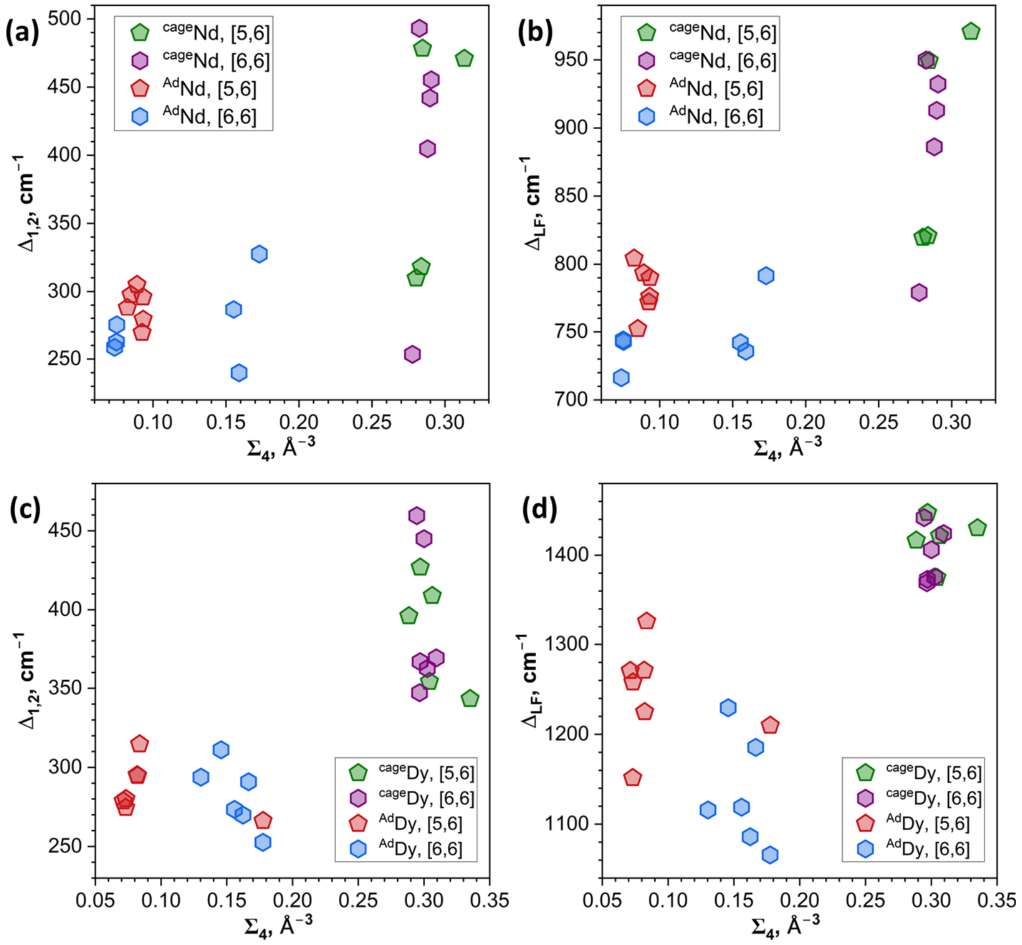

Figure S16. Correlations between Σ_4 and LF splitting ($\Delta_{1,2}$ and Δ_{LF}) in $NdSc_2N@C_{80}$(Ad) (a, b) and $DySc_2N@C_{80}$(Ad) (c, d). Four series of structures are denoted with different colors (two regioisomers and two types of Ln coordination for each isomer).

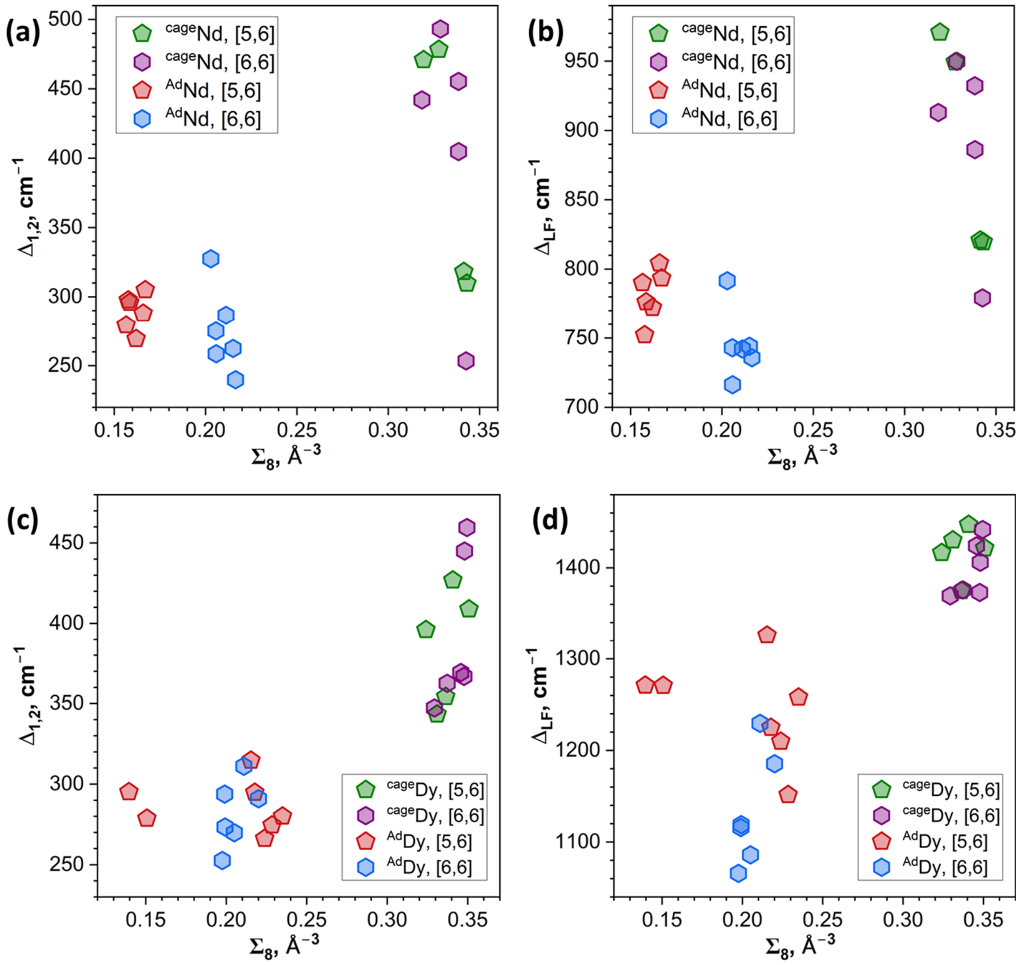

Figure S17. Correlations between Σ_8 and LF splitting ($\Delta_{1,2}$ and Δ_{LF}) in $NdSc_2N@C_{80}(Ad)$ (a, b) and $DySc_2N@C_{80}(Ad)$ (c, d). Four series of structures are denoted with different colors (two regioisomers and two types of Ln coordination for each isomer).

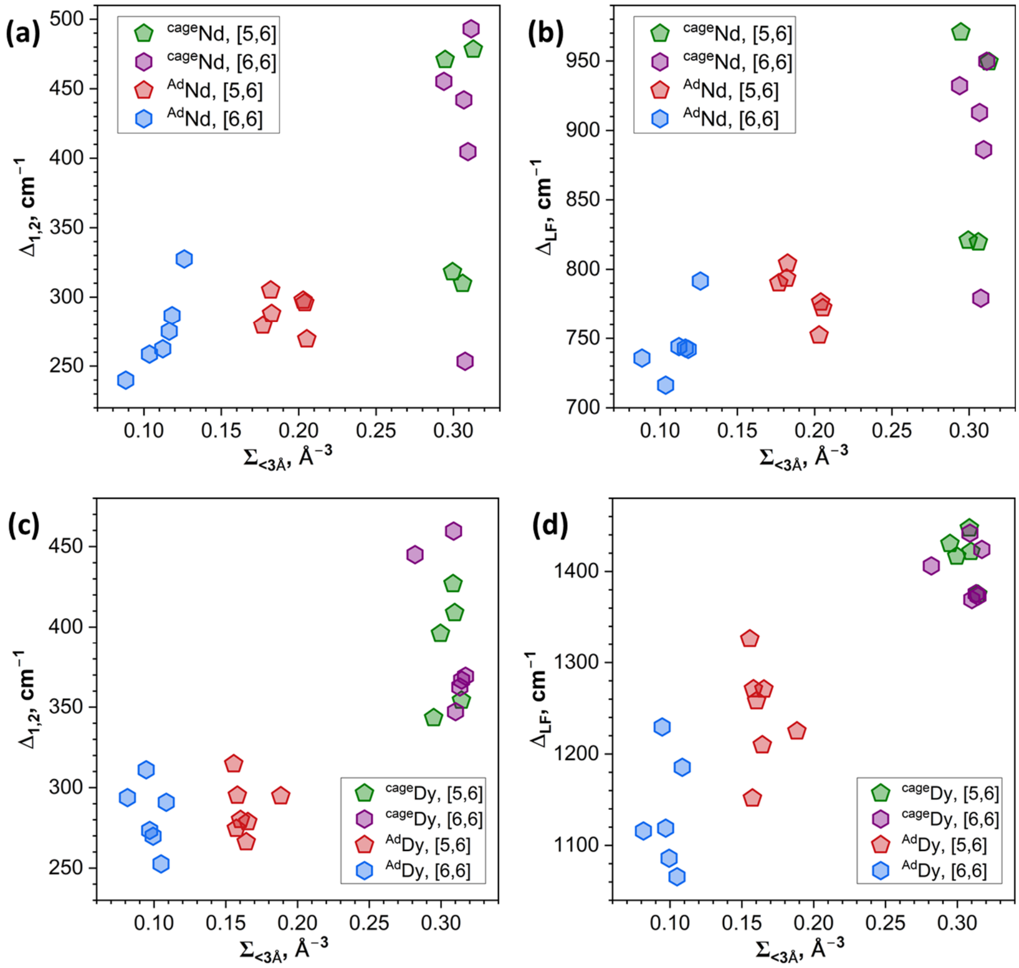

Figure S18. Correlations between $\Sigma_{<3Å}$ and LF splitting ($\Delta_{1,2}$ and Δ_{LF}) in $NdSc_2N@C_{80}(Ad)$ (a, b) and $DySc_2N@C_{80}(Ad)$ (c, d). Four series of structures are denoted with different colors (two regioisomers and two types of Ln coordination for each isomer).

Magnetization relaxation times of $DySc_2N@C_{80}$(Ad)

Magnetization decay curves were measured after the samples were first magnetized at 7 Tesla and then the field was quickly ramped to the required value. The decay curves were fitted with stretched exponential function:

$$M(t) = M_{eq} + (M_0 - M_{eq}) \exp\left[-\left(\frac{t}{\tau}\right)^{\beta}\right]$$

Where M_{eq} and M_0 are the equilibrium and initial magnetizations, respectively, τ is the relaxation time and β is a parameter, characterizing distribution of relaxation rates in the sample. For a single-exponential decay, β = 1. Reliable estimation of τ requires the duration of the decay curve measurement to be at least 3 τ. For relaxation times shorter than ~100 s, the method becomes increasingly unreliable because substantial part of the sample relaxes during the field sweep, and therefore only the tail with longer times is measured. Long times become overrepresented in the distribution, leading to overestimation of τ. Decay curves and their fitting are plotted in Figures S19–S20, whereas numerical values of τ and β are listed in Tables S7 and S8.

Table S7. Magnetization relaxation times and β parameters of **Dy-I** and **Dy-II** determined by DC magnetometry at different temperatures in the field of 0.2 T.

	**Dy-I**				**Dy-II**			
***T*, K**	***τ*, s**	**±Δ*τ*, s**	***β***	**±Δ*β***	***τ*, s**	**±Δ*τ*, s**	***β***	**±Δ*β***
2.7	11827.5	8.1	0.670	0.000				
3	6162.2	3.5	0.685	0.000				
3.25	3889.3	3.0	0.658	0.001				
3.5	2477.9	3.1	0.651	0.001				
3.75	1594.3	5.7	0.552	0.001	1559.5	2.5	0.562	0.001
4	1000.8	2.0	0.697	0.002	1007.8	2.1	0.598	0.002
4.25					707.2	2.4	0.605	0.002
4.5	490.3	1.6	0.720	0.003	467.9	1.8	0.668	0.003
4.75					371.7	2.0	0.625	0.004
5	278.8	1.5	0.705	0.005	279.8	1.7	0.651	0.005
5.25					239.5	1.9	0.608	0.005
5.5	178.5	1.2	0.691	0.005	196.7	1.7	0.612	0.006
6	143.4	1.1	0.718	0.006	169.4	1.4	0.566	0.005
6.5	118.3	1.0	0.681	0.007	140.2	1.3	0.611	0.005
7	102.5	0.8	0.668	0.005	123.8	0.7	0.635	0.004
7.5					104.5	0.9	0.662	0.006

Table S8. Magnetization relaxation times of **Dy-I** and **Dy-II** and β parameters determined by DC magnetometry at 4 K and in different magnetic fields.

	**Dy-I**				**Dy-II**			
**$\mu_0 H$, Oe**	***τ*, s**	**±Δ*τ*, s**	***β***	**±Δ*β***	***τ*, s**	**±Δ*τ*, s**	***β***	**±Δ*β***
250	53.6	0.6	0.759	0.006				
300	82.7	0.7	0.745	0.005				
350	133.7	0.5	0.759	0.004				
400	171.2	0.8	0.671	0.003	54.7	0.6	0.557	0.004
450	277.1	0.8	0.724	0.003	79.3	0.7	0.541	0.004
500	358.7	1.3	0.723	0.003	117.6	1.0	0.574	0.005
600	537.6	1.9	0.736	0.002	213.8	2.3	0.550	0.003
700	702.1	1.5	0.727	0.002	383.9	6.5	0.525	0.003
800	786.1	1.9	0.745	0.002	566.8	2.8	0.541	0.001
900	881.3	1.6	0.731	0.002	675.2	2.4	0.555	0.001
1000	893.0	1.6	0.723	0.001	748.7	1.3	0.579	0.001
1200	927.1	1.8	0.729	0.002	835.1	1.6	0.611	0.001
1400	940.5	1.6	0.725	0.001	873.9	1.6	0.631	0.001
1500	985.1	2.1	0.740	0.002	893.6	1.1	0.631	0.001
1600	961.8	1.9	0.724	0.002	904.1	1.5	0.639	0.001
1800	1001.7	1.7	0.685	0.002	955.7	1.6	0.626	0.001
1900	1002.9	1.9	0.693	0.002	957.7	1.6	0.627	0.001
2000	1000.8	2.0	0.697	0.002	1007.8	2.1	0.598	0.002
2100	1023.4	2.1	0.709	0.002	1008.7	2.1	0.598	0.002
2200	984.2	1.9	0.687	0.002	985.1	1.9	0.617	0.001
2400	975.6	2.0	0.697	0.002	985.7	2.0	0.621	0.002
2500	968.8	2.0	0.696	0.002	954.0	1.9	0.641	0.001
2600	929.1	1.9	0.717	0.002	948.9	1.9	0.637	0.001
2800	895.0	2.1	0.729	0.002	942.5	2.0	0.643	0.002
3000	903.7	2.2	0.739	0.002	930.1	2.0	0.639	0.002
3200	857.8	2.0	0.723	0.002	868.7	1.9	0.672	0.001
3400	840.3	2.0	0.720	0.002	832.5	2.1	0.688	0.002
3500	878.7	2.4	0.746	0.002	826.6	2.0	0.687	0.002
4000	733.2	2.4	0.778	0.002	791.5	2.1	0.684	0.002
4500	688.7	2.3	0.772	0.002	753.1	2.2	0.682	0.002
5000	616.5	2.0	0.741	0.003	661.7	2.5	0.708	0.002
5500	568.7	2.0	0.733	0.003	631.9	2.6	0.696	0.002
6000	549.5	2.1	0.735	0.003	597.6	2.6	0.687	0.003
7000	403.6	2.0	0.734	0.003	477.5	3.2	0.710	0.003
8000	388.3	2.5	0.734	0.004	435.7	3.3	0.691	0.004
9000	335.9	1.6	0.669	0.003	373.4	5.2	0.683	0.006
10000					336.1	3.8	0.690	0.005

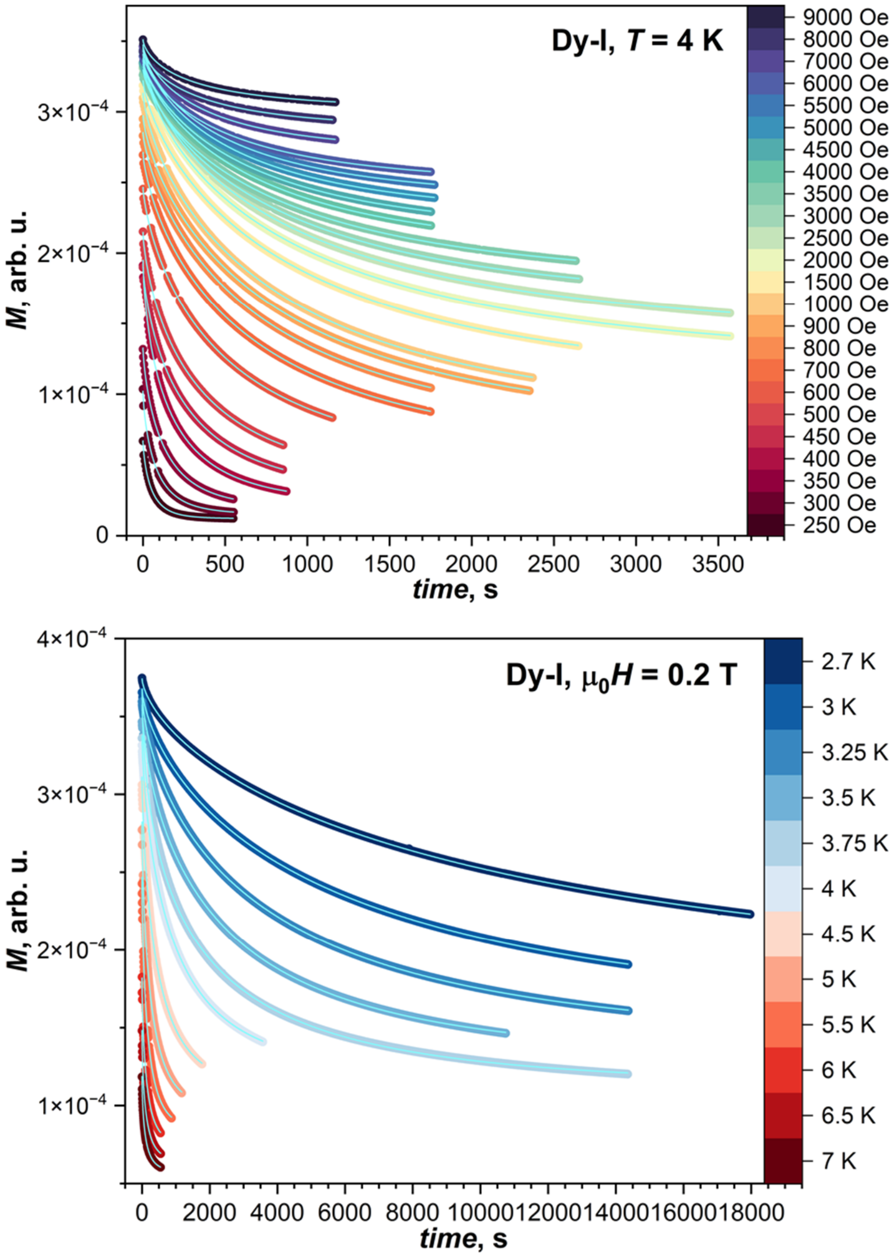

Figure S19. Magnetization decay curves (colored dots) and their fitting with stretched exponential function (cyan curves) for **Dy-I**. Top panel: temperature is fixed to 4 K, magnetic field is varied. Bottom panel: magnetic field is fixed to 0.2 T, temperature is varied.

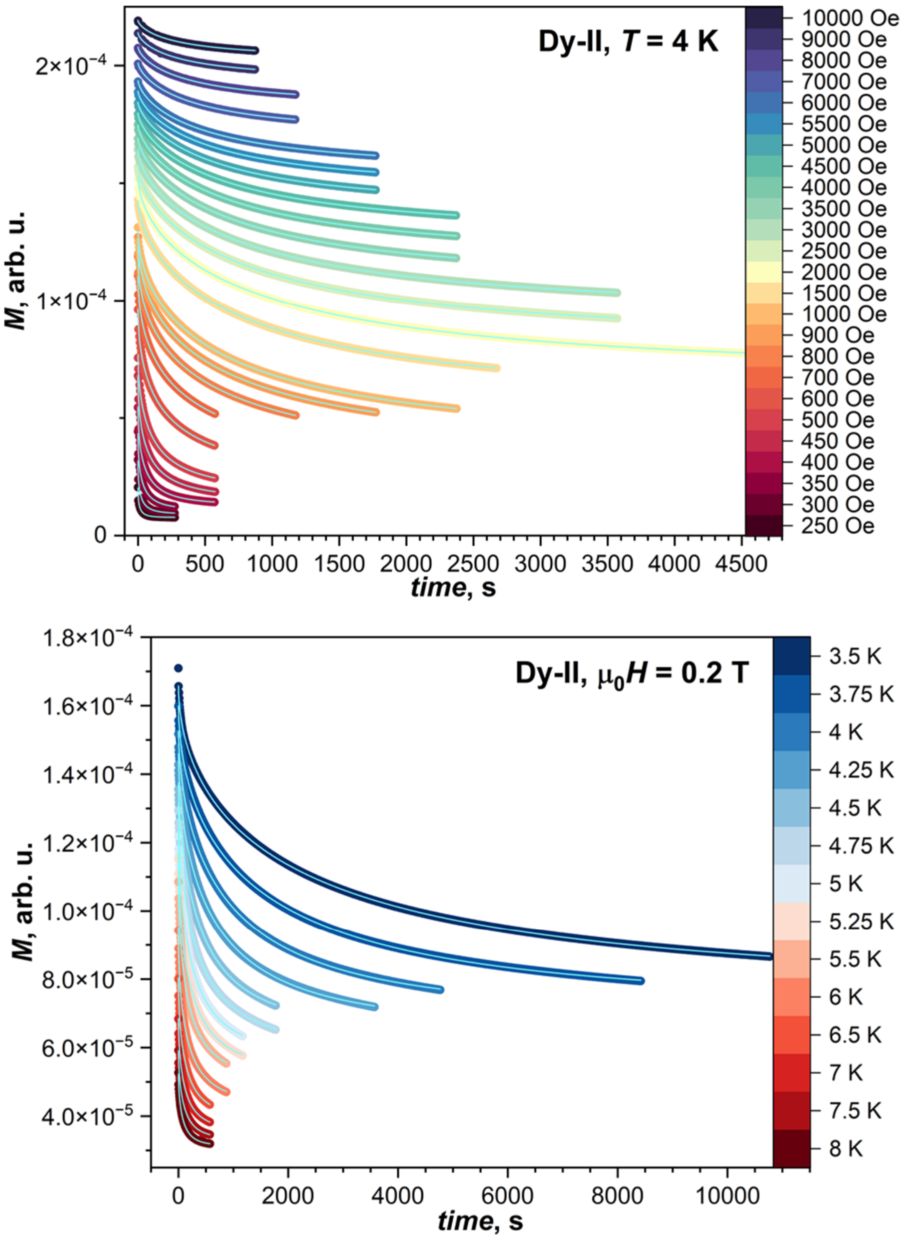

Figure S20. Magnetization decay curves (colored dots) and their fitting with stretched exponential function (cyan curves) for **Dy-II**. Top panel: temperature is fixed to 4 K, magnetic field is varied. Bottom panel: magnetic field is fixed to 0.2 T, temperature is varied.

References

1. Ye, Q.; Komarov, I. V.; Kirby, A. J.; Jones, M. 3,5,7-Trimethyl-1-azatricyclo[3.3.1.13,7]decan-2-ylidene, an Aminocarbene without π Conjugation. *J. Org. Chem.* **2002**, *67* (26), 9288-9294.
2. Schneider, Y.; Prévost, J.; Gobin, M.; Legault, C. Y. Diazirines as Potent Electrophilic Nitrogen Sources: Application to the Synthesis of Pyrazoles. *Org. Lett.* **2014**, *16* (2), 596-599.
3. Laikov, D. N.; Ustynuk, Y. A. PRIRODA-04: a quantum-chemical program suite. New possibilities in the study of molecular systems with the application of parallel computing. *Russ. Chem. Bull.* **2005**, *54* (3), 820-826.
4. Laikov, D. N. Fast evaluation of density functional exchange-correlation terms using the expansion of the electron density in auxiliary basis sets. *Chem. Phys. Lett.* **1997**, *281*, 151-156.
5. Neese, F. Software update: the ORCA program system, version 4.0. *WIREs Comput. Mol. Sci.* **2018**, *8* (1), e1327.
6. Hafner, J. Ab-initio simulations of materials using VASP: Density-functional theory and beyond. *J. Comput. Chem.* **2008**, *29* (13), 2044-2078.
7. Kresse, G.; Hafner, J. Ab initio molecular dynamics for liquid metals. *Phys. Rev. B* **1993**, *47* (1), 558-561.
8. Kresse, G.; Joubert, D. From ultrasoft pseudopotentials to the projector augmented-wave method. *Phys. Rev. B* **1999**, *59* (3), 1758-1775.
9. Perdew, J. P.; Burke, K.; Ernzerhof, M. Generalized gradient approximation made simple. *Phys. Rev. Lett.* **1996**, *77* (18), 3865-3868.
10. Grimme, S. Density functional theory with London dispersion corrections. *WIREs Comput. Mol. Sci.* **2011**, *1* (2), 211-228.
11. Aquilante, F.; Autschbach, J.; Baiardi, A.; Battaglia, S.; Borin, V. A.; Chibotaru, L. F.; Conti, I.; Vico, L. D.; Delcey, M.; Galván, I. F.; et al. Modern quantum chemistry with [Open]Molcas. *J. Chem. Phys.* **2020**, *152* (21), 214117.
12. Chibotaru, L. F.; Ungur, L. Ab initio calculation of anisotropic magnetic properties of complexes. I. Unique definition of pseudospin Hamiltonians and their derivation. *J. Chem. Phys.* **2012**, *137* (6), 064112.
13. Bleaney, B. Nuclear Magnetic Resonance Shifts in Solution Due to Lanthanide Ions. *J. Magn. Reson.* **1972**, *8*, 91-100.
14. Bleaney, B.; Dobson, C. M.; Levine, B. A.; Martin, R. B.; Williams, R. J. P.; Xavier, A. V. Origin of Lanthanide Nuclear Magnetic Resonance Shifts and Their Uses. *J. Chem. Soc., Chem. Commun.* **1972**, 791-793.
15. Yamada, M.; Abe, T.; Saito, C.; Yamazaki, T.; Sato, S.; Mizorogi, N.; Slanina, Z.; Uhlik, F.; Suzuki, M.; Maeda, Y.; et al. Adamantylidene Addition to $M_3N@I_h$-C_{80} (M = Sc, Lu) and $Sc_3N@D_{5h}$-C_{80}: Synthesis and Crystallographic Characterization of the [5,6]-Open and [6,6]-Open Adducts. *Chem.-Eur. J.* **2017**, *23* (27), 6552-6561.
